# Global, regional, and national time trends in cancer mortality attributable to high fasting plasma glucose: an age-period cohort analysis

**DOI:** 10.1186/s12889-023-16076-x

**Published:** 2023-07-15

**Authors:** Jing Xie, Zeye Liu, Liqun Ren, Liyun He, Shan Lu, Xiangzhi Meng, Xin Zhang, Zhanhao Su, Shenqi Jing, Tao Shan, Junjie Wang, Ruibing Xia, Wei Feng, Yakun Li, Naifeng Liu, Yun Liu

**Affiliations:** 1 Department of Pharmacy, Zhongda Hospital, School of Medicine, Southeast University, Nanjing, China; 2 National Center for Cardiovascular Disease, Fuwai Hospital, Chinese Academy of Medical Sciences & Peking Union Medical College, Beijing, 100037 China; 3 Department of Gerontology, Zhongda Hospital, School of Medicine, Southeast University, Nanjing, Jiangsu China; 4 Department of Endocrinology, Key Laboratory of Endocrinology of National Health Commission, Peking Union Medical College Hospital, Chinese Academy of Medical Sciences & Peking Union Medical College, Beijing, China; 5 Department of Outpatient, The First Affiliated Hospital, Nanjing Medical University, Nanjing, China; 6 Department of Thoracic Surgical Oncology, National Cancer Center/Cancer Hospital, Chinese Academy of Medical Sciences and Peking Union Medical College, Beijing, 100021 China; 7 Department of Information, The First Affiliated Hospital of Nanjing Medical University, Nanjing, China; 8 Guangdong Cardiovascular Institute, Guangdong Provincial People’s Hospital, Guangdong Academy of Medical Sciences, Guangzhou, China; 9 Department of Medical Informatics, School of Biomedical Engineering and Informatics, Nanjing Medical University, Nanjing, China; 10 Department of Medicine, University Hospital Munich, Ludwig-Maximilians-University Munich (LMU), Munich, Germany; 11 Laboratory of Experimental Intensive Care and Anesthesiology, Academic Medical Center, Amsterdam, The Netherlands; 12 Department of Cardiology, Zhongda Hospital, School of Medicine, Southeast University, Nanjing, China

**Keywords:** Cancer, high fasting plasma glucose, Mortality, Age-period- cohort

## Abstract

**Background:**

High fasting plasma glucose (HFPG) is the fastest-growing risk factor for cancer deaths worldwide. We reported the cancer mortality attributable to HFPG at global, regional, and national levels over the past three decades and associations with age, period, and birth cohort.

**Methods:**

Data for this study were retrieved from the Global Burden of Disease Study 2019, and we used age-period-cohort modelling to estimate age, cohort and period effects, as well as net drift (overall annual percentage change) and local drift (annual percentage change in each age group).

**Results:**

Over the past 30 years, the global age-standardized mortality rate (ASMR) attributable to HFPG has increased by 27.8%. The ASMR in 2019 was highest in the male population in high sociodemographic index (SDI) areas (8.70; 95% CI, 2.23–18.04). The net drift for mortality was highest in the female population in low SDI areas (2.33; 95% CI, 2.12–2.55). Unfavourable period and cohort effects were found across all SDI quintiles. Cancer subtypes such as "trachea, bronchus, and lung cancers", "colon and rectal cancers", "breast cancer" and "pancreatic cancer" exhibited similar trends.

**Conclusions:**

The cancer mortality attributable to HFPG has surged during the past three decades. Unfavourable age-period-cohort effects on mortality were observed across all SDI quintiles, and the cancer mortality attributable to HFPG is expected to continue to increase rapidly in the future, particularly in lower SDI locations. This is a grim global public health issue that requires immediate attention.

**Supplementary Information:**

The online version contains supplementary material available at 10.1186/s12889-023-16076-x.

## Introduction

High fasting plasma glucose (HFPG) is an abnormal metabolic state, and one of the most common manifestations is diabetes. Studies have shown that HFPG and diabetes are correlated with a variety of malignancies, such as colorectal, breast, and pancreatic cancers, affecting the progression of diseases and increasing mortality [1–7]. These malignancies might be associated with common risk factors, such as hyperglycaemia, insulin resistance, and oxidative stress [8]. Therefore, HFPG is likely to increase the global burden of cancer-related diseases.

It has been described that the cancer burden was associated with HFPG in a large number of studies [9, 10]. However, these reports do not identify the age, period and birth cohort impacts on mortality. Moreover, there are significant differences in disease characteristics and survival among cancer types of various origins. Further analysis of cancer subtypes was required to obtain relevant information on the epidemic trend and to determine the priority direction of medical resource investment. Therefore, it is necessary to perform in-depth analysis of the cancer mortality attributable to HFPG. The effects of age, period, and birth cohort may contribute to the cancer mortality attributable to HFPG because of changes in physiological age, socioeconomic status, lifestyle factors, and treatment strategies [11, 12].

In this regard, analysing trends with particular attention to age, period, and birth cohort effects can promote a deeper understanding of the development of cancer mortality attributable to HFPG, leading to effective policies, improved quality of life, and reduced mortality in cancer patients. The Global Burden of Diseases, Injuries, and Risk Factors Study (GBD 2019) uses a consistent methodology and all available population-level data to generate population health metrics, which provides a novel opportunity for global-scale evaluation of disease trends. Based on the GBD data, we used age-period-cohort (APC) models to explore the changes in cancer mortality attributable to HFPG at global, regional, and national levels from 1990 to 2019, The use of APC models provides insights into the contribution of age-related biological factors, as well as technological and social aspects, to disease trends, which is difficult to achieve through traditional methods of epidemiological analysis [13]. This study was performed as part of the GBD Collaborator Network and in accordance with GBD protocols.

## Methods

### Epidemiological analysis of GBD data

#### Data source

The GBD study used deidentified data, and the informed consent form was approved by the Institutional Review Committee of the University of Washington. The GBD 2019 edition provides the latest estimates of descriptive epidemiological data for a total of 369 diseases and injuries in 204 countries and territories over 30 years from 1990 to 2019 [14, 15]. The GBD network uses standardized tools within the Bayesian framework and all available data across age, time, geography and health causes to generate disease estimates. This approach used the information from available data to estimate the burden of the disease in countries that did not have primary data sources, thereby allowing estimation of the burden of the disease for all regions of the world. In GBD, high fasting plasma glucose (HFPG) was defined as a level of fasting plasma glucose above the theoretical minimum-risk exposure level (TMREL) (4.8–5.4 mmol/L). HFPG was measured as the mean FPG in a population which also include those with a history of diabetes treatment by statistical calculation, and the TMREL was calculated by taking the person-year weighted average of the levels of FPG that were associated with the lowest risk of mortality in the pooled analyses of prospective cohort studies [16]. Cancer death is defined as a death resulting from malignant neoplasms. In GBD, data on cancer deaths were collected from various sources, such as cancer registry data, vital registration (VR), and verbal autopsy (VA) data. The underlying cause of cancer death in GBD 2019 was determined based on the ICD codes and standardized classification rules [16]. The outcome of this research is the cancer mortality attributable to HFPG, and quantified by the comparative risk assessment (CRA) framework in GBD 2019 [16].

The sociodemographic index (SDI), a composite indicator of per capita income, average years of schooling in the population older than 15 years, and the fertility rate for females under the age of 25 years, was utilized by the analysis [14]. The SDI ranged from 0 to 1, with increased values indicating elevated socioeconomic levels, and all countries were evenly divided into five categories according to the quintile of SDI in 2019. The GBD database provided data on high-incidence tumour types in male and female populations.

### Overall time trend analysis of cancer mortality attributable to HFPG

The time trend in mortality in the study was assessed by the age-standardized rate as well as the percentage change in the age-standardized mortality rate (ASMR) from 1990 to 2019. In GBD, age-standardized rate for death were computed using the GBD world population age standard [14]. To predict the number of cancer deaths attributable to HFPG in the following years, we used a Bayesian age-period-cohort (BAPC) model. In the BAPC model, we assumed that the data were inverse gamma prior, adjusting for excessive dispersion in terms of age, period, and cohort effects [17]. Moreover, To visualize the proportion of cancer deaths in different age groups, we used the age groupings of 25–49, 50–59, 60–69, 70–79, and 80 + to create a bar chart.

### Age-period-cohort model analysis

The age-period-cohort (APC) model was used to analyse potential trends in mortality by age, period, and birth cohort [18]. Net drift and local drift are important parameters in the APC model. Net drift represents an overall log-linear trend by period and birth cohort, indicating an overall annual percentage change in the expected age adjustment rate over time. Local drift is a log-linear trend for each age group by period and birth group, indicating the expected annual percentage change in age-specific rates over time. Age effects are expressed as longitudinal age curves to represent the age-associated natural history of mortality attributable to HFPG. Period effects are expressed as the relative risk of mortality by period and are used to track progress in different time periods. Cohort effects are expressed as the relative risk of mortality by cohort and are used to track changes in mortality for different birth cohorts. More APC-specific methods and details are available in existing publications [13].

The input data for the APC model in the study were the population data of each country/territory from 1990 to 2019 and the estimated cancer mortality attributable to HFPG. In the APC model, all the age and period intervals must be equal. Therefore, the death population data in the study were divided into consecutive five-year periods from 1990 to 2019 (1990–1994 [1992], 1995–1999 [1997]…2015–2019 [2017]), with the survey years from 2000 to 2004 as the reference period group. As the number of deaths under 50 and over 90 years was too small, the age intervals for five consecutive years were 50–54 years, 55–59 years…80–84 years, and 85–89 years. The sample consisted of 13 consecutive cohorts, comprising cohorts born during 1903–1907 (median, 1905) and 1963–1967 (median, 1965), with cohorts born during 1933–1937 (median, 1935) serving as the reference group. The APC model analysis used the age-period-cohort Web Tool provided by the National Cancer Institute [19]. The significance of the annual change percentage trend was evaluated using the Wald chi-squared test, executed through R code on the age-period-cohort Web Tool. More information about the web tool are available in the literature [19]. Statistical testing was conducted in both directions and considered significant when *P* < 0.05. All the figures related to the APC models were performed using R software(version 4.2.2).

## Results

### Rapidly increasing risk factors for cancer mortality worldwide, 1990–2019

**Fig. 1:**
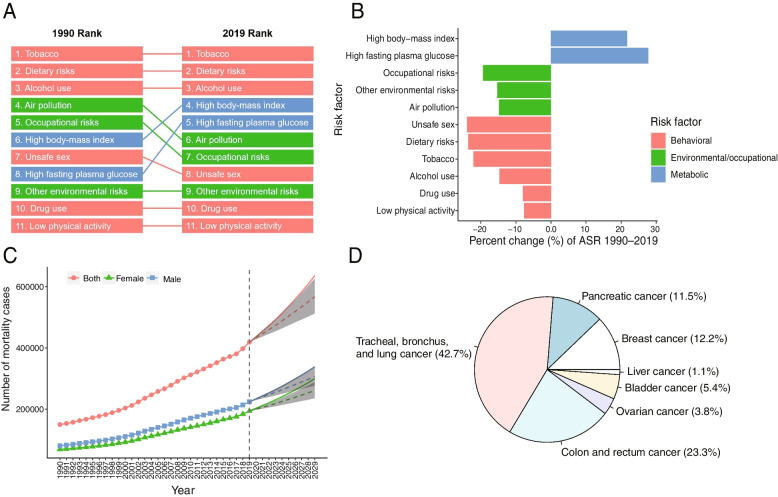
Rapid causes of cancer mortality worldwide, 1990–2019. **A** Risk factors ranking for age-standardized cancer death rates for the global, 1990–2019; (**B**) Percent change of risk factors contributing to age-standardized cancer death rates for the global, 1990—2019; (**C**) Prediction of the number of cancer deaths attributable to HPFG from 2020 to 2029 for the global; (**D**) Proportions of cancer deaths attributable to HFPG by subtype for the global in 2019

The number of cancer deaths attributable to HFPG worldwide in 2019 was 419,340 (95% CI 115,730–848,480). In the global range, tobacco consumption, dietary risks, and alcohol consumption (top three from high to low) were still risk factors that caused the most cancer deaths in 2019 when compared with 1990. However, the number of cancer deaths attributable to HFPG rose from the eighth (1990) to the fifth rank (2019) (Fig. 1A), the largest increase among all risk factors (Fig. 1B), followed by high body-mass index.

Its percentage increase of ASMR attributable to HFPG in the whole population over the past 30 years was 27.8%, 28.1% higher than that of the second risk factor (the increased percentage of ASMR attributable to high body-mass index in the past 30 years was 21.7%). For the female population, in particular, the percentage increase of ASMR was 175.3% higher than that of the second risk factor (Supplement Table S1, Figure S1-A). Based on the BAPC model, the number of cancer deaths attributable to HFPG was predicted to continue to rise to 636,166 by 2029 (Fig. 1C). The top four cancer subtypes were "trachea, bronchus and lung cancer", "colon and rectal cancer", "breast cancer" and "pancreatic cancer", accounting for 89.7% of the total number (Fig. 1D). It should be noted that the cancer types corresponding with the most deaths in females were different from those in males. The most common cancers in males were “trachea, bronchus, and lung cancers”, “colon and rectal cancer” and “pancreatic cancer”. Among females, the top three were “trachea, bronchus and lung cancer”, “breast cancer” and “colon and rectal cancer”. Among the cancer subtypes, breast cancer accounted for a large proportion (26.3%) in females (Supplement Figure S1-B).

### Global and regional trends in cancer mortality attributable to HFPG, 1990–2019

**Fig. 2:**
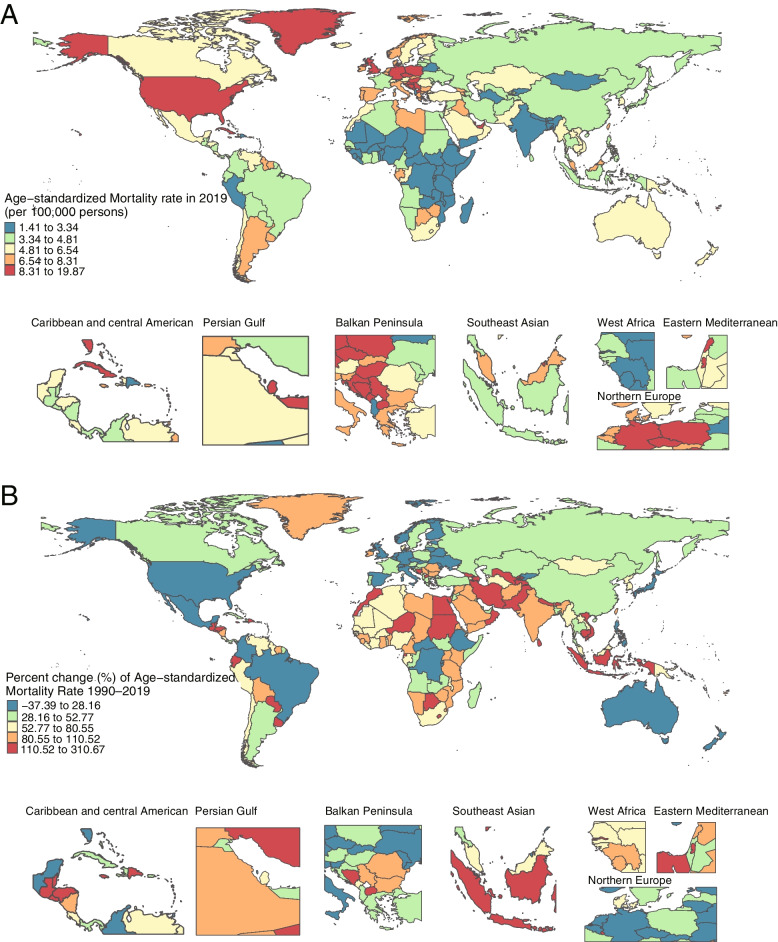
The age-standardized rate in 2019 (**A**) and percent change (%) of the age-standardized rate during 1990–2019 (**B**) for cancer mortality attributable to HFPG

The age-standardized rate of mortality in 2019 (total of 204 countries), as well as the percent change from 1990 to 2019, are shown in Fig. 2. Table 1 shows the population, the total number of deaths, the all-age rate, the age-standardized rate, and the net drift for mortality. From 1990 to 2019, the global population increased from 5.35 billion (95% CI, 5.24–5.46) to 7.74 billion (95% CI, 7.48–7.99), representing an increase of 44.67%. However, the number of cancer deaths attributable to HFPG increased from 150.10 thousand (95% CI, 39.21–312.37) to 419.34 thousand (95% CI, 115.73–848.48), an increase of 179.37%, more than four times the global population growth rate. The mortality rate in all SDI areas showed an upward trend, especially in middle, low middle and low SDI areas. In 2019, the age-standardized mortality rate in the total population ranged from 7.22 (95% CI, 2.04–14.45) in the high SDI areas to 2.64 (95% CI, 0.72–5.49) in the low SDI areas (Table 1). The highest mortality for males was found in the high SDI areas, which was 8.70 (95% CI, 2.23–18.04) (Supplement Table S2).

**Table 1:** Trends in cancer mortality attributable to HPFG for both genders across SDI quintiles, 1990 − 2019

	**Global**		**High SDI**		**High-middle SDI**		**Middle SDI**		**Low-middle SDI**		**Low SDI**	
	**1990**	**2019**	**1990**	**2019**	**1990**	**2019**	**1990**	**2019**	**1990**	**2019**	**1990**	**2019**
Population												
Number, n × 1,000,000	5350 (5239,5460)	7737 (7483,7993)	822	1013	1150	1430	1717	2397	1130	1764	528	1128
Percentage of global, %	100	100	15.4	13.1	21.5	18.5	32.1	31.0	21.1	22.8	9.9	14.6
Deaths												
Number, n × 1,000	150.10 (39.21,312.37)	419.34 (115.73,848.48)	67.83 (18.04,138.40)	145.81 (41.55,291.37)	44.42 (11.48,92.55)	113.97 (31.12,233.27)	25.28 (6.54,53.15)	104.15 (27.98,216.01)	9.35 (2.41,20.03)	43.17 (11.95,88.81)	3.13 (0.81,6.75)	11.98 (3.25,25.13)
Percentage of global, %	100	100	45.19	34.77	29.59	27.18	16.84	24.84	6.23	10.29	2.09	2.86
Percent change of deaths 1990–2019, %	179.37 (161.95,203.95)		114.96 (102.66,136.03)		156.58 (137.91,182.93)		311.95 (269.40,368.06)		361.81 (311.88,437.15)		282.43 (239.35,339.78)	
All-age mortality rate												
Rate per 100,000	2.81 (0.73,5.84)	5.42 (1.50,10.97)	8.25 (2.19,16.84)	14.39 (4.10,28.75)	3.86 (1.00,8.04)	7.97 (2.18,16.31)	1.47 (0.38,3.1)	4.35 (1.17,9.01)	0.83 (0.21,1.77)	2.45 (0.68,5.03)	0.59 (0.15,1.28)	1.06 (0.29,2.23)
Percent change of rate 1990–2019, %	93.16 (81.12,110.16)		74.37 (64.39,91.46)		106.36 (91.35,127.55)		195.1 (164.61,235.29)		195.74 (163.77,243.99)		78.95 (58.79,105.78)	
Age-standardized mortality rate												
Rate per 100,000	4.11 (1.08,8.48)	5.26 (1.45,10.62)	6.35 (1.69,12.98)	7.22 (2.04,14.45)	4.34 (1.13,9.01)	5.59 (1.52,11.42)	2.80 (0.74,5.87)	4.49 (1.21,9.29)	1.79 (0.47,3.81)	3.4 (0.95,6.96)	1.57 (0.41,3.36)	2.64 (0.72,5.49)
Percent change of rate 1990–2019, %	27.78 (20.49,38.66)		13.61 (7.71,24.14)		28.6 (19.35,40.82)		60.22 (44.14,80.88)		89.45 (68.50,119.50)		67.86 (49.84,92.94)	
APC model estimates												
Net drift of mortality y, % per year	0.90 (0.85,0.94)		0.52 (0.47,0.56)		0.88 (0.82,0.94)		1.62 (1.53,1.72)		2.09 (1.99,2.18)		1.75 (1.61,1.90)	

The net drift for mortality ranged from 0.52% (95% CI, 0.47–0.56) per year in areas with high SDI to 1.75% (95% CI, 1.61–1.90) per year in areas with low SDI between 1990 and 2019 (Table 1). The net drift of mortality in low SDI areas for females was the highest, reaching 2.33% (95% CI, 2.12–2.55) (Supplement Table S2). Meanwhile, the results of global and regional trend analyses of mortality for specific cancer subtypes exhibited a similar trend, as shown in Supplemental Tables S3-S6.

### National trends in cancer mortality attributable to HFPG, 1990–2019

Among the 204 countries and territories worldwide, China (90,655 deaths; 95% CI, 23,078–197,161), the United States of America (58,134 deaths; 95% CI, 16,747–114,100), India (30,220 deaths; 95% CI, 8,320–62,897) and Germany (19,416 deaths; 95% CI, 5,634–38,269) were the top four countries in terms of cancer deaths. The top four countries accounted for 47.3% of global deaths. At the same time, 150 countries and territories showed annual net drift ≥ 1.0%. An annual net drift increase of 1% means that the number of deaths would increase by 10%, 18% and 26% in the next 10, 20 and 30 years, respectively [19]. Among 204 countries and territories, Uzbekistan at 4.56% (95% CI: 3.79–5.33), Georgia at 4.52% (95% CI: 3.82–5.24), Lesotho at 4.36% (95% CI: 2.46–6.29), Cabo Verde at 4.29% (95% CI: 0.02–8.73), and Egypt at 4.24% (95% CI: 3.80–4.68) were the top five countries with the fastest increase (Supplement Table S7).

In general, these results suggest that the overall disease burden is increasing in most countries and territories of the world. Countries and territories with higher levels of SDI generally had higher mortality, yet the numbers increased relatively slowly. Although most of the countries and territories with low SDI levels had relatively low mortality, the numbers were rapidly increasing.

**Fig. 3:**
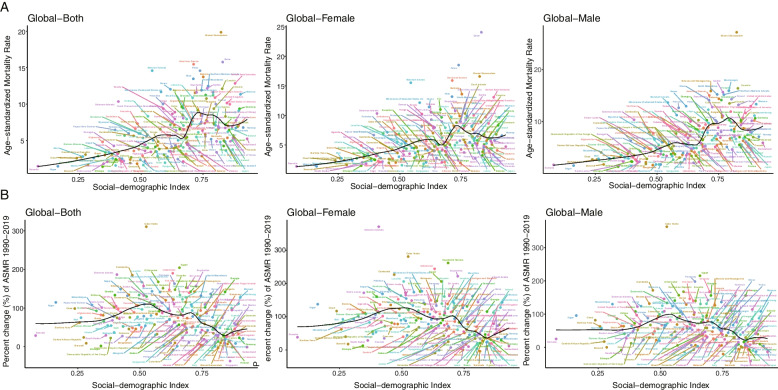
The age-standardized mortality rates in 2019 (**A**) and percent change (%) in the age-standardized rate during 1990–2019 (**B**) for 204 countries and territories by sociodemographic index

The age-standardized mortality rates and the percentage changes with SDI level in a total of 204 countries and territories are shown in Fig. 3. Countries with high SDI levels have higher age-standardized mortality rates, while countries with low SDI levels have higher percent change values. The specific cancer subtypes exhibited similar trends, as shown in Supplemental Figures S2-5.

### Time trends of distribution in mortality attributable to HFPG among different age groups

**Fig. 4:**
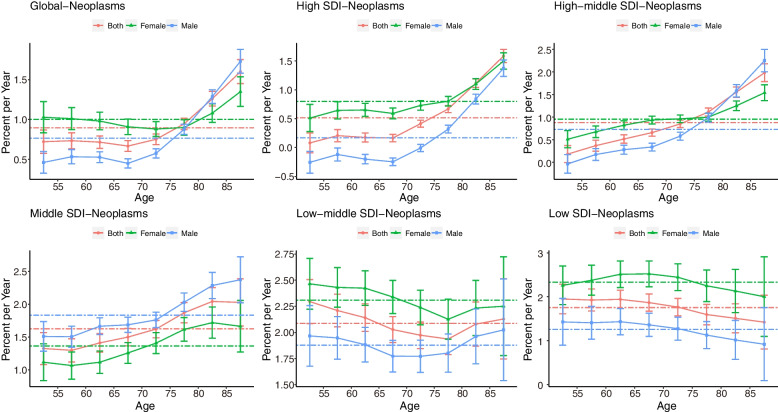
Local drifts of cancer mortality attributable to HFPG by SDI quintiles, 1990–2019

Figure 4 shows the net drift and local drifts for global and SDI quintile regions. Higher net drift values were found in lower SDI regions. Globally, cancer mortality attributable to HFPG showed increasing trends across all age groups (p < 0.05), and the trend intensified with age. The largest mortality increase occurred in the 84–89 years group, and the local drift value was 1.60% (95% CI: 1.45–1.75). The mortality rate of females increased faster than that of males globally. In high-, high-middle-, and middle-SDI regions, older people experienced a faster mortality increase, while in low-middle- and low-SDI regions, younger people experienced a faster mortality increase. Tracheal, bronchus, and lung, breast and pancreatic cancer had similar trends to neoplasm, while colon and rectal cancer in the 50–55 and 85–89 year groups had a relatively high death rate increase, as shown in Supplemental Figures S6-9.

**Fig. 5:**
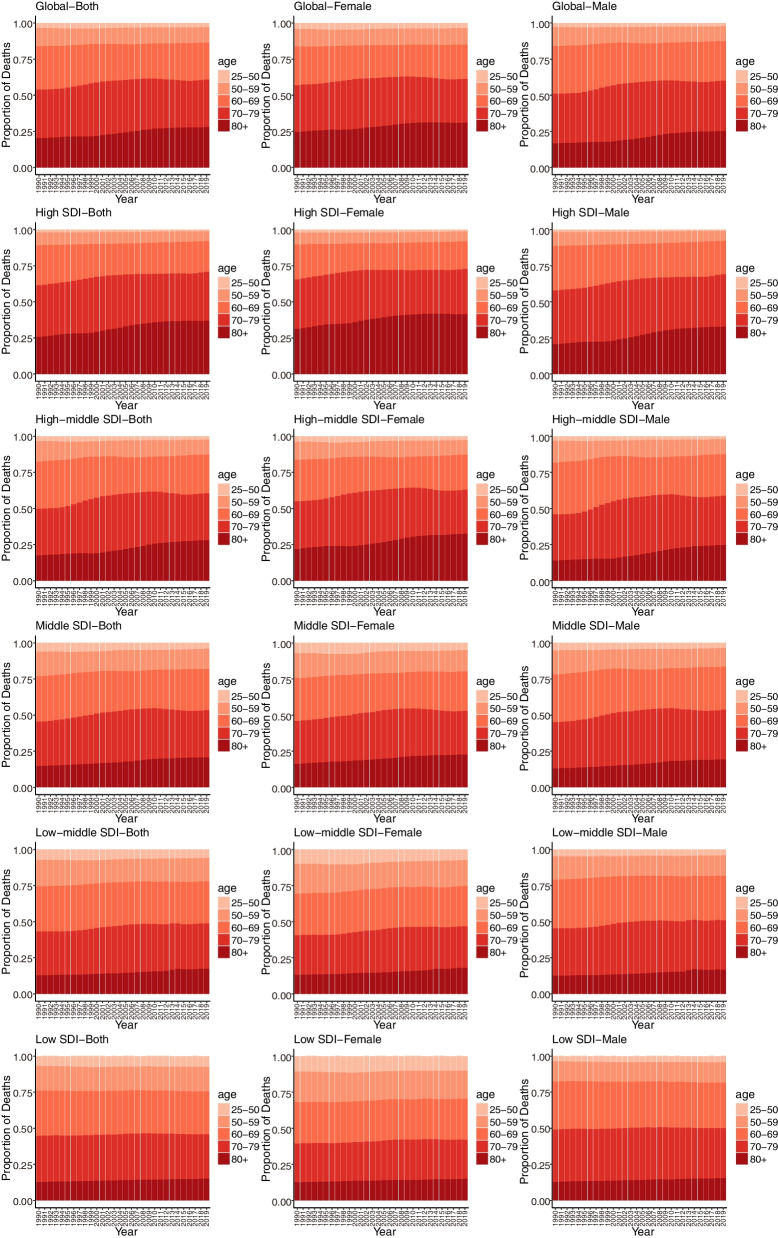
Age distribution of cancer deaths attributable to HFPG by SDI quintiles, 1990–2019

Figure 5 shows the time trend of deaths with the age distribution, which represents the survival status for cancer. From a global perspective, the time trend of deaths in the elderly population (80 + years) gradually increased and did not differ by gender. This trend was clearer in countries with high and high-middle SDI levels. It was noted that the number of deaths below 80 years was still in the minority in countries with middle, low-middle and low SDI, which might be related to the short average lifespan in these countries. Death according to cancer subtypes also exhibited the same trend, as shown in Supplemental Figures S10-13.

### Age-period-cohort effects on mortality attributable to HFPG

**Fig. 6:**
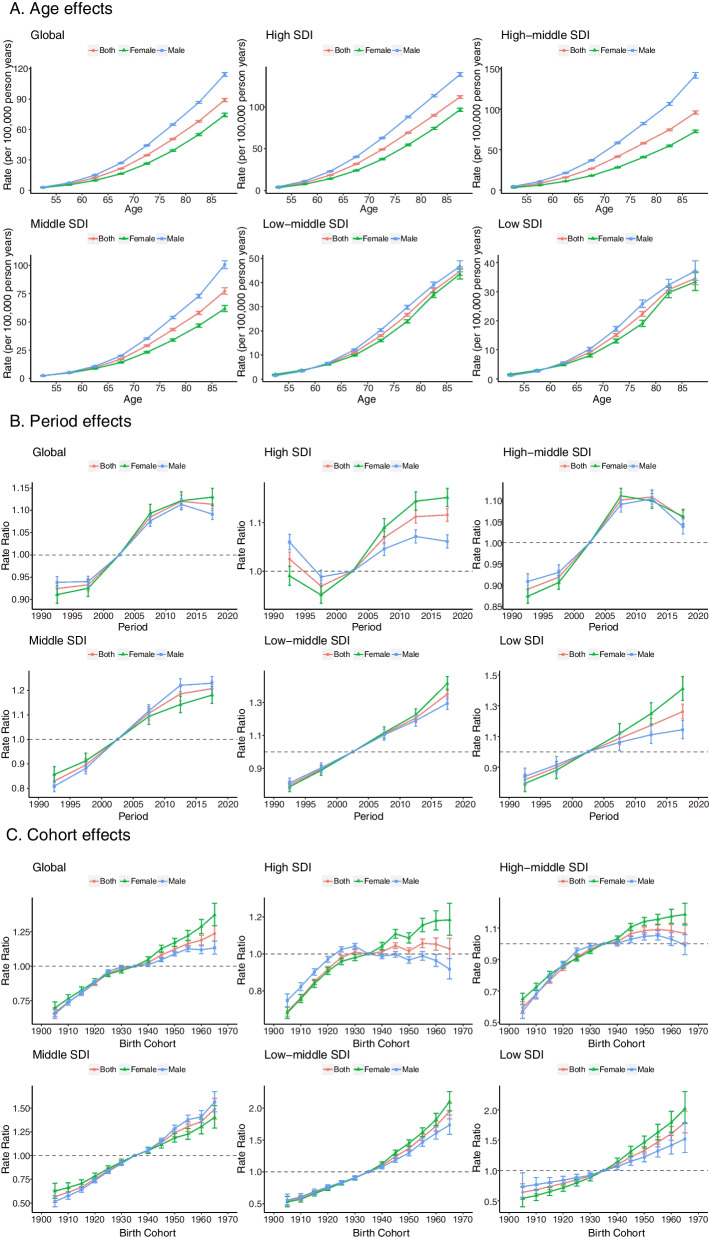
Age, period and cohort effects on cancer mortality attributable to HFPG by SDI quintiles

The age, period and cohort effects estimated by the APC model for mortality across SDI quintiles are shown in Fig. 6. A similar age-effect pattern was found across countries and territories with different SDI levels. Elderly individuals aged 85 to 89 had the highest risk, which gradually increased with age from 50 to 89 years old. The mortality of all age groups was generally higher in countries with high SDI and high-middle SDI. In addition, the risk of death in males was higher than that in females across SDI quintiles. Although an increase in mortality was observed across SDI quintiles, the mortality of females in low-middle and low SDI areas rapidly increased compared with that of males.

There were differences in the period effects across different SDI quintiles. In terms of global trends, the risk of mortality declined in the last decade, especially in high-middle and high SDI regions. However, a sustained upward trend was observed in the middle, low-middle and low SDI regions.

Globally, the mortality risk of successive younger birth cohorts also showed a trend of differences among SDI quintiles. Similar to the period effect, the global risk of mortality increased with the birth cohort, especially in middle, low-middle and low SDI regions. However, it was noteworthy that the mortality risk of males in high and high-middle SDI regions declined (birth cohort after 1935).

The effects of age, period, and birth cohort on death in cancer subtypes are shown in Supplemental Figures S14-17. Trends in disease burden for “tracheal, bronchial, lung cancer”, “colon, rectal cancer”, and “breast cancer” were generally consistent with the overall trend. For “pancreatic cancer”, there was a more pronounced upward trend in period and birth cohort effects for all SDI areas.

### Age-period-cohort effects in representative countries

**Fig. 7:**
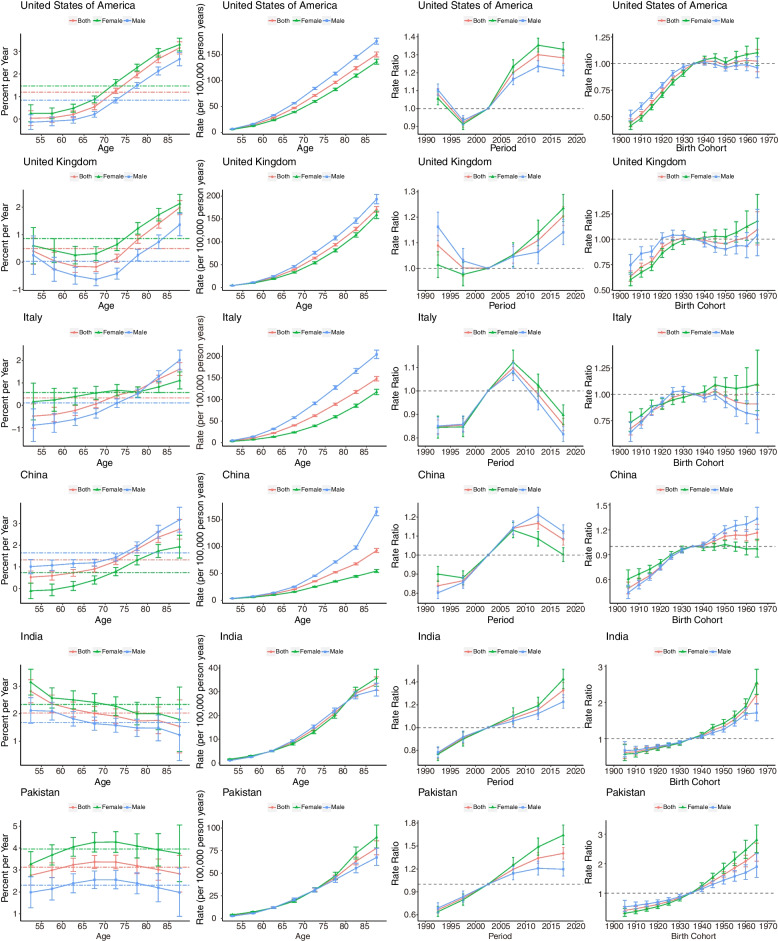
Age, period and cohort effects on neoplasm mortality attributable to HFPG for representative countries

Unfavourable age-period-cohort effects were observed across SDI quintiles. We selected representative countries from the five SDI levels (Fig. 7) to better describe the main trend in mortality through age-period-cohort effects across the world. The net drifts from six countries increased during the past 30 years. Notably, the net drift value for males was higher than that for females in China, where the drift (95% CI) values for males and females were 1.65 (1.52, 1.78) and 0.74 (0.60, 0.88), respectively. However, the net drift (95% CI) values for males in the United Kingdom and Italy populations were 0.03 (-0.14, 0.20) and 0.11 (-0.06, 0.27), respectively, which indicated that there was no significant increase in mortality (Supplement Table S8). Regarding the age effect, increased mortality was observed in all age groups for the six countries, which was more significant in males. In terms of the period effect, Italy was different from other countries and showed a clear downward trend. In terms of the birth cohort effect, the increasing trend slowed after 1935 for populations in the United States of America, United Kingdom, Italy, and China. However, increasing trends were observed in India and Pakistan. The age-period-cohort effects of cancer subtypes in representative countries are shown in Supplemental Figures S18-21 and Table S9-12.

## Discussion

Cancer is a major public health problem that all mankind is facing [20], and abnormal glucose metabolism increases the risk of death. In the past 30 years, the global population has increased by 44.67%, yet cancer deaths attributable to HFPG have increased by 179.37%, approximately four times the rate of population increase. This is a grim public health issue globally that requires our immediate attention.

In this study, the APC model was first applied to analyse the time trends of cancer mortality attributable to HFPG. Our results indicated that age, period, and birth cohort are influential factors underlying the increasing mortality trend. The local drift curves indicated that mortality attributable to HFPG increased with age in higher SDI locations, with older people experiencing a more rapid increase. This may be due to population ageing in these locations [21, 22]. In lower SDI locations, younger people had a greater mortality increase, showing a trend towards younger age of cancer mortality attributable to HFPG. The longitudinal age curves showed that mortality increased with age, which may be related to the degeneration of body functions. The period relative risk curves showed an upward trend, which may be associated with dietary and lifestyle changes over time. In higher SDI locations, mortality decreased or slowed in the last decade, possibly due to improved medical technology and health awareness. Conversely, lower SDI locations experienced a rapid mortality increase over the past 30 years. This could be related to limited access to health care and lack of awareness, resulting in many cancer patients being unable to afford better treatment [23–25]. These findings highlight the urgent need to increase awareness of blood glucose screening and control in cancer patients in lower SDI locations, as well as to improve the effectiveness of relevant therapies in the future. Globally, recent birth cohorts have a higher risk of cancer mortality attributable to high fasting plasma glucose, particularly in lower SDI locations. This increase is likely linked to rapid urbanization and industrialization over the past few decades, resulting in major lifestyle changes such as sedentary behaviour and unhealthy diets, which have led to a rapid increase in blood glucose levels [26–30]. Hyperglycaemia can facilitate tumourigenesis and enhance cancer development by promoting tumour cell proliferation, invasion, and migration, as well as inducing apoptosis resistance and chemoresistance [31–33]. With global population ageing [34] and recent birth cohorts continuing to exhibit increasing high-risk behaviours, the risk of cancer mortality attributable to HFPG is expected to continue to rise. Without timely prevention and control, this trend will result in significant disease and economic burdens, especially in lower SDI locations.

Compared to previous publications, our research makes significant contributions to better understanding the reasons underlying the rapid increase in cancer mortality attributable to HFPG over the past 30 years. In addition, we found that males in high SDI regions had higher age-standardized mortality rates, while females in lower SDI regions showed a rapid increase, indicating a potential increase in disease burden for females in countries with lower SDI levels. The reasons for the differences may be related to lifestyle, health education, health care resources, and cancer atlases of gender [35, 36]. The most common cancers in males were “trachea, bronchus, and lung cancer”, “colon and rectal cancer” and “pancreatic cancer”, which may be related to the cancer incidence and the association between high fasting plasma glucose and cancer. According to the World Cancer Research Fund (WCRF) [37],The top three common cancers in males worldwide were lung, prostate and colorectal cancers, accounting for 41.9% of all cancers (excluding nonmelanoma skin cancer). Previous studies have shown that prostate cancer may have an inverse association with diabetes or hyperglycaemia [8, 38, 39]. Therefore, despite its high incidence, there was no data about the burden of prostate cancer attributable to HFPG in the GBD 2019. While pancreatic cancer has a relatively low incidence (2.8% of all cancers), the high mortality rate of the disease [40, 41] combined with the strong association with diabetes or hyperglycaemia [42] contributes to a relatively high number of pancreatic cancer deaths attributable to HFPG. These factors may explain why "trachea, bronchus, and lung cancers", "colon and rectal cancer", and "pancreatic cancer" were the most common cancers in males. Among females, breast, colorectal and lung cancer are the three most common cancers, comprising a total of 44.5% of all cancers [37]. In previous studies, these types of cancers have also been reported to have a positive association with diabetes or hyperglycaemia [8]. These findings may explain why the most common cancers in females are "trachea, bronchus, and lung cancers", "breast cancer", and "colon and rectal cancer". Our study also revealed a lower increase in cancer mortality attributable to HFPG in Italy, which could be attributable to their traditional Mediterranean diet. The Mediterranean diet has the potential to prevent hyperglycaemia and cancer due to its antioxidant and anti-inflammatory properties [43, 44]. Additionally, our findings indicate a trend towards younger age at cancer mortality attributable to HFPG, particularly among females in India, highlighting the need for special attention to this issue.

This study has several limitations. First, the limitation derived from the GBD model is that the raw data for low- and middle-income countries were limited. Second, the APC model analysed in our study was based on the estimated cross-sectional data of GBD. Cohort studies are needed in the future to establish location- and time-specific relative risks. Third, this study analyses mortality data at the national level and does not present local differences. As the level of development of different regions in countries is often unbalanced, more sophisticated analysis using local data can identify each region trend.

## Conclusions

In summary, the management of glucose levels in cancer survivors should be emphasized in clinical practice, particularly in lower SDI locations. Cancer survivors with HFPG can be identified through glucose monitoring and screening and appropriately treated as early as possible to improve survival. Furthermore, our study provides important evidence for health authorities to achieve better resource allocation and to decrease the cancer burden attributable to HFPG.

## Supplementary Information

**Additional file 1: Figure S1.** Rapid causes of cancer mortality worldwide for females and males, 1990–2019. **Figure S2.** Tracheal, bronchus, and lung cancer age-standardized mortality rates in 2019 (A) and percent change (%) in age-standardized rates during 1990–2019 (B) for 204 countries and territories by sociodemographic index. **Figure S3.** The colon and rectal cancer age-standardized mortality rates in 2019 (A) and percent change (%) in the age-standardized rate during 1990–2019 (B) for 204 countries and territories by sociodemographic index. **Figure S4.** Breast cancer age-standardized mortality rates in 2019 (A) and percent change (%) in age-standardized rates during 1990–2019 (B) for 204 countries and territories by sociodemographic index. **Figure S5.** Pancreatic cancer age-standardized mortality rates in 2019 (A) and percent change (%) in age-standardized rates during 1990–2019 (B) for 204 countries and territories by sociodemographic index. **Figure S6.** Local drifts of tracheal, bronchus, and lung cancer mortality attributable to HFPG by SDI quintiles, 1990–2019. **Figure S7.** Local drifts of colon and rectal cancer mortality attributable to HFPG by SDI quintiles, 1990–2019. **Figure S8.** Local drifts of breast cancer mortality attributable to HFPG by SDI quintiles, 1990–2019. **Figure S9.** Local drifts of pancreatic cancer mortality attributable to HFPG by SDI quintiles, 1990–2019. **Figure S10.** Age distribution of tracheal, bronchus, and lung cancer mortality attributable to HFPG by SDI quintiles, 1990–2019. **Figure S11.** Age distribution of colon and rectal cancer mortality attributable to HFPG by SDI quintiles, 1990–2019. **Figure S12.** Age distribution of breast cancer mortality attributable to HFPG by SDI quintiles, 1990–2019. **Figure S13.** Age distribution of pancreatic cancer mortality attributable to HFPG by SDI quintiles, 1990–2019. **Figure S14.** Age, period and cohort effects on tracheal, bronchus, and lung cancer mortality attributable to HFPG by SDI quintiles. **Figure S15.** Age, period and cohort effects on colon and rectal cancer mortality attributable to HFPG by SDI quintiles. **Figure S16.** Age, period and cohort effects on breast cancer mortality attributable to HFPG by SDI quintiles. **Figure S17**. Age, period and cohort effects on pancreatic cancer mortality attributable to HFPG by SDI quintiles. **Figure S18.** Age, period and cohort effects on tracheal, bronchus, and lung cancer mortality attributable to HFPG for representative countries. **Figure S19.** Age, period and cohort effects on colon and rectal cancer mortality attributable to HFPG for representative countries. **Figure S20.** Age, period and cohort effects on breast cancer mortality attributable to HFPG for representative countries. **Figure S21.** Age, period and cohort effects on pancreatic cancer mortality attributable to HFPG for representative countries. **Table S1.** Percent changes (%) in the age-standardized mortality rate 1990–2019 for all risk factors. **Table S2.** Trends in cancer mortality attributable to HPFG for females and males across SDI quintiles, 1990 − 2019. **Table S3.** Trends in tracheal, bronchus, and lung cancer mortality attributable to HPFG for both females and males across SDI quintiles, 1990 − 2019. **Table S4.** Trends in colon and rectal cancer mortality attributable to HPFG for both females and males across SDI quintiles, 1990 − 2019. **Table S5.** Trends in breast cancer mortality attributable to HPFG for female across SDI quintiles, 1990 − 2019. **Table S6.** Trends in pancreatic cancer mortality attributable to HPFG for both females and males across SDI quintiles, 1990 − 2019. **Table S7.** Trends in cancer mortality attributable to HPFG for both genders in 204 countries and regions, 1990–2019. **Table S8.** Trends in cancer mortality attributable to HPFG for both females and males in representative countries, 1990–2019. **Table S9.** Trends in tracheal, bronchus, and lung cancer mortality attributable to HPFG for both females and males in representative countries, 1990–2019. **Table S10.** Trends in colon and rectal cancer mortality attributable to HPFG for both females and males in representative countries, 1990–2019. **Table S11.** Trends in breast cancer mortality attributable to HPFG for both females in representative countries, 1990–2019. **Table S12.** Trends in pancreatic cancer mortality attributable to HPFG for both females and males in representative countries, 1990–2019.

## Data Availability

The GBD database is available at https://ghdx.healthdata.org/gbd-results-tool.

## Abbreviations

GBD: Global Burden of Diseases, Injuries, and Risk Factors
HFPG: High fasting plasma glucose
ASMR: Age-standardized mortality rate
SDI: Sociodemographic index
APC: Age-period-cohort
BAPC: Bayesian age-period-cohort
TMREL: Theoretical minimum-risk exposure level
