# Supplementary material for: Global, regional, and national time trends in cancer mortality attributable to high fasting plasma glucose: an age-period cohort analysis
Source: BMC Public Health. 2023 Jul 15;23:1361. doi: 10.1186/s12889-023-16076-x (PMC10349485; doi:10.1186/s12889-023-16076-x)
Supplement: Supplementary file 1 — Additional file 1: Figure S1. Rapid causes of cancer mortality worldwide for females and males, 1990–2019. Figure S2. Tracheal, bronchus, and lung cancer age-standardized mortality rates in 2019 (A) and percent change (%) in age-standardized rates during 1990–2019 (B) for 204 countries and territories by sociodemographic index. Figure S3. The colon and rectal cancer age-standardized mortality rates in 2019 (A) and percent change (%) in the age-standardized rate during 1990–2019 (B) for 204 countries and territories by sociodemographic index. Figure S4. Breast cancer age-standardized mortality rates in 2019 (A) and percent change (%) in age-standardized rates during 1990–2019 (B) for 204 countries and territories by sociodemographic index. Figure S5. Pancreatic cancer age-standardized mortality rates in 2019 (A) and percent change (%) in age-standardized rates during 1990–2019 (B) for 204 countries and territories by sociodemographic index. Figure S6. Local drifts of tracheal, bronchus, and lung cancer mortality attributable to HFPG by SDI quintiles, 1990–2019. Figure S7. Local drifts of colon and rectal cancer mortality attributable to HFPG by SDI quintiles, 1990–2019. Figure S8. Local drifts of breast cancer mortality attributable to HFPG by SDI quintiles, 1990–2019. Figure S9. Local drifts of pancreatic cancer mortality attributable to HFPG by SDI quintiles, 1990–2019. Figure S10. Age distribution of tracheal, bronchus, and lung cancer mortality attributable to HFPG by SDI quintiles, 1990–2019. Figure S11. Age distribution of colon and rectal cancer mortality attributable to HFPG by SDI quintiles, 1990–2019. Figure S12. Age distribution of breast cancer mortality attributable to HFPG by SDI quintiles, 1990–2019. Figure S13. Age distribution of pancreatic cancer mortality attributable to HFPG by SDI quintiles, 1990–2019. Figure S14. Age, period and cohort effects on tracheal, bronchus, and lung cancer mortality attributable to HFPG by SDI quintiles. Figure S15. Age [file 12889_2023_16076_MOESM1_ESM.docx]

**Supplementary**

# Table of Contents

[Table of Contents 1](#_Toc24335)

[Figure S1. Rapid causes of cancer mortality worldwide for females and males, 1990-2019 3](#_Toc9834)

[Figure S2. Tracheal, bronchus, and lung cancer age-standardized mortality rates in 2019 (A) and percent change (%) in age-standardized rates during 1990-2019 (B) for 204 countries and territories by sociodemographic index. 4](#_Toc28577)

[Figure S3. The colon and rectal cancer age-standardized mortality rates in 2019 (A) and percent change (%) in the age-standardized rate during 1990-2019 (B) for 204 countries and territories by sociodemographic index. 4](#_Toc26067)

[Figure S4. Breast cancer age-standardized mortality rates in 2019 (A) and percent change (%) in age-standardized rates during 1990-2019 (B) for 204 countries and territories by sociodemographic index. 5](#_Toc16039)

[Figure S5. Pancreatic cancer age-standardized mortality rates in 2019 (A) and percent change (%) in age-standardized rates during 1990-2019 (B) for 204 countries and territories by sociodemographic index. 5](#_Toc12062)

[Figure S6. Local drifts of tracheal, bronchus, and lung cancer mortality attributable to HFPG by SDI quintiles, 1990-2019. 6](#_Toc9551)

[Figure S7. Local drifts of colon and rectal cancer mortality attributable to HFPG by SDI quintiles, 1990-2019. 6](#_Toc684)

[Figure S8. Local drifts of breast cancer mortality attributable to HFPG by SDI quintiles, 1990-2019. 7](#_Toc9913)

[Figure S9. Local drifts of pancreatic cancer mortality attributable to HFPG by SDI quintiles, 1990-2019. 8](#_Toc21074)

[Figure S10. Age distribution of tracheal, bronchus, and lung cancer mortality attributable to HFPG by SDI quintiles, 1990-2019. 9](#_Toc25529)

[Figure S11. Age distribution of colon and rectal cancer mortality attributable to HFPG by SDI quintiles, 1990-2019. 10](#_Toc8356)

[Figure S12. Age distribution of breast cancer mortality attributable to HFPG by SDI quintiles, 1990-2019. 11](#_Toc19825)

[Figure S13. Age distribution of pancreatic cancer mortality attributable to HFPG by SDI quintiles, 1990-2019. 12](#_Toc14225)

[Figure S14. Age, period and cohort effects on tracheal, bronchus, and lung cancer mortality attributable to HFPG by SDI quintiles 13](#_Toc5637)

[Figure S15. Age, period and cohort effects on colon and rectal cancer mortality attributable to HFPG by SDI quintiles 14](#_Toc6361)

[Figure S16. Age, period and cohort effects on breast cancer mortality attributable to HFPG by SDI quintiles 15](#_Toc6078)

[Figure S17. Age, period and cohort effects on pancreatic cancer mortality attributable to HFPG by SDI quintiles 16](#_Toc7965)

[Figure S18. Age, period and cohort effects on tracheal, bronchus, and lung cancer mortality attributable to HFPG for representative countries. 17](#_Toc28233)

[Figure S19. Age, period and cohort effects on colon and rectal cancer mortality attributable to HFPG for representative countries. 18](#_Toc15520)

[Figure S20. Age, period and cohort effects on breast cancer mortality attributable to HFPG for representative countries. 19](#_Toc6907)

[Figure S21. Age, period and cohort effects on pancreatic cancer mortality attributable to HFPG for representative countries. 20](#_Toc27894)

[Table S1. Percent changes (%) in the age-standardized mortality rate 1990-2019 for all risk factors 21](#_Toc5195)

[Table S2. Trends in cancer mortality attributable to HPFG for females and males across SDI quintiles, 1990−2019. 22](#_Toc22831)

[Table S3. Trends in tracheal, bronchus, and lung cancer mortality attributable to HPFG for both females and males across SDI quintiles, 1990−2019. 26](#_Toc25039)

[Table S4. Trends in colon and rectal cancer mortality attributable to HPFG for both females and males across SDI quintiles, 1990−2019. 29](#_Toc27193)

[Table S5. Trends in breast cancer mortality attributable to HPFG for female across SDI quintiles, 1990−2019. 32](#_Toc12683)

[Table S6. Trends in pancreatic cancer mortality attributable to HPFG for both females and males across SDI quintiles, 1990−2019. 33](#_Toc9113)

[Table S7. Trends in cancer mortality attributable to HPFG for both genders in 204 countries and regions, 1990-2019. 37](#_Toc30802)

[Table S8. Trends in cancer mortality attributable to HPFG for both females and males in representative countries, 1990-2019. 54](#_Toc12459)

[Table S9. Trends in tracheal, bronchus, and lung cancer mortality attributable to HPFG for both females and males in representative countries, 1990-2019. 56](#_Toc32387)

[Table S10. Trends in colon and rectal cancer mortality attributable to HPFG for both females and males in representative countries, 1990-2019. 58](#_Toc8448)

[Table S11. Trends in breast cancer mortality attributable to HPFG for both females in representative countries, 1990-2019. 60](#_Toc881)

[Table S12. Trends in pancreatic cancer mortality attributable to HPFG for both females and males in representative countries, 1990-2019. 62](#_Toc29846)

# Figure S1. Rapid causes of cancer mortality worldwide for females and males, 1990-2019


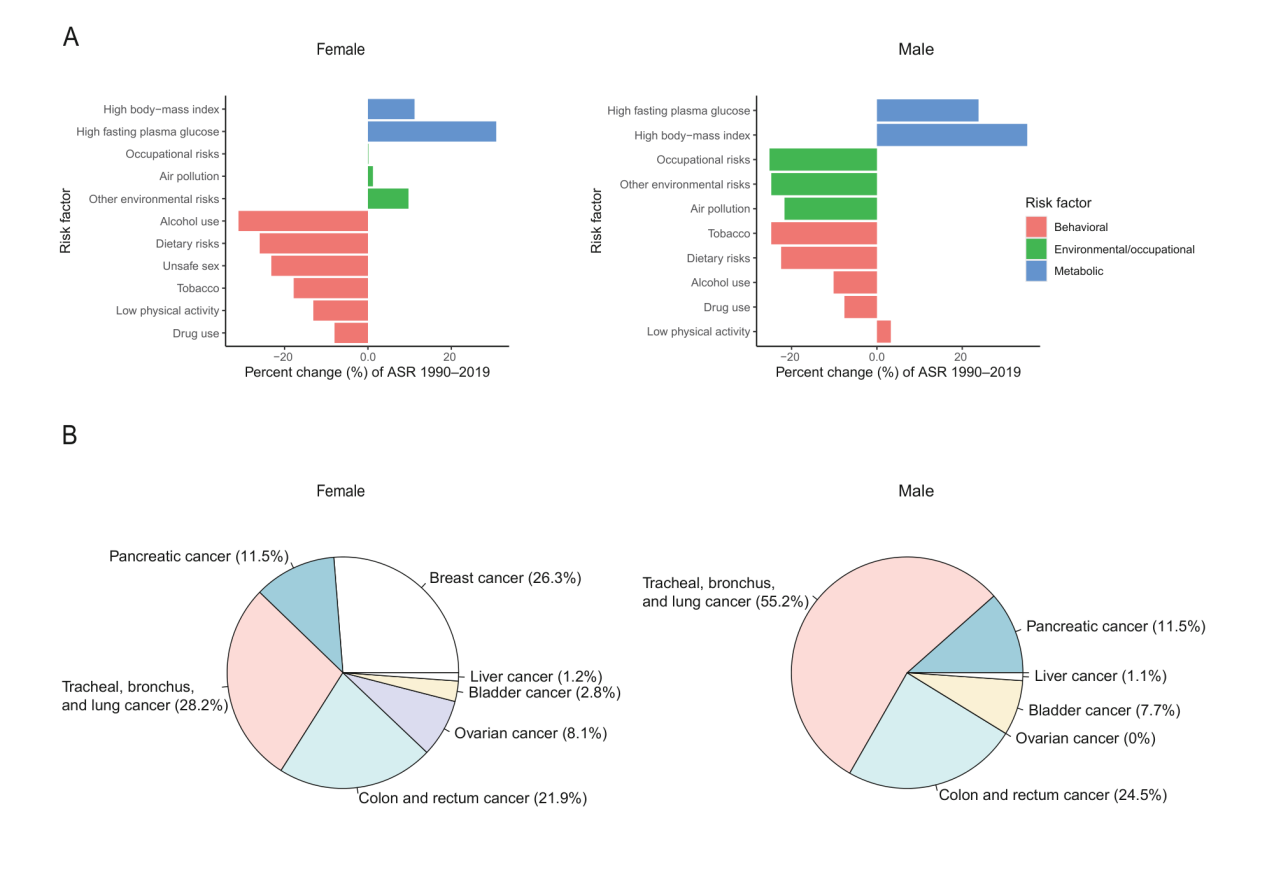


# Figure S2. Tracheal, bronchus, and lung cancer age-standardized mortality rates in 2019 (A) and percent change (%) in age-standardized rates during 1990-2019 (B) for 204 countries and territories by sociodemographic index.


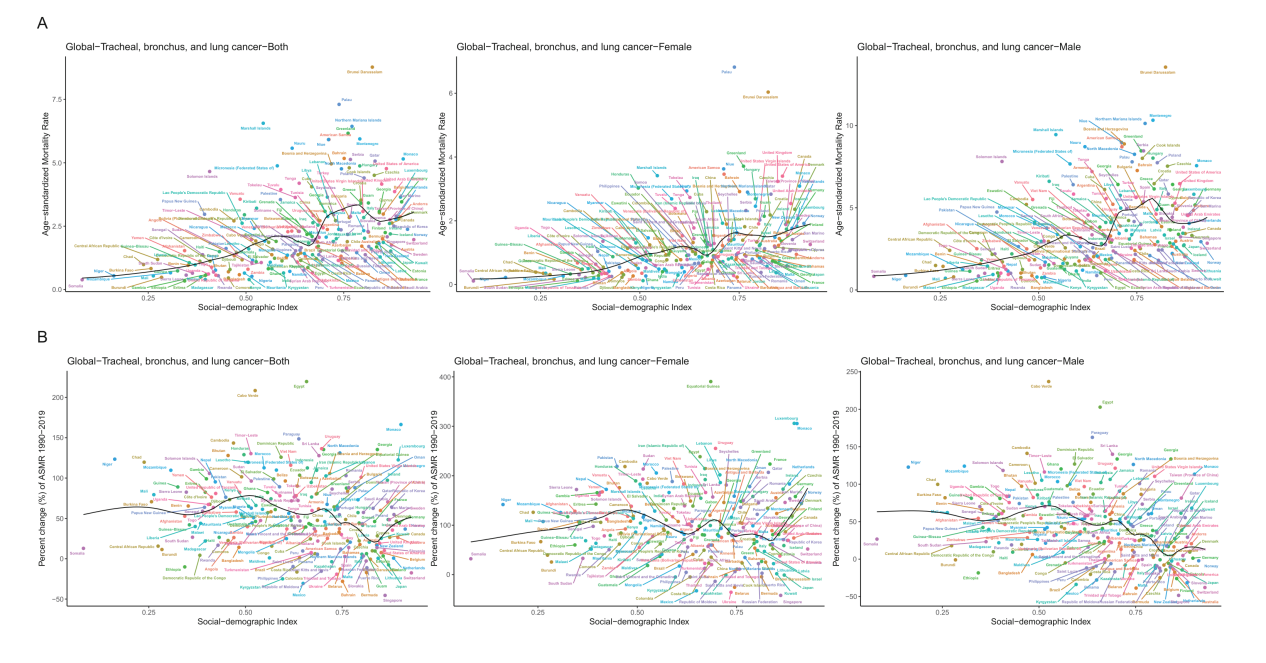


# Figure S3. The colon and rectal cancer age-standardized mortality rates in 2019 (A) and percent change (%) in the age-standardized rate during 1990-2019 (B) for 204 countries and territories by sociodemographic index.

**
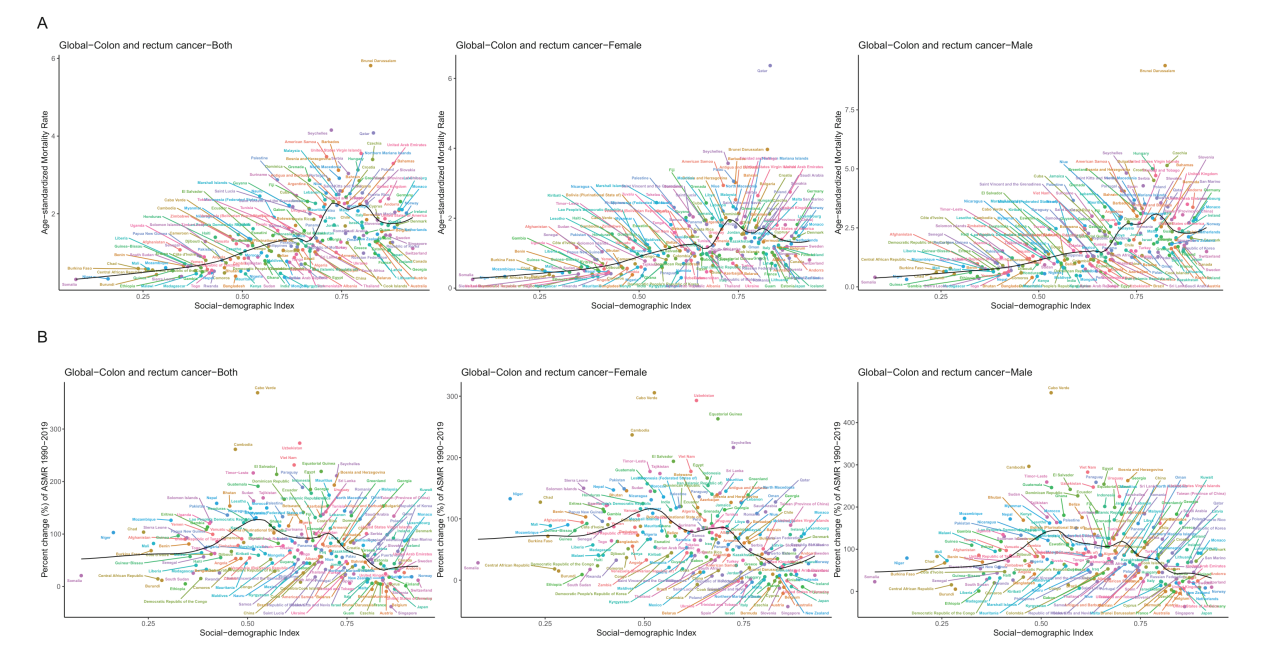
**

# Figure S4. Breast cancer age-standardized mortality rates in 2019 (A) and percent change (%) in age-standardized rates during 1990-2019 (B) for 204 countries and territories by sociodemographic index.

**
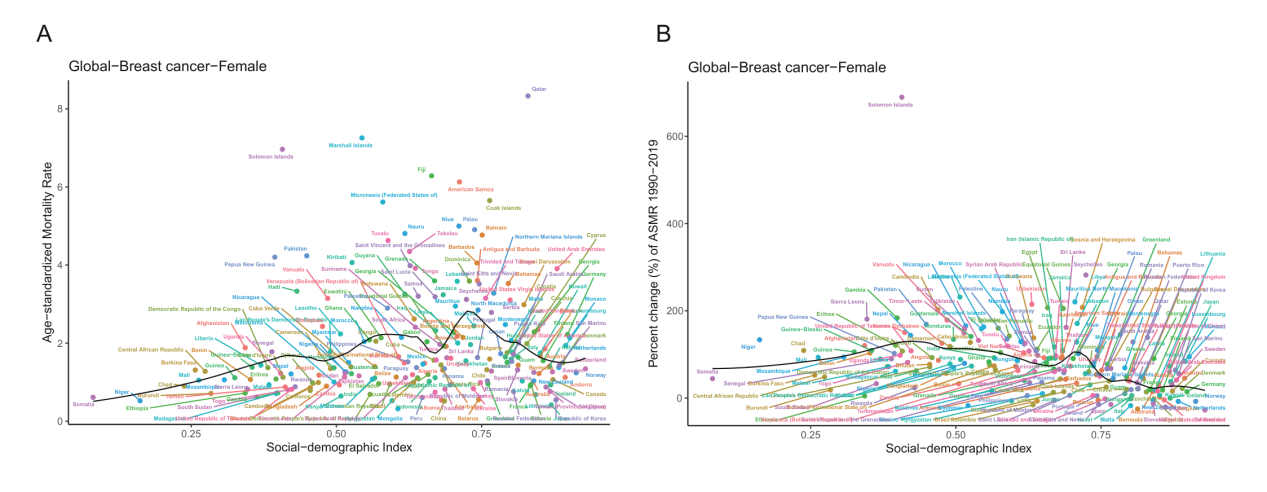
**

# Figure S5. Pancreatic cancer age-standardized mortality rates in 2019 (A) and percent change (%) in age-standardized rates during 1990-2019 (B) for 204 countries and territories by sociodemographic index.

**
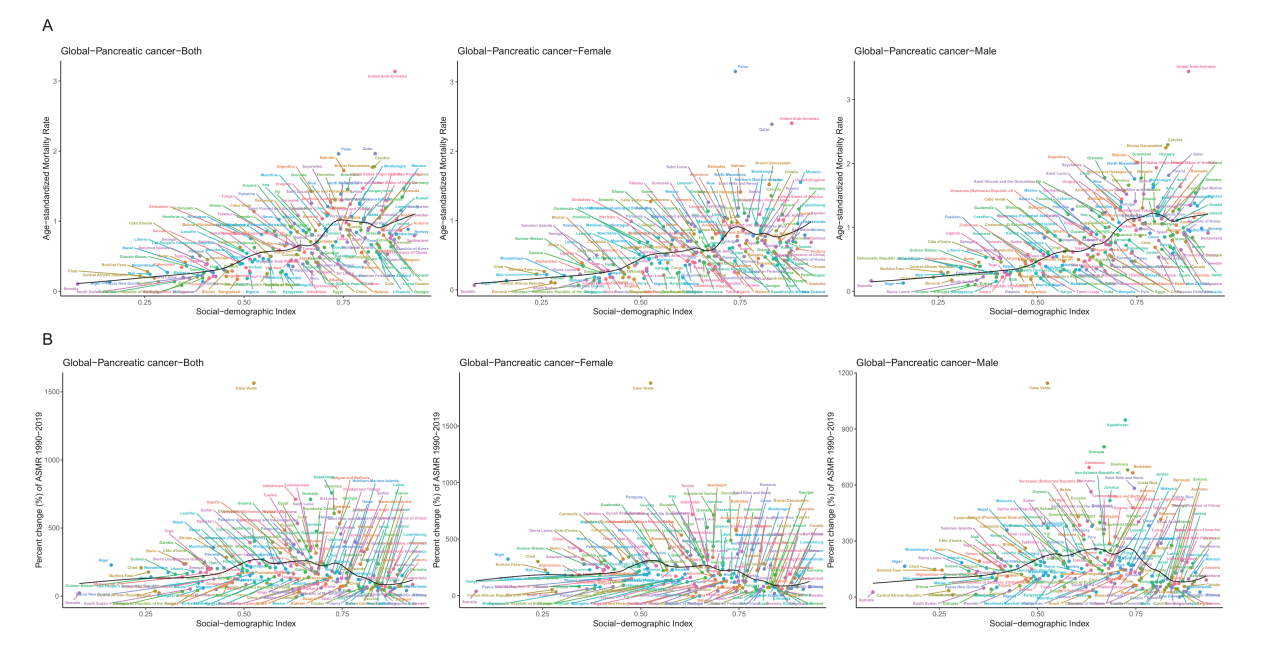
**

# Figure S6. Local drifts of tracheal, bronchus, and lung cancer mortality attributable to HFPG by SDI quintiles, 1990-2019.


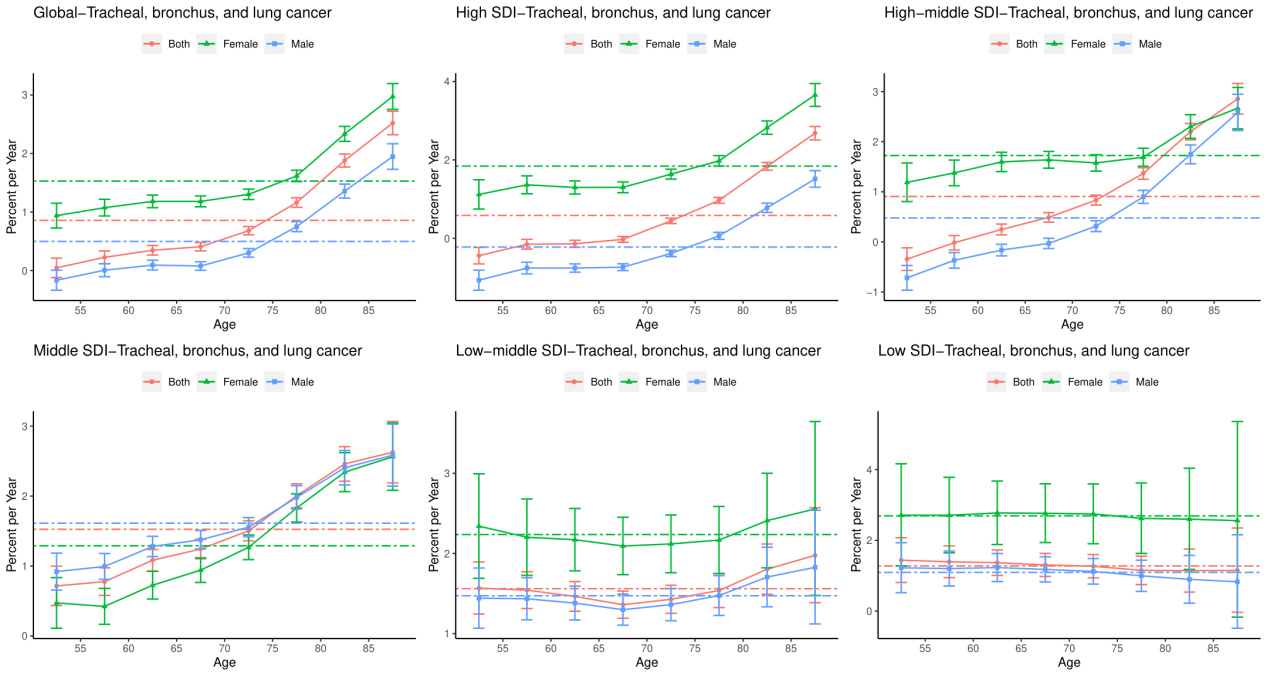


# Figure S7. Local drifts of colon and rectal cancer mortality attributable to HFPG by SDI quintiles, 1990-2019.


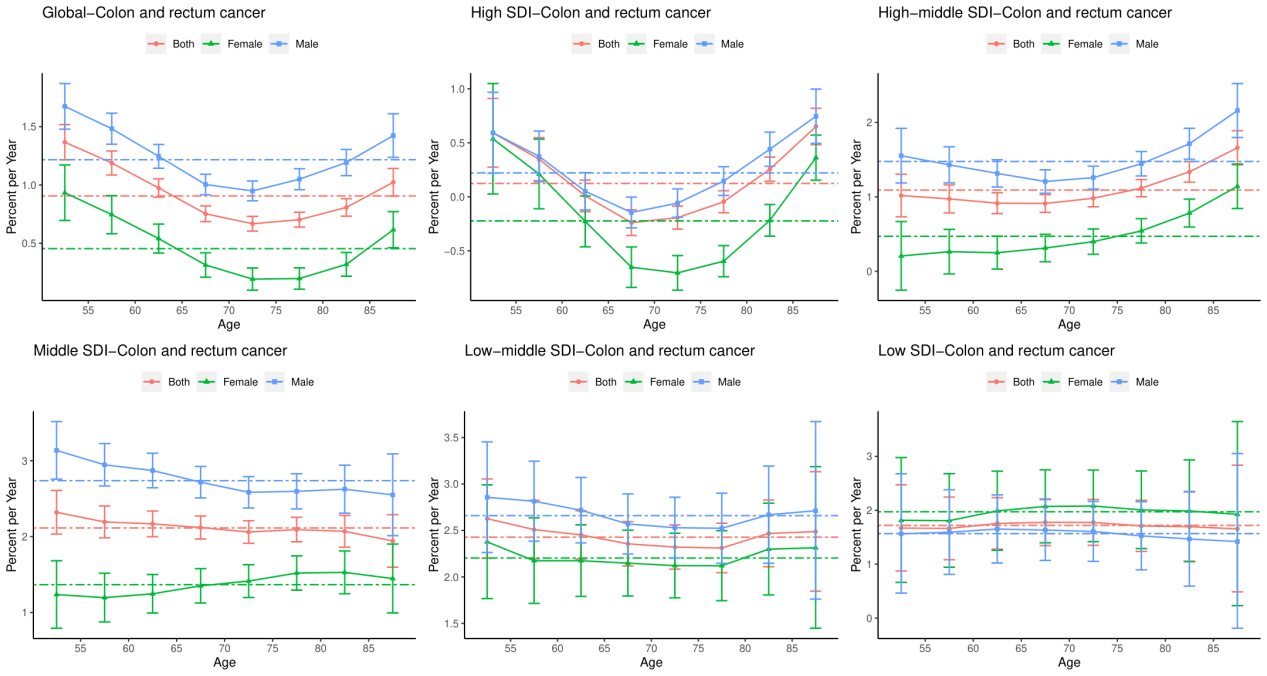


# Figure S8. Local drifts of breast cancer mortality attributable to HFPG by SDI quintiles, 1990-2019.


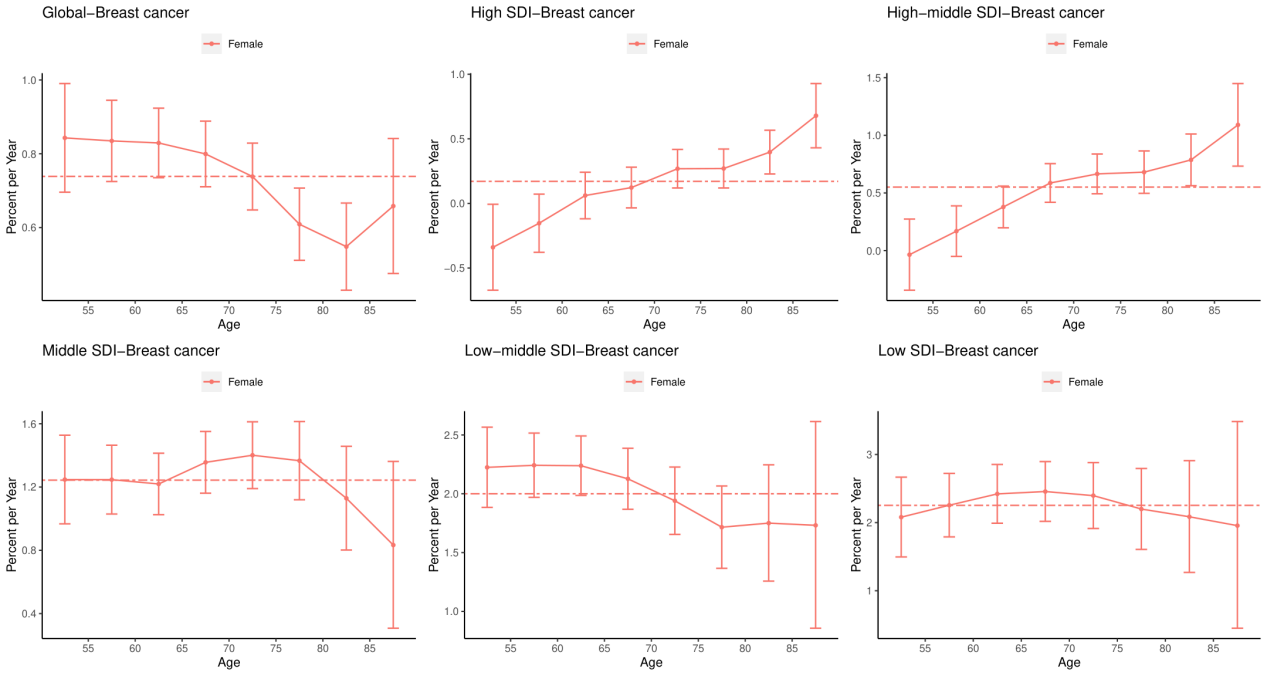


# Figure S9. Local drifts of pancreatic cancer mortality attributable to HFPG by SDI quintiles, 1990-2019.


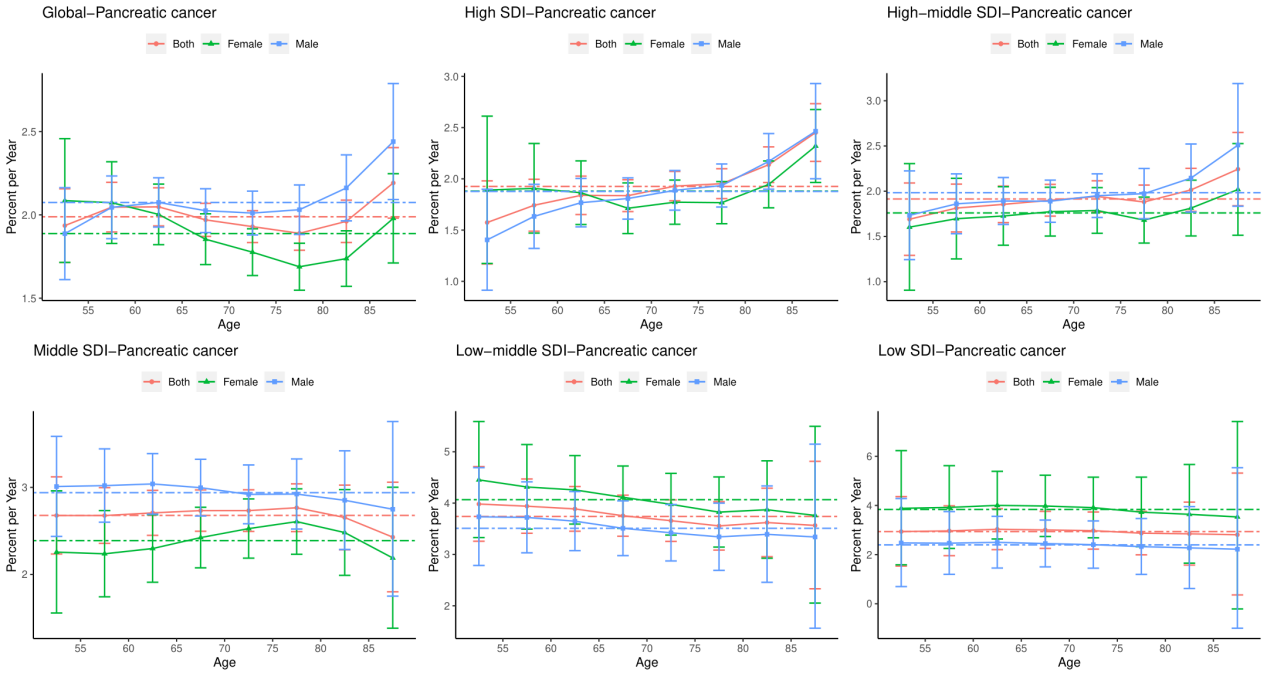


# Figure S10. Age distribution of tracheal, bronchus, and lung cancer mortality attributable to HFPG by SDI quintiles, 1990-2019.


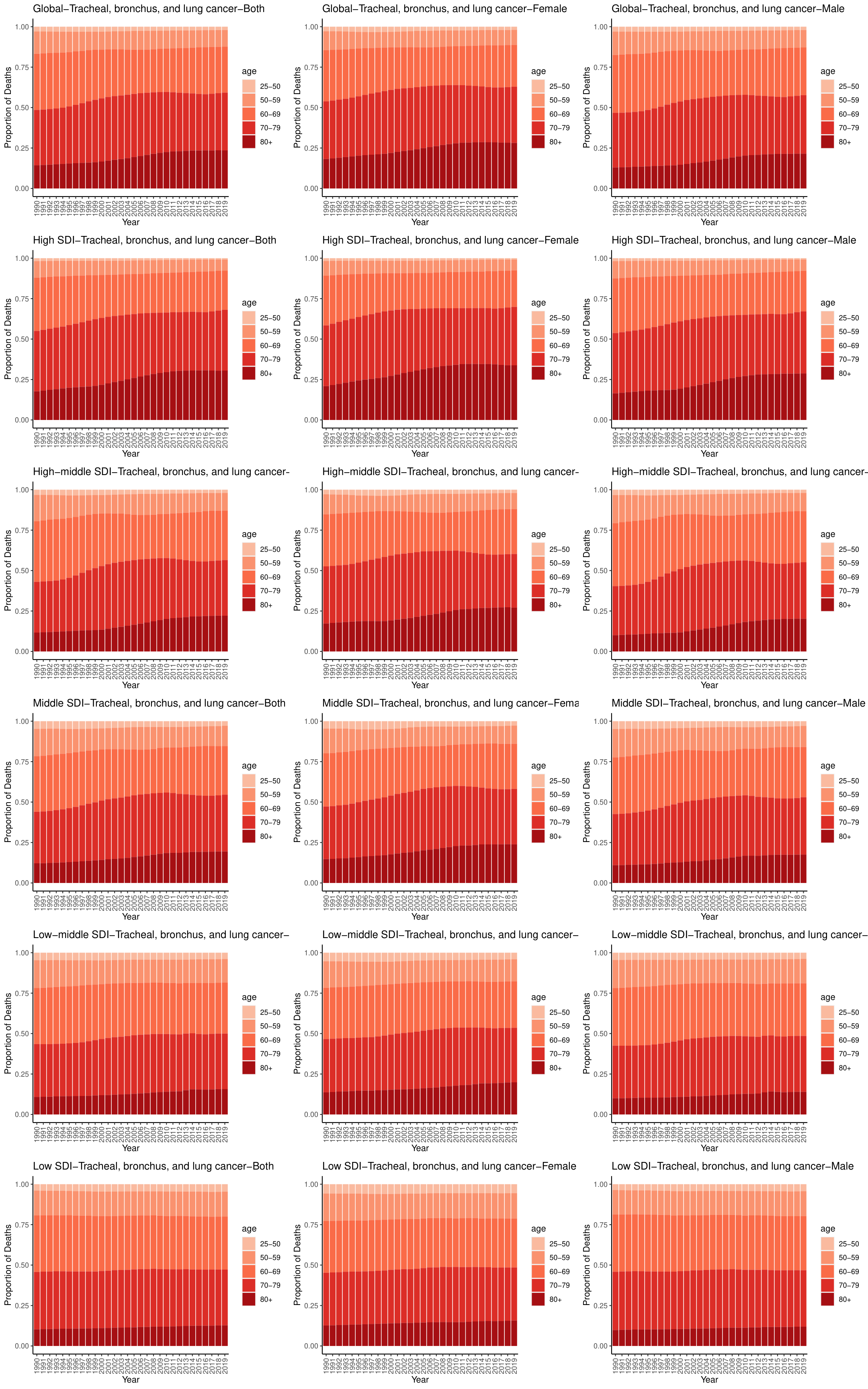


# Figure S11. Age distribution of colon and rectal cancer mortality attributable to HFPG by SDI quintiles, 1990-2019.


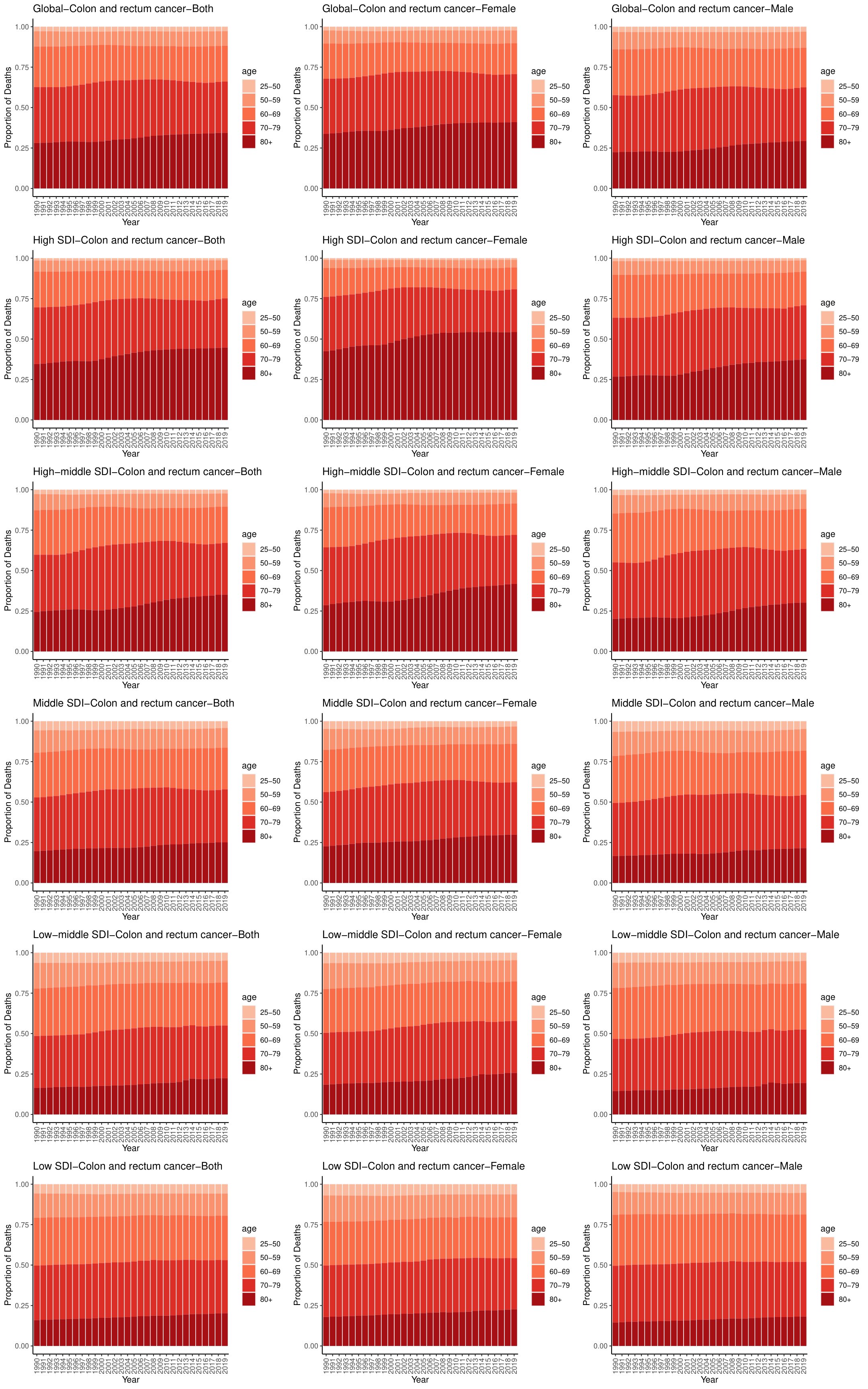


# Figure S12. Age distribution of breast cancer mortality attributable to HFPG by SDI quintiles, 1990-2019.


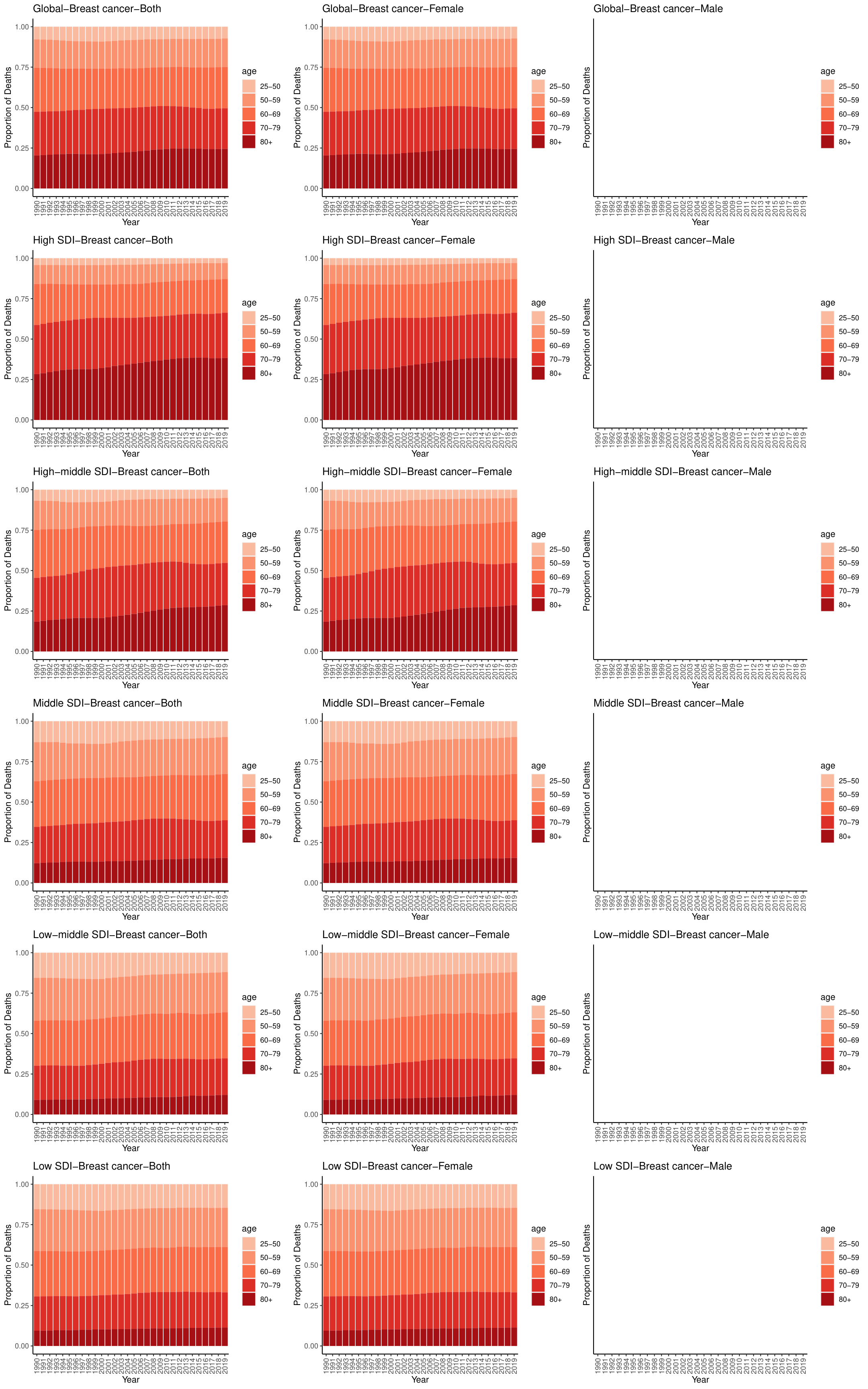


# Figure S13. Age distribution of pancreatic cancer mortality attributable to HFPG by SDI quintiles, 1990-2019.


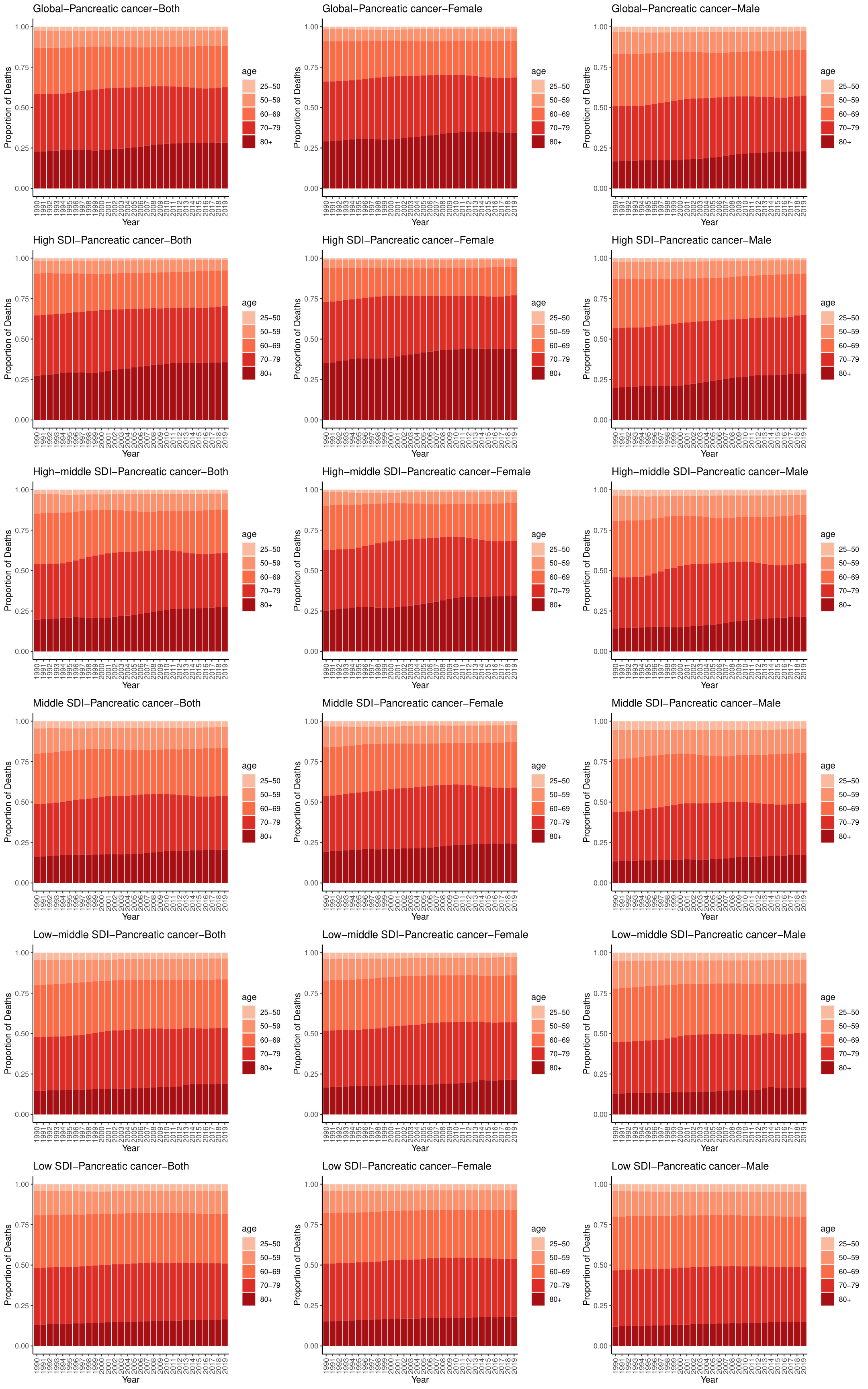


# Figure S14. Age, period and cohort effects on tracheal, bronchus, and lung cancer mortality attributable to HFPG by SDI quintiles


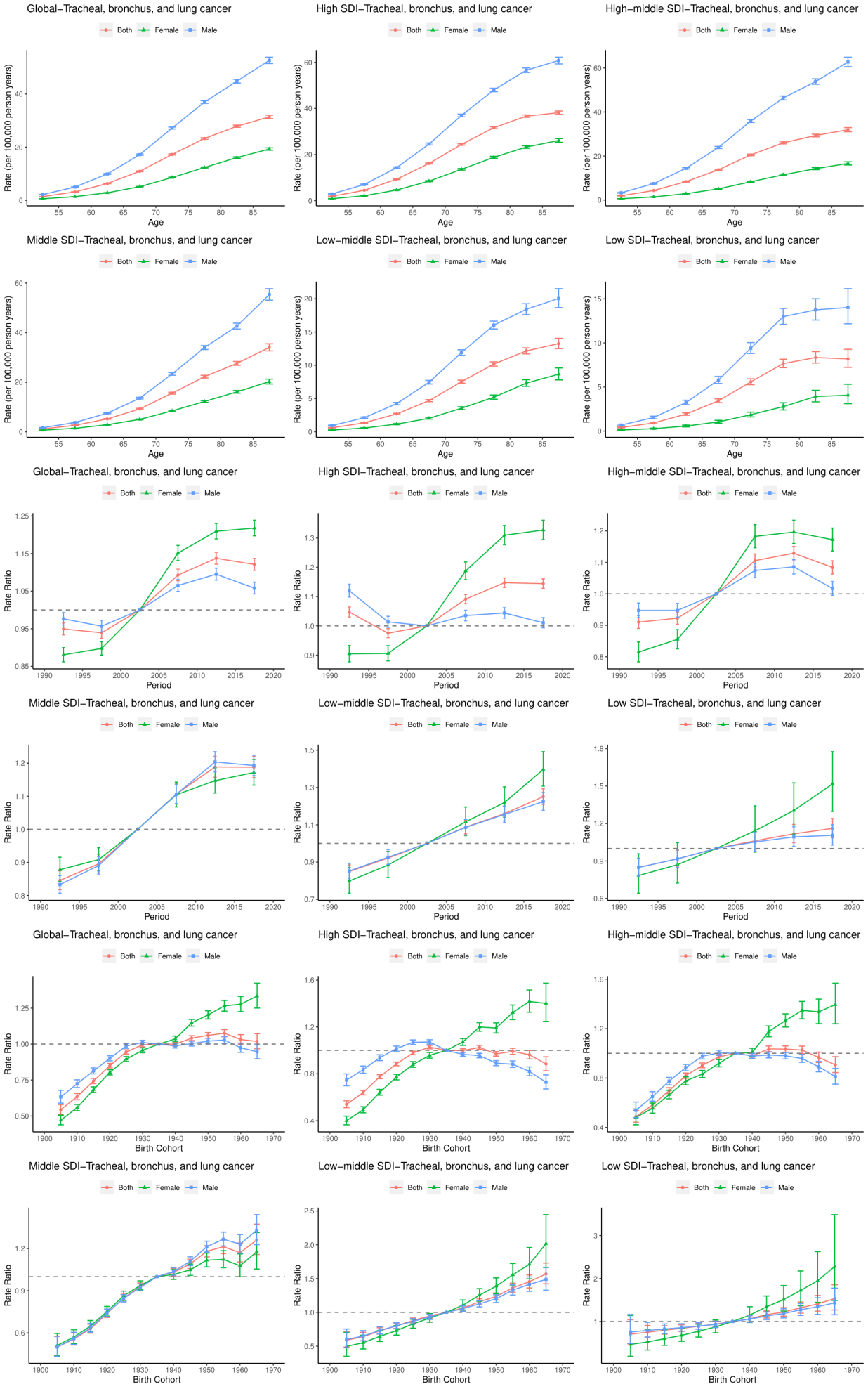


# Figure S15. Age, period and cohort effects on colon and rectal cancer mortality attributable to HFPG by SDI quintiles


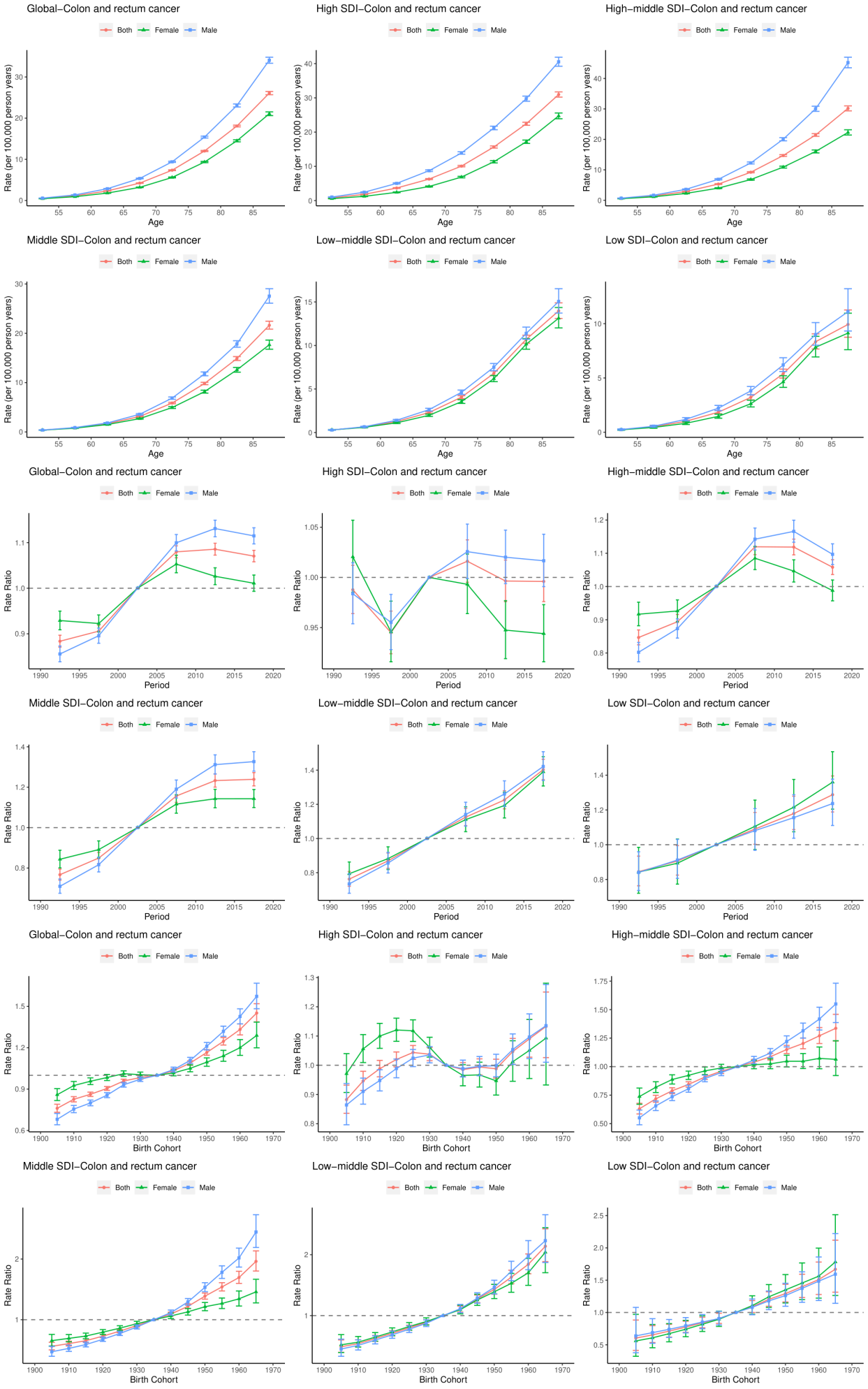


# Figure S16. Age, period and cohort effects on breast cancer mortality attributable to HFPG by SDI quintiles


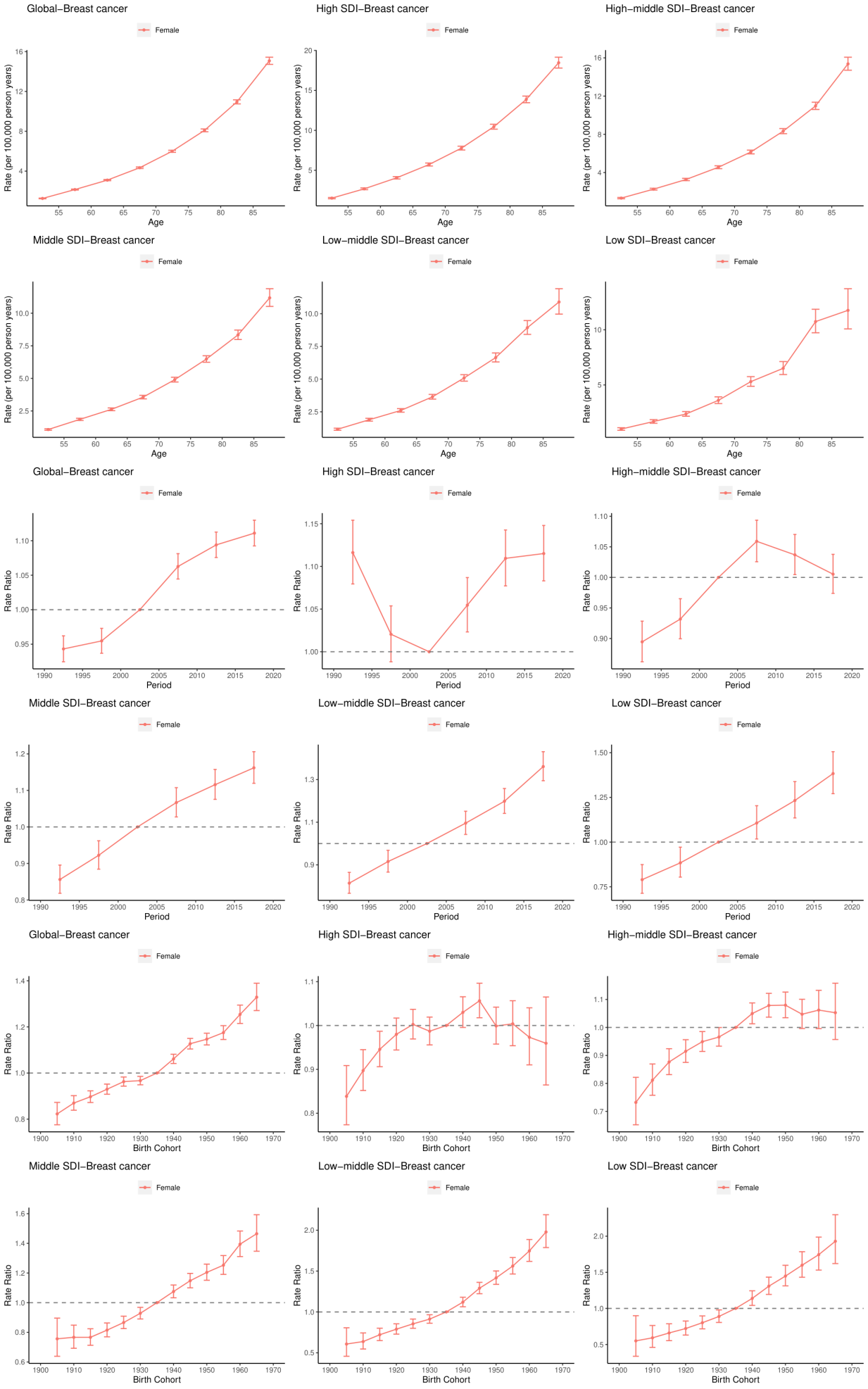


# Figure S17. Age, period and cohort effects on pancreatic cancer mortality attributable to HFPG by SDI quintiles

**
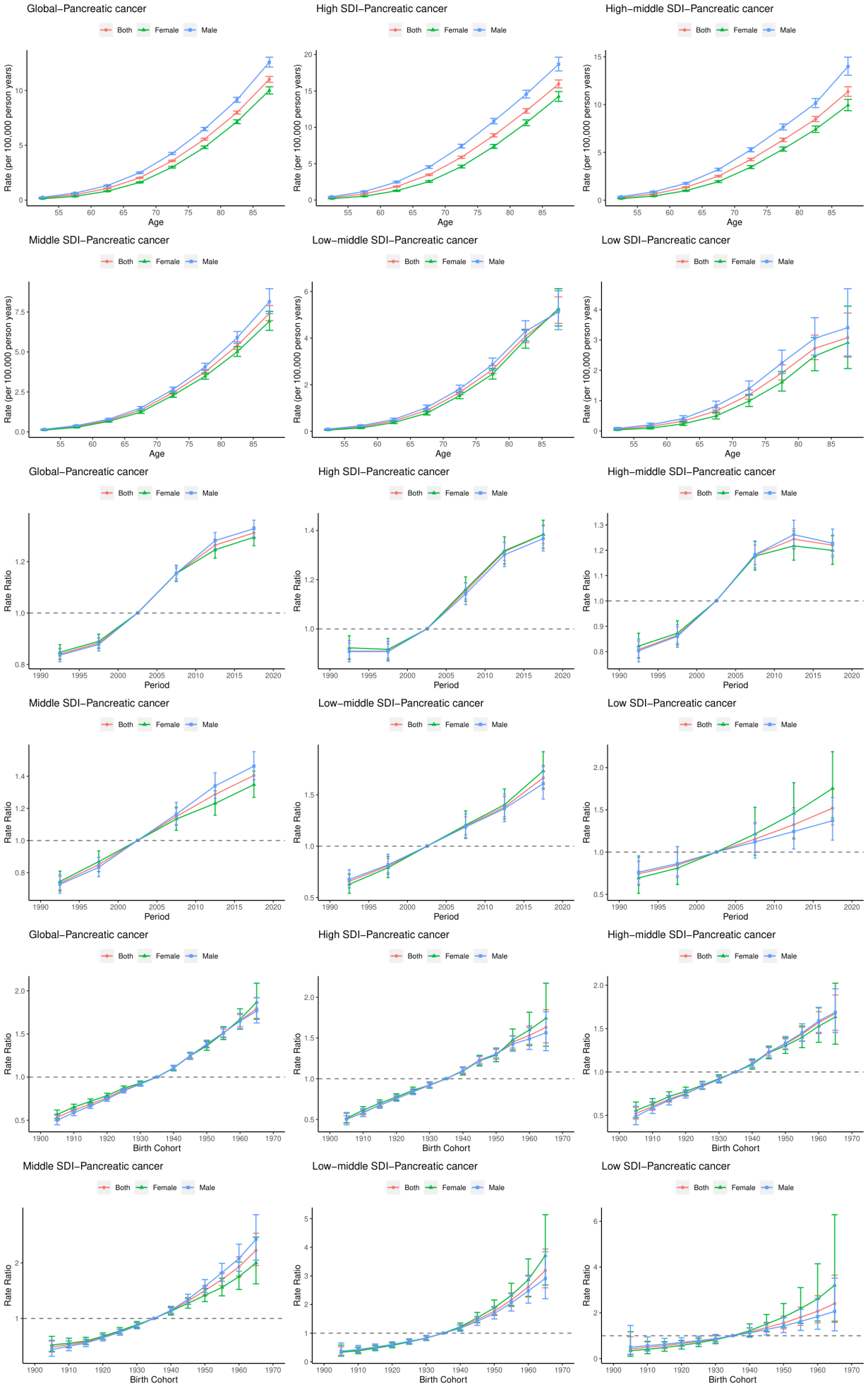
**

# Figure S18. Age, period and cohort effects on tracheal, bronchus, and lung cancer mortality attributable to HFPG for representative countries.


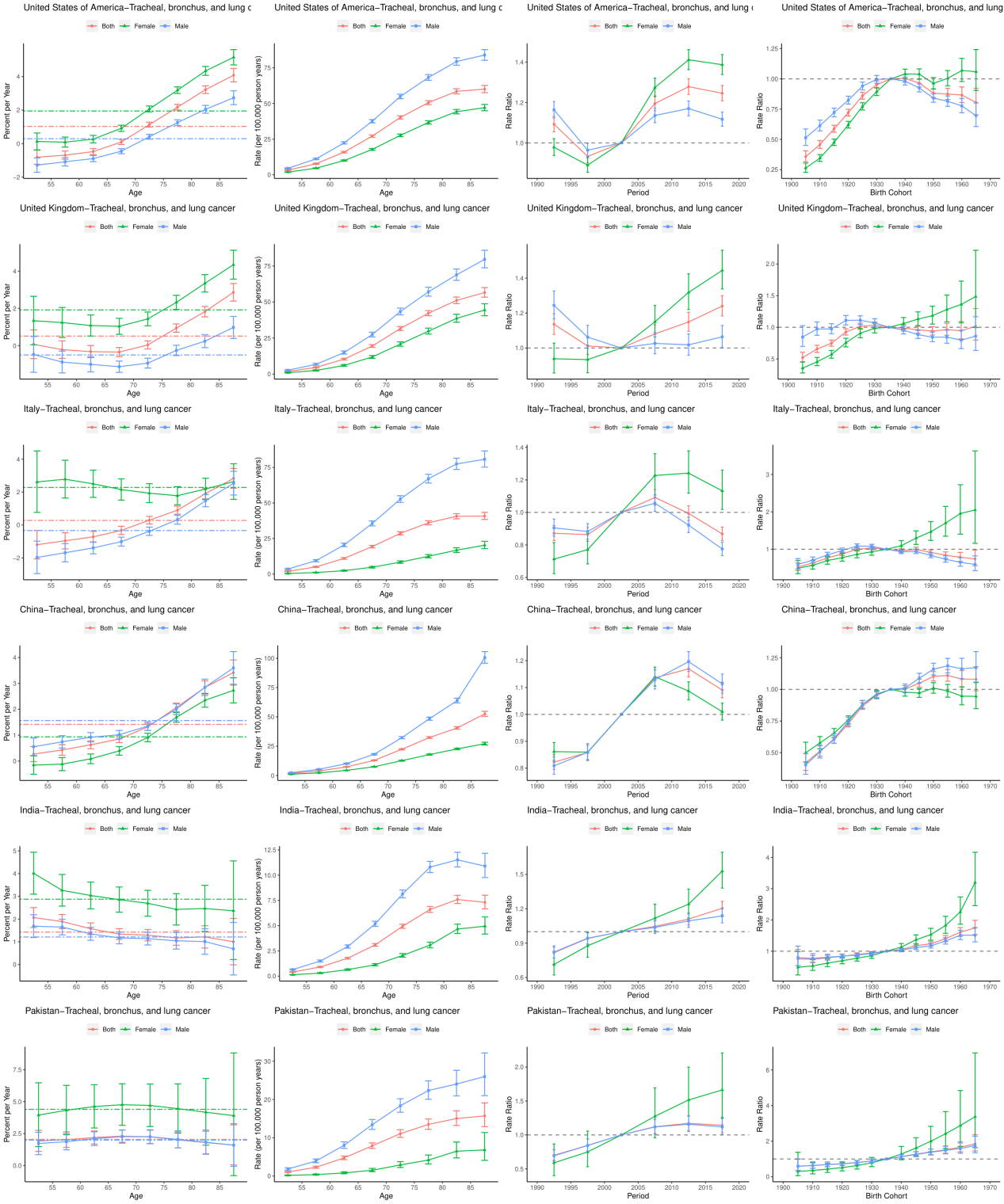


# Figure S19. Age, period and cohort effects on colon and rectal cancer mortality attributable to HFPG for representative countries.


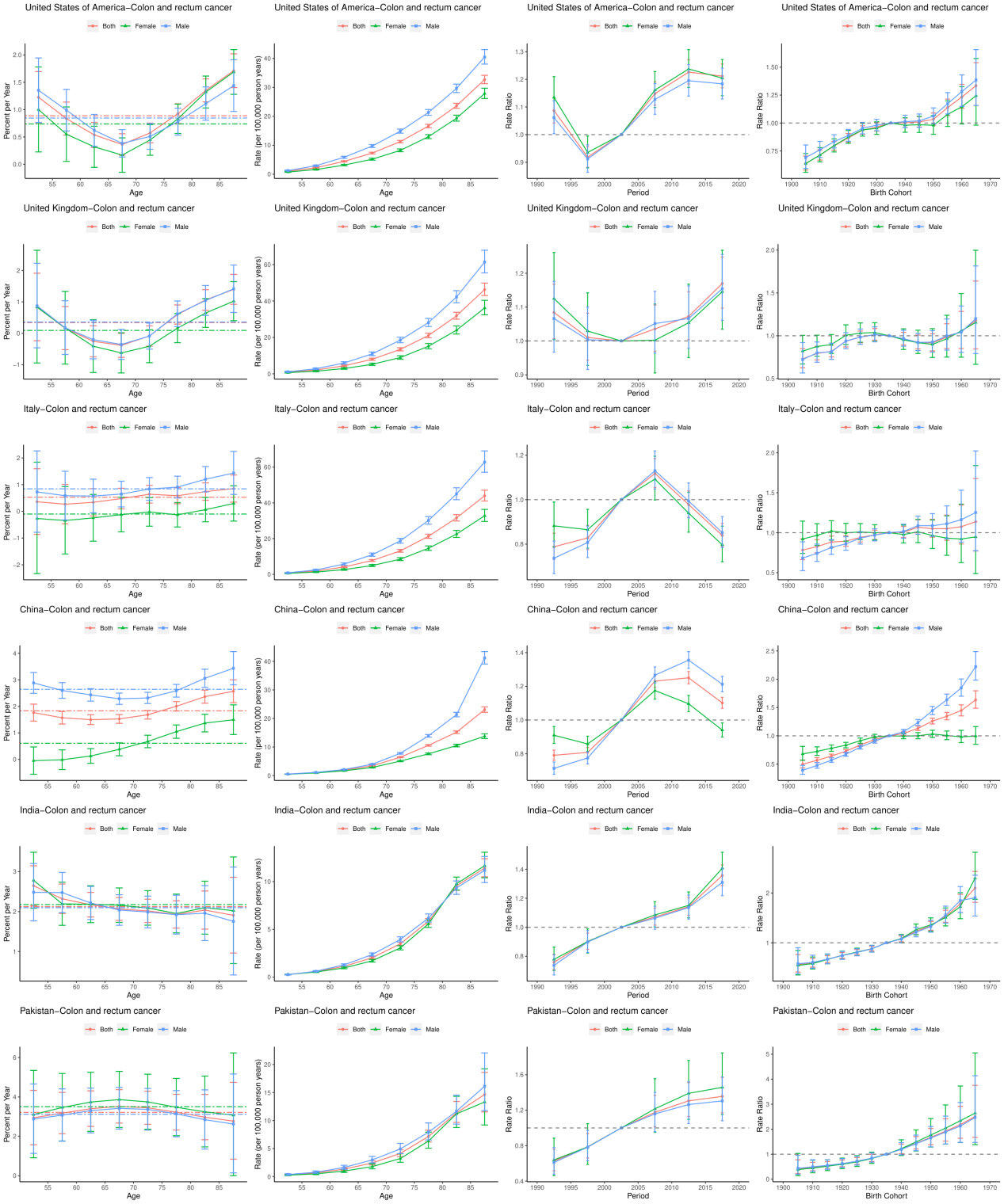


# Figure S20. Age, period and cohort effects on breast cancer mortality attributable to HFPG for representative countries.


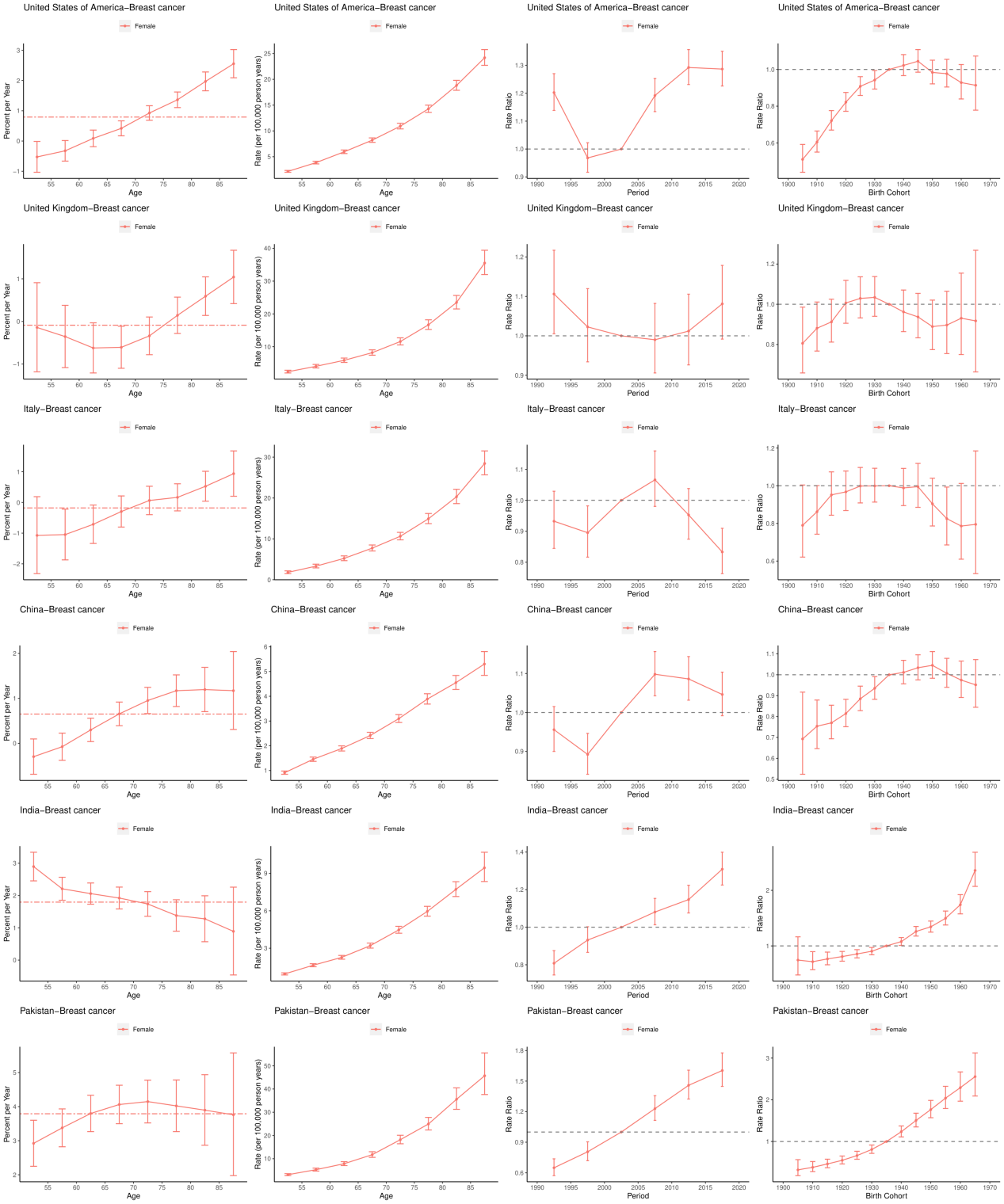


# Figure S21. Age, period and cohort effects on pancreatic cancer mortality attributable to HFPG for representative countries.


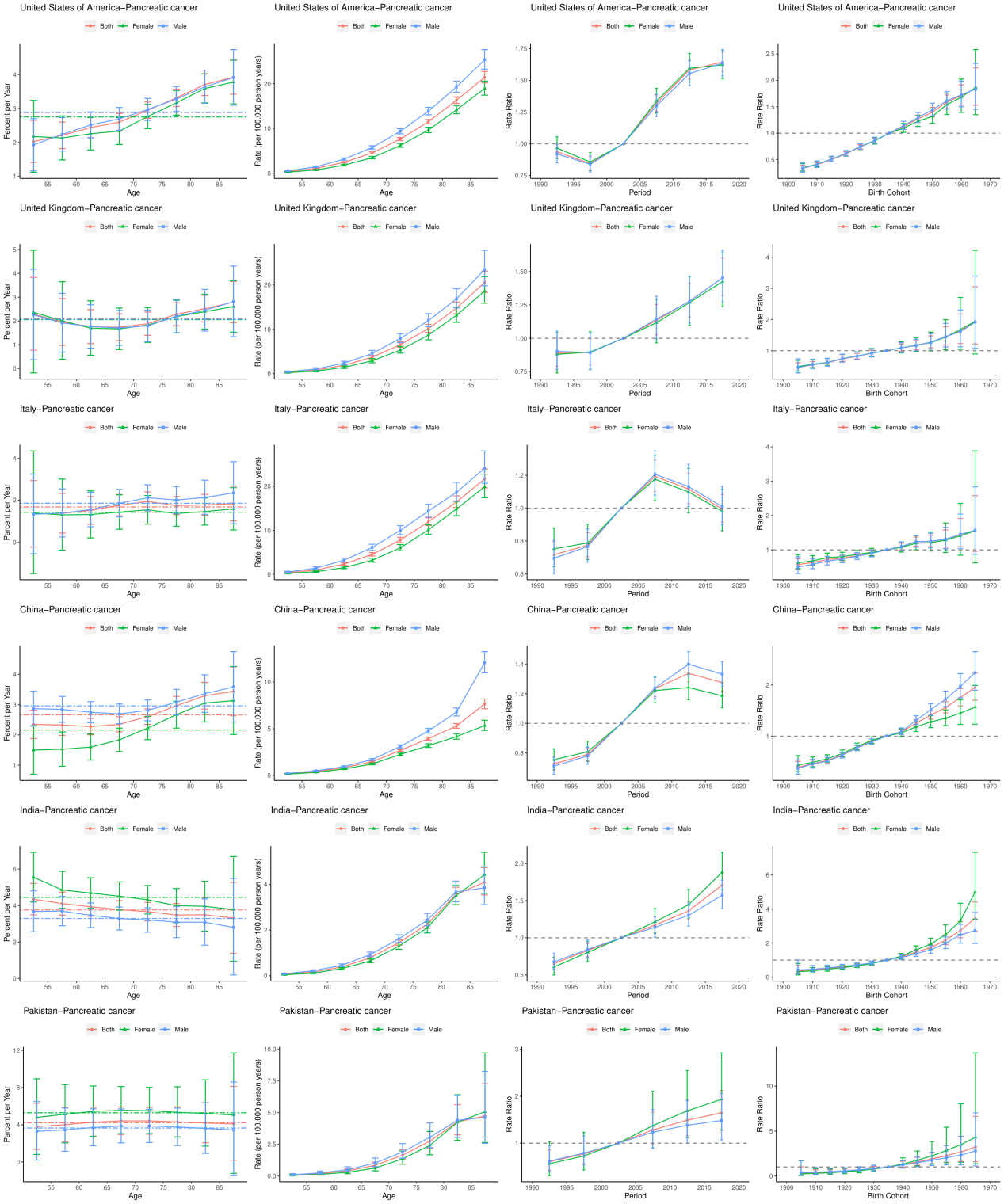


# Table S1. Percent changes (%) in the age-standardized mortality rate 1990-2019 for all risk factors

| **Both** | |  | **Female** | |  | **Female** | |
| --- | --- | --- | --- | --- | --- | --- | --- |
| **Risk factor** | **Percent change** |  | **Risk factor** | **Percent change** |  | **Risk factor** | **Percent change** |
| High fasting plasma glucose | 27.8% (38.7%,20.5%) |  | High fasting plasma glucose | 30.8% (42.2%,22.3%) |  | High body-mass index | 35.2% (70.9%,14.1%) |
| High body-mass index | 21.7% (42.9%,7%) |  | High body-mass index | 11.2% (27.7%, -1%) |  | High fasting plasma glucose | 23.8% (38%,14.1%) |
| Low physical activity | -7.7% (0.3%, -14%) |  | Other environmental risks | 9.7% (21.9%, -1.1%) |  | Low physical activity | 3.3% (19.9%, -6.5%) |
| Drug use | -8% (9.6%, -22.8%) |  | Air pollution | 1.2% (23.1%, -17.3%) |  | Drug use | -7.6% (10.8%, -23.2%) |
| Alcohol use | -14.7% (-6.4%, -23%) |  | Occupational risks | 0.1% (16.8%, -16.9%) |  | Alcohol use | -10.2% (0.5%, -20.5%) |
| Air pollution | -14.8% (1.2%, -29.6%) |  | Drug use | -8% (19.3%, -28.7%) |  | Air pollution | -21.7% (-4.3%, -36.8%) |
| Other environmental risks | -15.3% (-5.9%, -23.5%) |  | Low physical activity | -13.1% (-4.4%, -19.5%) |  | Dietary risks | -22.5% (-10.2%, -32.9%) |
| Occupational risks | -19.4% (-13.5%, -25.3%) |  | Tobacco | -17.8% (-11.8%, -23.1%) |  | Other environmental risks | -24.8% (-14.4%, -33%) |
| Tobacco | -22.1% (-0.151, -28.5%) |  | Unsafe sex | -23.2% (-12.3%, -34.7%) |  | Tobacco | -24.8% (-16.3%, -32.1%) |
| Dietary risks | -23.6% (-14.2%, -32.7%) |  | Dietary risks | -26% (-17.1%, -34.4%) |  | Occupational risks | -25.2% (-19.5%, -30.5%) |
| Unsafe sex | -23.9% (-13.4%, -35.2%) |  | Alcohol use | -31.1% (-26%, -35.9%) |  |  |  |

# Table S2. Trends in cancer mortality attributable to HPFG for females and males across SDI quintiles, 1990−2019.

|  | **Global** | | **High SDI** | | **High-middle SDI** | | **Middle SDI** | | **Low-middle SDI** | | **Low SDI** | |
| --- | --- | --- | --- | --- | --- | --- | --- | --- | --- | --- | --- | --- |
|  | **1990** | **2019** | **1990** | **2019** | **1990** | **2019** | **1990** | **2019** | **1990** | **2019** | **1990** | **2019** |
| **Female** | | | | | | | | | | | | |
| Population |  |  |  |  |  |  |  |  |  |  |  |  |
| Number, n ×1,000,000 | 2656 (2602,2710) | 3856 (3732,3982) | 417 | 508 | 578 | 716 | 844 | 1191 | 553 | 878 | 262 | 563 |
| Percentage of global, % | 100 | 100 | 15.71 | 13.17 | 21.78 | 18.57 | 31.77 | 30.88 | 20.84 | 22.76 | 9.85 | 14.59 |
| Deaths |  |  |  |  |  |  |  |  |  |  |  |  |
| Number*, n× 1,000 | 69.12(18.17,143.11) | 194.51(53.41,409.51) | 31.99(8.47,66.57) | 68.78(18.88,140.01) | 19.2(4.99,40.26) | 48.85(13.14,104.28) | 11.95(3.14,25.2) | 47.23(12.6,99.9) | 4.52(1.13,9.77) | 23.06(6.14,49.16) | 1.41(0.34,3.12) | 6.46(1.6,14.02) |
| Percentage of global, % | 100 | 100 | 46.28 | 35.36 | 27.78 | 25.12 | 17.29 | 24.28 | 6.55 | 11.86 | 2.04 | 3.32 |
| Percent change of deaths  1990–2019, % | 181.4(161.98,206.11) |  | 114.99(100.38,137.8) |  | 154.42(134.17,182.74) |  | 295.11(248.22,352.18) |  | 409.68(332.93,520.32) |  | 357.83(282.71,473.94) |  |
| All-age mortality rate |  |  |  |  |  |  |  |  |  |  |  |  |
| Rate per 100,000 | 2.6(0.68,5.39) | 5.04(1.38,10.62) | 7.67(2.03,15.96) | 13.55(3.72,27.58) | 3.32(0.86,6.96) | 6.82(1.84,14.57) | 1.42(0.37,2.99) | 3.97(1.06,8.39) | 0.82(0.2,1.76) | 2.63(0.7,5.6) | 0.54(0.13,1.19) | 1.15(0.29,2.49) |
| Percent change of rate 1990–2019, % | 93.81(80.44,110.83) |  | 76.67(64.65,95.41) |  | 105.55(89.19,128.43) |  | 180.04(146.81,220.49) |  | 221.47(173.05,291.25) |  | 113.03(78.08,167.06) |  |
| Age-standardized mortality rate |  |  |  |  |  |  |  |  |  |  |  |  |
| Rate per 100,000 | 3.39(0.88,7.03) | 4.43(1.22,9.33) | 5.04(1.34,10.48) | 6.06(1.65,12.38) | 3.23(0.84,6.78) | 4.21(1.13,9.02) | 2.49(0.66,5.25) | 3.8(1.02,8.02) | 1.7(0.43,3.63) | 3.41(0.91,7.24) | 1.38(0.33,3.07) | 2.69(0.68,5.82) |
| Percent change of rate 1990–2019, % | 30.77(22.25,42.23) |  | 20.23(12.78,32.95) |  | 30.54(20.4,44.69) |  | 52.62(34.6,73.81) |  | 100.37(69.44,143.88) |  | 95.53(63.63,146.73) |  |
| APC model estimates |  |  |  |  |  |  |  |  |  |  |  |  |
| Net drift of mortality y, % per year | 1(0.93,1.07) |  | 0.8(0.73,0.86) |  | 0.96(0.89,1.02) |  | 1.36(1.25,1.48) |  | 2.31(2.19,2.43) |  | 2.33(2.12,2.55) |  |
| **Male** | | | | | | | | | | | | |
| Population |  |  |  |  |  |  |  |  |  |  |  |  |
| Number, n ×1,000,000 | 2694 (2637,2750) | 3881 (3751,4010) | 405 | 506 | 572 | 715 | 873 | 1206 | 576 | 886 | 266 | 566 |
| Percentage of global, % | 100 | 100 | 15.03 | 13.03 | 21.23 | 18.41 | 32.40 | 31.07 | 21.39 | 22.84 | 9.89 | 14.59 |
| Deaths |  |  |  |  |  |  |  |  |  |  |  |  |
| Number*, n× 1,000 | 80.98(18.93,175.68) | 224.83(55.52,481.52) | 35.84(8.41,77.47) | 77.03(19.75,159.77) | 25.22(5.76,55.59) | 65.12(15.56,140.57) | 13.33(2.98,29.82) | 56.92(13.48,125.4) | 4.82(1.08,10.89) | 20.1(4.87,43.04) | 1.72(0.39,3.9) | 5.52(1.38,12.09) |
| Percentage of global, % | 100 | 100 | 44.26 | 34.26 | 31.14 | 28.96 | 16.46 | 25.32 | 5.96 | 8.94 | 2.13 | 2.45 |
| Percent change of deaths  1990–2019, % | 177.64(154.41,213.86) |  | 114.93(101.87,142.08) |  | 158.22(131.83,198.47) |  | 327.06(258.47,415.4) |  | 316.9(260.49,411.83) |  | 220.61(179.35,289.16) |  |
| All-age mortality rate |  |  |  |  |  |  |  |  |  |  |  |  |
| Rate per 100,000 | 3.01(0.7,6.52) | 5.79(1.43,12.41) | 8.85(2.08,19.14) | 15.23(3.91,31.59) | 4.41(1.01,9.72) | 9.11(2.18,19.67) | 1.53(0.34,3.42) | 4.72(1.12,10.4) | 0.84(0.19,1.89) | 2.27(0.55,4.86) | 0.65(0.15,1.46) | 0.97(0.24,2.14) |
| Percent change of rate 1990–2019, % | 92.71(76.58,117.85) |  | 72.06(61.61,93.79) |  | 106.75(85.61,138.97) |  | 209.11(159.47,273.05) |  | 170.97(134.31,232.67) |  | 50.86(31.44,83.11) |  |
| Age-standardized mortality rate |  |  |  |  |  |  |  |  |  |  |  |  |
| Rate per 100,000 | 5.1(1.21,10.96) | 6.32(1.56,13.42) | 8.31(1.95,17.93) | 8.7(2.23,18.04) | 6.03(1.4,13.09) | 7.48(1.8,16.05) | 3.2(0.74,7.04) | 5.33(1.27,11.7) | 1.89(0.44,4.24) | 3.4(0.83,7.2) | 1.77(0.43,3.96) | 2.59(0.66,5.59) |
| Percent change of rate 1990–2019, % | 23.82(14.1,37.96) |  | 4.63(-1.45,16.9) |  | 24.15(12.36,41.58) |  | 66.26(41.67,97.9) |  | 79.57(56.1,118.89) |  | 46.11(28.84,76.98) |  |
| APC model estimates |  |  |  |  |  |  |  |  |  |  |  |  |
| Net drift of mortality y, % per year | 0.77(0.72,0.81) |  | 0.17(0.12,0.22) |  | 0.73(0.66,0.79) |  | 1.83(1.74,1.92) |  | 1.88(1.76,1.99) |  | 1.26(1.06,1.46) |  |

# Table S3. Trends in tracheal, bronchus, and lung cancer mortality attributable to HPFG for both females and males across SDI quintiles, 1990−2019.

|  | **Global** | | **High SDI** | | **High-middle SDI** | | **Middle SDI** | | **Low-middle SDI** | | **Low SDI** | |
| --- | --- | --- | --- | --- | --- | --- | --- | --- | --- | --- | --- | --- |
|  | **1990** | **2019** | **1990** | **2019** | **1990** | **2019** | **1990** | **2019** | **1990** | **2019** | **1990** | **2019** |
| **Both** | | | | | | | | | | | | |
| Deaths |  |  |  |  |  |  |  |  |  |  |  |  |
| Number*, n× 1,000 | 67.26(14.64,151.22) | 179.05(42.68,389.38) | 29.45(6.63,64.87) | 61.89(15.11,131.91) | 20.39(4.2,46.93) | 49.56(11.25,109.61) | 12.53(2.77,28.9) | 48.93(11.23,109.85) | 3.77(0.76,8.78) | 14.94(3.36,33.47) | 1.1(0.2,2.73) | 3.62(0.75,8.31) |
| Percentage of global, % | 100 | 100 | 43.78 | 34.57 | 30.31 | 27.68 | 18.62 | 27.33 | 5.6 | 8.34 | 1.63 | 2.02 |
| Percent change of deaths  1990–2019, % | 166.19(142.95,198.32) |  | 110.16(95.62,141.26) |  | 143.1(117.81,178.03) |  | 290.62(230.98,359.43) |  | 296.53(227.16,382.25) |  | 230.92(181.72,306.27) |  |
| All-age mortality rate |  |  |  |  |  |  |  |  |  |  |  |  |
| Rate per 100,000 | 1.26(0.27,2.83) | 2.31(0.55,5.03) | 3.58(0.81,7.89) | 6.11(1.49,13.02) | 1.77(0.37,4.08) | 3.46(0.79,7.66) | 0.73(0.16,1.68) | 2.04(0.47,4.58) | 0.33(0.07,0.78) | 0.85(0.19,1.9) | 0.21(0.04,0.52) | 0.32(0.07,0.74) |
| Percent change of rate 1990–2019, % | 84.05(67.98,106.27) |  | 70.47(58.68,95.7) |  | 95.52(75.18,123.61) |  | 179.81(137.09,229.11) |  | 153.93(109.51,208.83) |  | 54.84(31.83,90.11) |  |
| Age-standardized mortality rate |  |  |  |  |  |  |  |  |  |  |  |  |
| Rate per 100,000 | 1.78(0.39,3.99) | 2.22(0.53,4.83) | 2.76(0.62,6.07) | 3.11(0.76,6.6) | 1.92(0.4,4.4) | 2.41(0.55,5.32) | 1.35(0.3,3.08) | 2.1(0.48,4.68) | 0.71(0.15,1.65) | 1.17(0.27,2.61) | 0.54(0.1,1.33) | 0.79(0.17,1.81) |
| Percent change of rate 1990–2019, % | 25.19(14.52,39.63) |  | 12.91(5.36,28.87) |  | 25.05(12.31,41.78) |  | 54.93(32.05,80.22) |  | 64.38(36.13,98.87) |  | 48.01(26.5,80.45) |  |
| APC model estimates |  |  |  |  |  |  |  |  |  |  |  |  |
| Net drift of mortality y, % per year | 0.86(0.8,0.91) |  | 0.58(0.53,0.64) |  | 0.91(0.83,0.98) |  | 1.53(1.42,1.63) |  | 1.56(1.43,1.69) |  | 1.28(1.02,1.54) |  |
| **Female** | | | | | | | | | | | | |
| Deaths |  |  |  |  |  |  |  |  |  |  |  |  |
| Number*, n× 1,000 | 17.28(3.23,41.14) | 54.95(10.81,128.86) | 8.09(1.54,19.08) | 21.91(4.35,50.19) | 4.45(0.85,10.71) | 13.29(2.52,31.66) | 3.74(0.72,9.05) | 14.67(2.84,35.35) | 0.84(0.16,2.01) | 4.25(0.87,10.17) | 0.16(0.03,0.39) | 0.8(0.16,1.9) |
| Percentage of global, % | 100 | 100 | 46.8 | 39.87 | 25.75 | 24.18 | 21.64 | 26.7 | 4.83 | 7.74 | 0.92 | 1.46 |
| Percent change of deaths  1990–2019, % | 217.94(192.64,251.9) |  | 170.85(153.35,197.22) |  | 198.54(162.8,247.99) |  | 292.16(231.5,366.99) |  | 409.25(322.83,514.05) |  | 405.01(277.57,521.24) |  |
| All-age mortality rate |  |  |  |  |  |  |  |  |  |  |  |  |
| Rate per 100,000 | 0.65(0.12,1.55) | 1.42(0.28,3.34) | 1.94(0.37,4.57) | 4.32(0.86,9.89) | 0.77(0.15,1.85) | 1.86(0.35,4.42) | 0.44(0.09,1.07) | 1.23(0.24,2.97) | 0.15(0.03,0.36) | 0.48(0.1,1.16) | 0.06(0.01,0.15) | 0.14(0.03,0.34) |
| Percent change of rate 1990–2019, % | 118.98(101.55,142.37) |  | 122.56(108.18,144.24) |  | 141.18(112.31,181.14) |  | 177.95(134.96,230.99) |  | 221.2(166.69,287.29) |  | 134.98(75.69,189.07) |  |
| Age-standardized mortality rate |  |  |  |  |  |  |  |  |  |  |  |  |
| Rate per 100,000 | 0.83(0.16,1.98) | 1.25(0.25,2.93) | 1.3(0.25,3.06) | 1.99(0.4,4.59) | 0.73(0.14,1.77) | 1.15(0.22,2.74) | 0.78(0.15,1.86) | 1.19(0.23,2.88) | 0.32(0.06,0.78) | 0.64(0.13,1.53) | 0.16(0.03,0.39) | 0.34(0.07,0.81) |
| Percent change of rate 1990–2019, % | 50.72(38.74,66.64) |  | 52.96(44.11,67.27) |  | 56.29(38,81.92) |  | 53.91(30.92,82.46) |  | 98.1(63.88,139.19) |  | 116.86(61.44,169.89) |  |
| APC model estimates |  |  |  |  |  |  |  |  |  |  |  |  |
| Net drift of mortality y, % per year | 1.53(1.46,1.6) |  | 1.84(1.74,1.94) |  | 1.72(1.6,1.85) |  | 1.29(1.16,1.42) |  | 2.24(1.97,2.5) |  | 2.69(2.06,3.33) |  |
| **Male** | | | | | | | | | | | | |
| Deaths |  |  |  |  |  |  |  |  |  |  |  |  |
| Number*, n× 1,000 | 49.98(8.13,118.89) | 124.1(21.11,286.27) | 21.36(3.51,49.58) | 39.98(6.94,89.36) | 15.94(2.57,38.41) | 36.27(5.94,83.46) | 8.79(1.37,21.08) | 34.26(5.57,80.44) | 2.93(0.45,7.21) | 10.69(1.86,24.84) | 0.94(0.15,2.43) | 2.82(0.48,6.92) |
| Percentage of global, % | 100 | 100 | 42.74 | 32.22 | 31.88 | 29.23 | 17.58 | 27.61 | 5.87 | 8.61 | 1.87 | 2.28 |
| Percent change of deaths  1990–2019, % | 148.3(122.3,178.25) |  | 87.18(76.48,103.8) |  | 127.61(101.2,162.02) |  | 289.96(213.13,379.14) |  | 264.42(196.93,342.92) |  | 201.47(154.68,271.82) |  |
| All-age mortality rate |  |  |  |  |  |  |  |  |  |  |  |  |
| Rate per 100,000 | 1.86(0.3,4.41) | 3.2(0.54,7.38) | 5.28(0.87,12.25) | 7.91(1.37,17.67) | 2.79(0.45,6.71) | 5.08(0.83,11.68) | 1.01(0.16,2.41) | 2.84(0.46,6.67) | 0.51(0.08,1.25) | 1.21(0.21,2.8) | 0.35(0.06,0.91) | 0.5(0.08,1.22) |
| Percent change of rate 1990–2019, % | 72.34(54.3,93.13) |  | 49.85(41.28,63.15) |  | 82.24(61.09,109.79) |  | 182.26(126.64,246.81) |  | 136.86(93,187.89) |  | 41.85(19.84,74.96) |  |
| Age-standardized mortality rate |  |  |  |  |  |  |  |  |  |  |  |  |
| Rate per 100,000 | 3.02(0.49,7.16) | 3.42(0.58,7.88) | 4.86(0.8,11.26) | 4.51(0.78,10.1) | 3.62(0.59,8.67) | 4.06(0.67,9.34) | 2.04(0.32,4.87) | 3.16(0.51,7.38) | 1.11(0.17,2.69) | 1.77(0.31,4.09) | 0.91(0.14,2.34) | 1.28(0.22,3.09) |
| Percent change of rate 1990–2019, % | 13.29(2.04,26.15) |  | -7.2(-12.31,0.68) |  | 12.01(-0.58,28.46) |  | 54.77(25.68,88.56) |  | 59.45(30.18,93.57) |  | 39.61(19.18,70.57) |  |
| APC model estimates |  |  |  |  |  |  |  |  |  |  |  |  |
| Net drift of mortality y, % per year | 0.5(0.44,0.55) |  | -0.22(-0.29,-0.16) |  | 0.48(0.39,0.56) |  | 1.61(1.51,1.72) |  | 1.47(1.32,1.63) |  | 1.09(0.81,1.38) |  |

# Table S4. Trends in colon and rectal cancer mortality attributable to HPFG for both females and males across SDI quintiles, 1990−2019.

|  | **Global** | | **High SDI** | | **High-middle SDI** | | **Middle SDI** | | **Low-middle SDI** | | **Low SDI** | |
| --- | --- | --- | --- | --- | --- | --- | --- | --- | --- | --- | --- | --- |
|  | **1990** | **2019** | **1990** | **2019** | **1990** | **2019** | **1990** | **2019** | **1990** | **2019** | **1990** | **2019** |
| **Both** | | | | | | | | | | | | |
| Deaths |  |  |  |  |  |  |  |  |  |  |  |  |
| Number*, n× 1,000 | 34.09(8.14,75.1) | 97.58(23.83,212.8) | 16.62(3.99,36.31) | 33.92(8.53,73.47) | 9.95(2.32,22.05) | 27.96(6.7,61.68) | 4.87(1.16,10.94) | 22.87(5.38,50.83) | 1.96(0.46,4.39) | 10.2(2.45,22.39) | 0.67(0.15,1.51) | 2.56(0.61,5.71) |
| Percentage of global, % | 100 | 100 | 48.77 | 34.76 | 29.19 | 28.66 | 14.29 | 23.44 | 5.74 | 10.46 | 1.96 | 2.62 |
| Percent change of deaths  1990–2019, % | 186.25(166.22,214.15) |  | 104.04(91.42,123.64) |  | 181.03(157.86,215.78) |  | 369.66(316.23,435.86) |  | 421.79(343.11,516.23) |  | 282.88(218.22,368.99) |  |
| All-age mortality rate |  |  |  |  |  |  |  |  |  |  |  |  |
| Rate per 100,000 | 0.64(0.15,1.4) | 1.26(0.31,2.75) | 2.02(0.49,4.42) | 3.35(0.84,7.25) | 0.86(0.2,1.92) | 1.95(0.47,4.31) | 0.28(0.07,0.64) | 0.95(0.22,2.12) | 0.17(0.04,0.39) | 0.58(0.14,1.27) | 0.13(0.03,0.29) | 0.23(0.05,0.51) |
| Percent change of rate 1990–2019, % | 97.92(84.07,117.21) |  | 65.51(55.27,81.41) |  | 126.02(107.39,153.98) |  | 236.43(198.16,283.86) |  | 234.15(183.77,294.63) |  | 79.16(48.91,119.45) |  |
| Age-standardized mortality rate |  |  |  |  |  |  |  |  |  |  |  |  |
| Rate per 100,000 | 0.98(0.23,2.15) | 1.24(0.3,2.71) | 1.55(0.37,3.39) | 1.64(0.4,3.54) | 1.02(0.24,2.24) | 1.38(0.33,3.06) | 0.58(0.14,1.28) | 1.02(0.24,2.25) | 0.4(0.09,0.88) | 0.83(0.2,1.83) | 0.35(0.08,0.79) | 0.6(0.14,1.33) |
| Percent change of rate 1990–2019, % | 26.43(17.57,39.41) |  | 5.47(-1.42,16.17) |  | 36.25(24.63,52.74) |  | 75.83(55.38,99.58) |  | 109.74(78.1,146.59) |  | 68.59(40.87,106.22) |  |
| APC model estimates |  |  |  |  |  |  |  |  |  |  |  |  |
| Net drift of mortality y, % per year | 0.91(0.86,0.95) |  | 0.12(0.04,0.21) |  | 1.09(1.01,1.18) |  | 2.11(2.01,2.22) |  | 2.43(2.25,2.6) |  | 1.72(1.41,2.03) |  |
| **Female** | | | | | | | | | | | | |
| Deaths |  |  |  |  |  |  |  |  |  |  |  |  |
| Number*, n× 1,000 | 16.98(3.16,39.42) | 42.56(7.86,98.67) | 8.19(1.54,18.94) | 14.7(2.79,33.74) | 5.09(0.94,11.82) | 11.98(2.19,27.79) | 2.48(0.46,5.81) | 9.81(1.8,22.92) | 0.93(0.17,2.23) | 4.87(0.92,11.41) | 0.28(0.05,0.71) | 1.18(0.22,2.76) |
| Percentage of global, % | 100 | 100 | 48.23 | 34.53 | 29.98 | 28.14 | 14.59 | 23.05 | 5.48 | 11.43 | 1.65 | 2.77 |
| Percent change of deaths  1990–2019, % | 150.7(133.72,171.73) |  | 79.46(67.28,93.47) |  | 135.36(117.11,159.63) |  | 296.04(247.81,354.92) |  | 422.64(338.22,537.67) |  | 321.55(222.27,459.38) |  |
| All-age mortality rate |  |  |  |  |  |  |  |  |  |  |  |  |
| Rate per 100,000 | 0.64(0.12,1.48) | 1.1(0.2,2.56) | 1.96(0.37,4.54) | 2.89(0.55,6.65) | 0.88(0.16,2.04) | 1.67(0.31,3.88) | 0.29(0.05,0.69) | 0.82(0.15,1.93) | 0.17(0.03,0.4) | 0.55(0.1,1.3) | 0.11(0.02,0.27) | 0.21(0.04,0.49) |
| Percent change of rate 1990–2019, % | 72.66(60.97,87.15) |  | 47.47(37.46,58.98) |  | 90.15(75.4,109.75) |  | 180.71(146.52,222.44) |  | 229.64(176.4,302.19) |  | 96.15(49.96,160.28) |  |
| Age-standardized mortality rate |  |  |  |  |  |  |  |  |  |  |  |  |
| Rate per 100,000 | 0.86(0.16,1.99) | 0.97(0.18,2.25) | 1.24(0.23,2.87) | 1.2(0.23,2.74) | 0.87(0.16,2.01) | 1.02(0.19,2.36) | 0.55(0.1,1.29) | 0.82(0.15,1.91) | 0.38(0.07,0.91) | 0.75(0.14,1.75) | 0.3(0.05,0.76) | 0.53(0.1,1.24) |
| Percent change of rate 1990–2019, % | 13.16(5.88,22.48) |  | -3.25(-8.69,3.83) |  | 17.55(8.87,29.5) |  | 48.5(30.85,69.6) |  | 98.25(67.35,141.77) |  | 78.43(37.12,134.74) |  |
| APC model estimates |  |  |  |  |  |  |  |  |  |  |  |  |
| Net drift of mortality y, % per year | 0.45(0.38,0.52) |  | -0.22(-0.35,-0.1) |  | 0.47(0.34,0.6) |  | 1.37(1.21,1.52) |  | 2.2(1.95,2.45) |  | 1.97(1.5,2.45) |  |
| **Male** | | | | | | | | | | | | |
| Deaths |  |  |  |  |  |  |  |  |  |  |  |  |
| Number*, n× 1,000 | 17.11(2.96,39.64) | 55.02(10.23,123.88) | 8.44(1.49,19.4) | 19.22(3.56,42.67) | 4.86(0.83,11.36) | 15.98(2.92,36.67) | 2.39(0.42,5.66) | 13.06(2.46,30.54) | 1.02(0.18,2.47) | 5.34(1,12.32) | 0.39(0.07,0.94) | 1.38(0.25,3.3) |
| Percentage of global, % | 100 | 100 | 49.3 | 34.94 | 28.4 | 29.05 | 13.98 | 23.74 | 5.98 | 9.7 | 2.27 | 2.51 |
| Percent change of deaths  1990–2019, % | 221.53(197.65,252.94) |  | 127.91(115.84,147.31) |  | 228.85(202.84,264.86) |  | 445.88(368.87,538.78) |  | 421.02(305.81,543.89) |  | 255.07(181.27,354.37) |  |
| All-age mortality rate |  |  |  |  |  |  |  |  |  |  |  |  |
| Rate per 100,000 | 0.64(0.11,1.47) | 1.42(0.26,3.19) | 2.08(0.37,4.79) | 3.8(0.7,8.44) | 0.85(0.15,1.99) | 2.24(0.41,5.13) | 0.27(0.05,0.65) | 1.08(0.2,2.53) | 0.18(0.03,0.43) | 0.6(0.11,1.39) | 0.15(0.03,0.35) | 0.24(0.04,0.58) |
| Percent change of rate 1990–2019, % | 123.17(106.59,144.97) |  | 82.45(72.79,97.98) |  | 163.29(142.47,192.12) |  | 295.11(239.37,362.36) |  | 238.65(163.77,318.51) |  | 67.07(32.35,113.8) |  |
| Age-standardized mortality rate |  |  |  |  |  |  |  |  |  |  |  |  |
| Rate per 100,000 | 1.15(0.2,2.65) | 1.58(0.3,3.56) | 2.02(0.35,4.64) | 2.18(0.4,4.84) | 1.26(0.22,2.92) | 1.9(0.35,4.34) | 0.61(0.11,1.43) | 1.25(0.24,2.92) | 0.41(0.07,0.99) | 0.92(0.18,2.1) | 0.41(0.07,0.99) | 0.67(0.12,1.58) |
| Percent change of rate 1990–2019, % | 37.22(27.54,49.44) |  | 8.07(2.56,16.71) |  | 50.83(39.57,66.35) |  | 104.31(76.91,134.93) |  | 122.72(75.26,174.41) |  | 62.42(30.92,107.21) |  |
| APC model estimates |  |  |  |  |  |  |  |  |  |  |  |  |
| Net drift of mortality y, % per year | 1.22(1.15,1.28) |  | 0.22(0.12,0.32) |  | 1.48(1.36,1.59) |  | 2.74(2.59,2.89) |  | 2.66(2.42,2.9) |  | 1.57(1.15,1.98) |  |

# Table S5. Trends in breast cancer mortality attributable to HPFG for female across SDI quintiles, 1990−2019.

|  | **Global** | | **High SDI** | | **High-middle SDI** | | **Middle SDI** | | **Low-middle SDI** | | **Low SDI** | |
| --- | --- | --- | --- | --- | --- | --- | --- | --- | --- | --- | --- | --- |
|  | **1990** | **2019** | **1990** | **2019** | **1990** | **2019** | **1990** | **2019** | **1990** | **2019** | **1990** | **2019** |
| **Female** | | | | | | | | | | | | |
| Deaths |  |  |  |  |  |  |  |  |  |  |  |  |
| Number*, n× 1,000 | 19.61(3.73,43.21) | 51.06(9.9,113.53) | 8.43(1.65,18.64) | 15.01(3.03,32.29) | 5.26(1,11.76) | 11.91(2.3,26.31) | 3.34(0.63,7.51) | 12.51(2.42,27.85) | 1.9(0.35,4.44) | 8.61(1.63,19.68) | 0.67(0.12,1.61) | 2.98(0.57,6.88) |
| Percentage of global, % | 100 | 100 | 42.98 | 29.39 | 26.83 | 23.33 | 17.01 | 24.49 | 9.67 | 16.85 | 3.43 | 5.84 |
| Percent change of deaths  1990–2019, % | 160.41(140.91,184.43) |  | 78.1(67.35,93.29) |  | 126.37(110.81,147.69) |  | 274.96(233.26,326.73) |  | 354.02(272.74,473.99) |  | 343.51(255.69,468.95) |  |
| All-age mortality rate |  |  |  |  |  |  |  |  |  |  |  |  |
| Rate per 100,000 | 0.74(0.14,1.63) | 1.32(0.26,2.94) | 2.02(0.4,4.47) | 2.96(0.6,6.36) | 0.91(0.17,2.03) | 1.66(0.32,3.67) | 0.4(0.07,0.89) | 1.05(0.2,2.34) | 0.34(0.06,0.8) | 0.98(0.19,2.24) | 0.26(0.05,0.61) | 0.53(0.1,1.22) |
| Percent change of rate 1990–2019, % | 79.35(65.92,95.9) |  | 46.35(37.52,58.83) |  | 82.88(70.31,100.1) |  | 165.76(136.2,202.46) |  | 186.36(135.1,262.03) |  | 106.37(65.5,164.74) |  |
| Age-standardized mortality rate |  |  |  |  |  |  |  |  |  |  |  |  |
| Rate per 100,000 | 0.95(0.18,2.11) | 1.17(0.23,2.59) | 1.37(0.27,3.04) | 1.38(0.27,2.96) | 0.89(0.17,2) | 1.04(0.2,2.31) | 0.66(0.12,1.47) | 0.97(0.19,2.16) | 0.66(0.12,1.53) | 1.22(0.23,2.79) | 0.61(0.11,1.44) | 1.18(0.23,2.69) |
| Percent change of rate 1990–2019, % | 22.32(13.39,32.97) |  | 0.54(-4.9,8.21) |  | 17.23(9.5,27.86) |  | 46.65(29.89,66.01) |  | 84.59(50.42,133.82) |  | 91.98(53.88,149.04) |  |
| APC model estimates |  |  |  |  |  |  |  |  |  |  |  |  |
| Net drift of mortality y, % per year | 0.74(0.68,0.8) |  | 0.17(0.07,0.27) |  | 0.55(0.44,0.67) |  | 1.24(1.1,1.38) |  | 2(1.8,2.2) |  | 2.25(1.92,2.59) |  |

# Table S6. Trends in pancreatic cancer mortality attributable to HPFG for both females and males across SDI quintiles, 1990−2019.

|  | **Global** | | **High SDI** | | **High-middle SDI** | | **Middle SDI** | | **Low-middle SDI** | | **Low SDI** | |
| --- | --- | --- | --- | --- | --- | --- | --- | --- | --- | --- | --- | --- |
|  | **1990** | **2019** | **1990** | **2019** | **1990** | **2019** | **1990** | **2019** | **1990** | **2019** | **1990** | **2019** |
| **Both** | | | | | | | | | | | | |
| Deaths |  |  |  |  |  |  |  |  |  |  |  |  |
| Number*, n× 1,000 | 13.07(2.99,28.68) | 48.36(11.54,103.69) | 6.55(1.51,14.35) | 19.64(4.76,41.73) | 3.95(0.89,8.67) | 13.21(3.08,28.8) | 1.77(0.39,3.89) | 10.14(2.34,21.94) | 0.6(0.13,1.41) | 4.36(1.03,9.46) | 0.19(0.04,0.46) | 0.97(0.22,2.17) |
| Percentage of global, % | 100 | 100 | 50.17 | 40.62 | 30.21 | 27.33 | 13.58 | 20.97 | 4.57 | 9.02 | 1.43 | 2.01 |
| Percent change of deaths  1990–2019, % | 270.12(247.22,304.77) |  | 199.7(179.96,228.91) |  | 234.8(208.49,268.56) |  | 471.42(406.47,553.59) |  | 629.85(519.64,809.27) |  | 420.5(329.82,546.98) |  |
| All-age mortality rate |  |  |  |  |  |  |  |  |  |  |  |  |
| Rate per 100,000 | 0.24(0.06,0.54) | 0.62(0.15,1.34) | 0.8(0.18,1.75) | 1.94(0.47,4.12) | 0.34(0.08,0.75) | 0.92(0.22,2.01) | 0.1(0.02,0.23) | 0.42(0.1,0.92) | 0.05(0.01,0.12) | 0.25(0.06,0.54) | 0.04(0.01,0.09) | 0.09(0.02,0.19) |
| Percent change of rate 1990–2019, % | 155.91(140.08,179.86) |  | 143.1(127.09,166.8) |  | 169.27(148.11,196.42) |  | 309.33(262.81,368.19) |  | 367.4(296.82,482.29) |  | 143.56(101.12,202.74) |  |
| Age-standardized mortality rate |  |  |  |  |  |  |  |  |  |  |  |  |
| Rate per 100,000 | 0.36(0.08,0.8) | 0.61(0.14,1.3) | 0.61(0.14,1.34) | 0.97(0.23,2.07) | 0.39(0.09,0.86) | 0.65(0.15,1.41) | 0.2(0.05,0.44) | 0.44(0.1,0.95) | 0.12(0.03,0.28) | 0.35(0.08,0.75) | 0.1(0.02,0.23) | 0.22(0.05,0.49) |
| Percent change of rate 1990–2019, % | 66.92(56.16,82.97) |  | 59.56(49.77,75.26) |  | 65.63(52.81,83.15) |  | 117.42(93.53,147.52) |  | 194.33(151.59,266.33) |  | 131.59(93.44,186.15) |  |
| APC model estimates |  |  |  |  |  |  |  |  |  |  |  |  |
| Net drift of mortality y, % per year | 1.99(1.92,2.06) |  | 1.93(1.81,2.04) |  | 1.91(1.79,2.04) |  | 2.68(2.5,2.85) |  | 3.74(3.43,4.05) |  | 2.94(2.36,3.52) |  |
| **Female** | | | | | | | | | | | | |
| Deaths |  |  |  |  |  |  |  |  |  |  |  |  |
| Number*, n× 1,000 | 6.36(1.05,14.84) | 22.42(3.74,52.36) | 3.23(0.53,7.55) | 9(1.54,20.96) | 1.95(0.32,4.59) | 6.1(1,14.31) | 0.86(0.14,2.02) | 4.73(0.78,11.06) | 0.25(0.04,0.61) | 2.14(0.37,4.98) | 0.06(0.01,0.16) | 0.44(0.07,1.06) |
| Percentage of global, % | 100 | 100 | 50.82 | 40.13 | 30.61 | 27.21 | 13.54 | 21.09 | 3.97 | 9.54 | 1.02 | 1.97 |
| Percent change of deaths  1990–2019, % | 252.53(226.15,283.64) |  | 178.38(159.57,203.51) |  | 213.37(185.71,246.85) |  | 449.35(382.82,527.03) |  | 746.15(593.57,925.76) |  | 581.63(436.31,739.3) |  |
| All-age mortality rate |  |  |  |  |  |  |  |  |  |  |  |  |
| Rate per 100,000 | 0.24(0.04,0.56) | 0.58(0.1,1.36) | 0.77(0.13,1.81) | 1.77(0.3,4.13) | 0.34(0.06,0.79) | 0.85(0.14,2) | 0.1(0.02,0.24) | 0.4(0.07,0.93) | 0.05(0.01,0.11) | 0.24(0.04,0.57) | 0.02(0,0.06) | 0.08(0.01,0.19) |
| Percent change of rate 1990–2019, % | 142.8(124.63,164.23) |  | 128.75(113.3,149.4) |  | 153.17(130.82,180.22) |  | 289.37(242.21,344.42) |  | 433.68(337.45,546.97) |  | 217.16(149.55,290.53) |  |
| Age-standardized mortality rate |  |  |  |  |  |  |  |  |  |  |  |  |
| Rate per 100,000 | 0.31(0.05,0.74) | 0.51(0.09,1.19) | 0.49(0.08,1.15) | 0.77(0.13,1.8) | 0.33(0.05,0.77) | 0.52(0.09,1.22) | 0.19(0.03,0.43) | 0.39(0.06,0.9) | 0.1(0.02,0.25) | 0.33(0.06,0.76) | 0.07(0.01,0.17) | 0.2(0.03,0.47) |
| Percent change of rate 1990–2019, % | 62.08(50.32,76.06) |  | 56.72(47.23,70.22) |  | 59.76(46.25,76.51) |  | 107.44(81.71,135.77) |  | 219.32(161.33,287.97) |  | 189.02(126.04,256.07) |  |
| APC model estimates |  |  |  |  |  |  |  |  |  |  |  |  |
| Net drift of mortality y, % per year | 1.89(1.78,2) |  | 1.88(1.7,2.06) |  | 1.76(1.56,1.95) |  | 2.39(2.14,2.64) |  | 4.07(3.61,4.53) |  | 3.84(2.9,4.79) |  |
| **Male** | | | | | | | | | | | | |
| Deaths |  |  |  |  |  |  |  |  |  |  |  |  |
| Number*, n× 1,000 | 6.71(1.21,15.79) | 25.94(4.86,59.1) | 3.32(0.61,7.8) | 10.65(2.06,23.75) | 2(0.36,4.77) | 7.12(1.31,16.62) | 0.91(0.17,2.2) | 5.41(1,12.68) | 0.34(0.06,0.86) | 2.22(0.43,5.24) | 0.12(0.02,0.32) | 0.53(0.1,1.28) |
| Percentage of global, % | 100 | 100 | 49.55 | 41.05 | 29.83 | 27.43 | 13.63 | 20.86 | 5.14 | 8.57 | 1.81 | 2.04 |
| Percent change of deaths  1990–2019, % | 286.8(257.41,328.93) |  | 220.44(200.72,248.6) |  | 255.65(221.43,302.36) |  | 492.2(393.65,616.8) |  | 544.68(418.9,753.2) |  | 334.58(241.33,471.49) |  |
| All-age mortality rate |  |  |  |  |  |  |  |  |  |  |  |  |
| Rate per 100,000 | 0.25(0.05,0.59) | 0.67(0.13,1.52) | 0.82(0.15,1.93) | 2.11(0.41,4.7) | 0.35(0.06,0.83) | 1(0.18,2.33) | 0.1(0.02,0.25) | 0.45(0.08,1.05) | 0.06(0.01,0.15) | 0.25(0.05,0.59) | 0.05(0.01,0.12) | 0.09(0.02,0.23) |
| Percent change of rate 1990–2019, % | 168.47(148.08,197.71) |  | 156.52(140.74,179.07) |  | 184.75(157.35,222.14) |  | 328.64(257.31,418.83) |  | 319.03(237.27,454.56) |  | 104.49(60.61,168.91) |  |
| Age-standardized mortality rate |  |  |  |  |  |  |  |  |  |  |  |  |
| Rate per 100,000 | 0.42(0.08,1) | 0.72(0.14,1.64) | 0.77(0.14,1.8) | 1.21(0.23,2.7) | 0.48(0.08,1.14) | 0.8(0.15,1.88) | 0.22(0.04,0.52) | 0.5(0.09,1.16) | 0.14(0.02,0.34) | 0.37(0.07,0.88) | 0.12(0.02,0.31) | 0.25(0.05,0.59) |
| Percent change of rate 1990–2019, % | 70.41(58.35,88.55) |  | 57.13(47.66,70.44) |  | 67.84(52.57,88.47) |  | 126.98(91.52,170.08) |  | 176.73(124.94,265.51) |  | 100.72(60.38,161.35) |  |
| APC model estimates |  |  |  |  |  |  |  |  |  |  |  |  |
| Net drift of mortality y, % per year | 2.07(1.98,2.17) |  | 1.88(1.73,2.03) |  | 1.98(1.81,2.16) |  | 2.94(2.69,3.19) |  | 3.51(3.09,3.93) |  | 2.4(1.66,3.14) |  |

# Table S7. Trends in cancer mortality attributable to HPFG for both genders in 204 countries and regions, 1990-2019.

| **SDI quintile** | **Country** | **Deaths** | | **All-age mortality** | | **Age-standardized mortality** | | **Net drift of mortality from APC model,% per year** |
| --- | --- | --- | --- | --- | --- | --- | --- | --- |
|  |  | **Number in 2019** | **Percent change 1990-2019, %** | **Rate in 2019** | **Percent change 1990-2019, %** | **Rate in 2019** | **Percent change 1990-2019, %** |  |
| High SDI | Denmark | 852.67(238.05,1765.62) | 133.51(109.02,171.23) | 14.69(4.1,30.43) | 107.01(85.3,140.45) | 6.88(1.91,14.3) | 58.69(42.37,83.5) | 1.11(0.62,1.6) |
|  | Puerto Rico | 513.87(152.59,1112.03) | 175.48(117.3,254.1) | 14.59(4.33,31.58) | 182.67(122.97,263.34) | 6.73(1.97,14.7) | 30.2(1.83,67.71) | 0.85(0.24,1.46) |
|  | Switzerland | 982.2(275.33,2013.04) | 70.84(52,98.61) | 11.19(3.14,22.94) | 33.66(18.92,55.38) | 5.23(1.46,10.73) | -1.31(-11.88,14.72) | -0.18(-0.61,0.25) |
|  | Russian Federation | 8133.34(2123.91,17355.24) | 72.74(52.34,102.28) | 5.54(1.45,11.83) | 77.81(56.81,108.22) | 3.39(0.88,7.23) | 30.23(15.09,52.01) | 0.86(0.73,1) |
|  | Germany | 19415.79(5634.34,38268.51) | 92.78(73.93,121.05) | 22.87(6.64,45.07) | 81.49(63.75,108.11) | 9.35(2.73,18.53) | 22.74(10.82,40.52) | 0.34(0.08,0.6) |
|  | Finland | 861.63(247.76,1734.68) | 107.66(87.89,136.32) | 15.57(4.48,31.35) | 88(70.1,113.95) | 6.43(1.85,12.97) | 13.69(3.34,28.89) | 0.39(-0.1,0.89) |
|  | France | 6324.85(1676.57,13272.61) | 124.13(101.78,153.51) | 9.55(2.53,20.05) | 95.58(76.08,121.21) | 4.24(1.11,8.91) | 30.57(18.54,46.9) | 0.93(0.75,1.11) |
|  | Czechia | 2692.06(797.65,5580.93) | 85.04(50.64,135.82) | 25.29(7.49,52.44) | 79.03(45.75,128.15) | 12.1(3.57,25.22) | 16.31(-5.72,47.88) | 0.29(0.03,0.54) |
|  | United Kingdom | 13805.92(4055.56,27142.98) | 79.13(65.13,104.97) | 20.54(6.03,40.38) | 53.15(41.19,75.24) | 10.15(2.95,20.01) | 25.23(16.02,42.51) | 0.49(0.37,0.61) |
|  | San Marino | 5.49(1.41,13.38) | 224.62(119.51,374.05) | 16.59(4.25,40.41) | 131.02(56.22,237.37) | 7.74(1.97,19.12) | 56.07(2.77,130.07) | 1.82(-5.12,9.27) |
|  | Brunei Darussalam | 44.7(13.8,85.84) | 227.2(171.66,304.59) | 10.23(3.16,19.64) | 93.53(60.68,139.3) | 19.87(6.19,37.39) | 9.82(-7.44,33.19) | 0.71(-1.57,3.04) |
|  | Kuwait | 122.78(36.46,247.62) | 513.38(419.13,656.16) | 2.77(0.82,5.59) | 143.8(106.34,200.55) | 6.2(1.86,12.43) | 45.75(22.95,82.44) | 1.5(-0.27,3.3) |
|  | Ireland | 513.38(142.76,1049) | 268.02(231.05,332.24) | 10.46(2.91,21.36) | 169.91(142.79,217) | 6.61(1.83,13.52) | 99.53(79.58,133.4) | 2.2(1.43,2.96) |
|  | Republic of Korea | 4667.05(1218.08,9587.79) | 426.96(368,502.46) | 8.74(2.28,17.96) | 337.55(288.59,400.24) | 5.27(1.37,10.81) | 60.09(42.42,81.99) | 0.34(0.08,0.59) |
|  | Taiwan (Province of China) | 2758.47(731.72,5951.25) | 430.71(320.68,595.41) | 11.68(3.1,25.2) | 358.34(263.31,500.58) | 6.91(1.83,14.91) | 94.76(54.51,153.19) | 1.98(1.64,2.31) |
|  | Luxembourg | 94.86(27.72,192.49) | 294.57(237.65,393.44) | 15.34(4.48,31.12) | 143.21(108.12,204.15) | 9.08(2.63,18.46) | 111.29(81.81,163.57) | 2.66(0.92,4.44) |
|  | Singapore | 363.38(98.26,750.29) | 134.16(108.74,159.88) | 6.41(1.73,13.24) | 25.9(12.24,39.73) | 4.89(1.32,10.07) | -37.39(-44.11,-30.62) | -2.27(-2.94,-1.59) |
|  | Slovenia | 309.38(81.69,674.11) | 106.5(46.13,185.31) | 14.92(3.94,32.5) | 96.23(38.86,171.12) | 6.72(1.77,14.67) | 9.59(-23.46,51.91) | -0.24(-0.96,0.49) |
|  | United States of America | 58134.43(16746.97,114099.58) | 104.62(92.23,127.61) | 17.73(5.11,34.79) | 58.22(48.64,76) | 9.97(2.86,19.61) | 14.61(7.63,27.31) | 1.19(1.1,1.29) |
|  | Australia | 2173.1(587.91,4443.84) | 172.18(145.81,214.32) | 8.85(2.39,18.09) | 86.79(68.69,115.7) | 4.84(1.31,9.88) | 18.17(7.54,35.32) | 0.12(-0.19,0.44) |
|  | Iceland | 29.34(8.19,61.22) | 174.89(143.7,217.86) | 8.51(2.37,17.75) | 102.43(79.46,134.07) | 5.01(1.39,10.47) | 38.51(22.82,60.08) | 0.85(-1.86,3.63) |
|  | Canada | 4148.72(1122.38,8614.43) | 185.39(155.56,227.09) | 11.36(3.07,23.59) | 113(90.74,144.13) | 5.7(1.53,11.8) | 30(17.21,48.88) | 0.32(0.09,0.56) |
|  | Japan | 15793.96(4102.22,32973.42) | 148.77(119.17,169.9) | 12.36(3.21,25.8) | 145.03(115.88,165.84) | 3.74(0.98,7.76) | -1.78(-9.37,4.62) | -0.71(-0.83,-0.59) |
|  | Slovakia | 656.69(173.88,1444.73) | 97.1(53.45,156.79) | 12.08(3.2,26.57) | 91.51(49.09,149.5) | 6.94(1.84,15.29) | 25.86(-1.81,64.19) | 0.65(0.14,1.15) |
|  | New Zealand | 399.97(111.27,820.15) | 145.22(124.19,179.77) | 8.9(2.47,18.24) | 86.4(70.41,112.66) | 4.83(1.34,9.93) | 16.94(7.63,32.46) | -0.57(-1.29,0.15) |
|  | Estonia | 139.72(35.8,305.76) | 73.45(37.36,122.19) | 10.65(2.73,23.3) | 107.33(64.19,165.59) | 4.95(1.28,10.87) | 26.88(-0.75,61.97) | 1.03(0,2.07) |
|  | Belgium | 1739.75(485.51,3551.95) | 65.33(50.6,90.37) | 15.24(4.25,31.11) | 44.49(31.62,66.37) | 7.03(1.93,14.41) | 6.89(-2.53,22.19) | 0.01(-0.31,0.33) |
|  | Qatar | 72.81(22.16,147.66) | 1127.83(803.62,1661.83) | 2.54(0.77,5.15) | 90.8(40.42,173.78) | 15.77(5.09,30.35) | 74.67(32.45,147.63) | 1.43(-1.52,4.48) |
|  | Saudi Arabia | 680.38(190.94,1415.55) | 478.62(327.22,708.94) | 1.9(0.53,3.96) | 159.82(91.84,263.25) | 4.88(1.43,9.97) | 103.64(56.53,184.51) | 2.14(1.47,2.81) |
|  | Latvia | 200.19(51.75,431.8) | 71.69(42.69,110.53) | 10.45(2.7,22.54) | 138.33(98.08,192.24) | 4.79(1.24,10.4) | 48.57(23.26,82.15) | 1.82(0.98,2.68) |
|  | Austria | 1022.51(284.36,2097.11) | 97.08(79.01,123.64) | 11.47(3.19,23.52) | 71.71(55.97,94.85) | 5.37(1.47,11.03) | 29.04(17.9,46.16) | 0.71(0.28,1.13) |
|  | Andorra | 10.11(2.53,22.13) | 277.85(173.98,429.97) | 12.17(3.04,26.64) | 146.06(78.42,245.12) | 7.03(1.75,15.34) | 33.21(-3.49,84.23) | 0.89(-3.9,5.91) |
|  | Netherlands | 2444.4(671.01,5094.57) | 90.48(70.06,120.38) | 14.25(3.91,29.69) | 65.68(47.92,91.69) | 6.71(1.84,14) | 8.08(-3.34,24.96) | -0.16(-0.44,0.11) |
|  | United Arab Emirates | 280.03(77.35,588.44) | 1236.38(876.19,1763.71) | 3.03(0.84,6.37) | 170.71(97.75,277.53) | 12.85(3.83,25.67) | 46.29(13.23,99.41) | 1.44(-0.26,3.16) |
|  | Lithuania | 216.45(53.62,471.39) | 59.52(29.38,96.96) | 7.75(1.92,16.87) | 109.72(70.1,158.94) | 3.61(0.89,7.86) | 20.52(-2.93,49.11) | 0.93(0.15,1.72) |
|  | Bermuda | 9.21(2.52,19.78) | 126.99(91.65,184.77) | 14.38(3.93,30.89) | 110.76(77.95,164.41) | 6.64(1.81,14.25) | -3.02(-17.43,21.7) | -0.47(-5.01,4.29) |
|  | Cyprus | 162.65(45.42,325.97) | 206.29(163.5,261.47) | 12.38(3.46,24.82) | 81.42(56.08,114.11) | 8.3(2.32,16.62) | 21.77(3.9,42.85) | 0.47(-0.76,1.72) |
|  | Guam | 12.66(3.51,27.06) | 256.34(190.62,353.33) | 7.42(2.05,15.86) | 185.61(132.93,263.35) | 6.8(1.89,14.49) | 13.27(-8.82,44.74) | 0.64(-3.5,4.97) |
|  | Norway | 699.34(200.71,1408.81) | 71.32(60.33,89.13) | 13.07(3.75,26.34) | 36.03(27.3,50.16) | 6.78(1.93,13.68) | 20.96(13.22,33.56) | -0.08(-0.62,0.46) |
|  | Sweden | 1244.73(352.67,2520.36) | 91.74(76.05,117.67) | 12.18(3.45,24.65) | 61.1(47.91,82.88) | 5.36(1.5,10.87) | 33.54(23.04,51.61) | 0.84(0.41,1.27) |
|  | Monaco | 11.81(3.16,24.95) | 207.93(144.17,305.65) | 31.44(8.4,66.41) | 149.48(97.82,228.65) | 11.34(3.01,24.17) | 127.43(77.61,203.5) | 3.14(-1.58,8.1) |
| High middle SDI | Dominica | 8.37(2.38,17.12) | 123.22(83.88,184.78) | 12.19(3.46,24.92) | 140.52(98.13,206.85) | 9.14(2.59,18.75) | 83.7(51.31,135.49) | 2.32(-2.29,7.14) |
|  | Croatia | 871.28(234.69,1904.99) | 100.19(59.31,158) | 20.51(5.52,44.85) | 130.95(83.79,197.64) | 9.37(2.51,20.63) | 34.77(7.26,72.33) | 1.19(0.75,1.63) |
|  | Ukraine | 2637.71(675.62,5703.77) | 13.37(-4.49,37.18) | 5.99(1.53,12.95) | 35.56(14.2,64.03) | 3.42(0.88,7.4) | 6.81(-10.1,29.05) | -0.44(-0.64,-0.23) |
|  | Hungary | 2249.58(619.43,4803.42) | 96.43(63.01,144.45) | 23.25(6.4,49.65) | 111.01(75.11,162.59) | 11.23(3.06,24.15) | 44.6(19.78,80) | 1.2(0.94,1.46) |
|  | Argentina | 4220.89(1167.99,8538) | 159.89(137.52,196.41) | 9.36(2.59,18.92) | 90.79(74.37,117.6) | 7.65(2.11,15.48) | 50.09(37.85,69.93) | 0.99(0.77,1.2) |
|  | Saint Kitts and Nevis | 4.88(1.41,10.1) | 88.28(56.81,130.71) | 8.2(2.38,16.97) | 30.84(8.97,60.32) | 8.4(2.45,17.29) | 21.86(3.26,48.57) | 0.98(-5,7.34) |
|  | North Macedonia | 383.87(106.08,826.05) | 313.87(229.03,437.85) | 17.83(4.93,38.37) | 287.48(208.05,403.55) | 11.9(3.3,25.58) | 131.79(84.9,199.6) | 3.08(2.25,3.93) |
|  | Kazakhstan | 907.12(243.87,1894.01) | 89.32(59.8,132.63) | 4.93(1.33,10.3) | 68.49(42.21,107.02) | 5.49(1.48,11.38) | 42.5(20.88,74.59) | 2.01(1.57,2.46) |
|  | Romania | 2117.78(540.72,4531.09) | 180.8(128.96,250.15) | 11.01(2.81,23.55) | 241.51(178.47,325.86) | 5.56(1.42,11.96) | 109.1(71.7,160.86) | 2.45(2.14,2.77) |
|  | Chile | 1411.69(405.43,2850.33) | 312.25(270.6,380.67) | 7.76(2.23,15.66) | 200.83(170.45,250.77) | 5.84(1.68,11.79) | 58.67(42.97,84.05) | 1.72(1.29,2.16) |
|  | Poland | 7265.41(1933.32,15359.88) | 139.61(104.6,185.5) | 18.9(5.03,39.96) | 137.89(103.13,183.44) | 9.99(2.65,21.21) | 44.01(22.57,71.41) | 1.23(1.07,1.38) |
|  | Montenegro | 116.65(30.87,248.55) | 192.43(142.35,262.4) | 18.8(4.98,40.07) | 194.97(144.45,265.55) | 11.6(3.07,24.62) | 76.05(46.31,116.7) | 2.13(0.85,3.43) |
|  | Greece | 1901.06(513.85,3920.66) | 143.87(119.58,178.6) | 18.39(4.97,37.93) | 145.11(120.69,180.01) | 7.35(1.96,15.3) | 46.63(33.48,66) | 1.26(0.92,1.59) |
|  | Malaysia | 1701.31(461.04,3664.6) | 412.87(295.97,573.92) | 5.44(1.47,11.71) | 189.28(123.34,280.11) | 7.28(2.01,15.45) | 75.25(34.27,131.51) | 1.69(1.28,2.09) |
|  | United States Virgin Islands | 20.25(5.81,40.74) | 406.76(318.21,536.74) | 19.47(5.59,39.18) | 416.64(326.36,549.15) | 10.89(3.1,21.95) | 104.07(69.19,154.92) | 2.92(-0.71,6.68) |
|  | Lebanon | 564.41(160.34,1168.1) | 421.33(318.94,644.67) | 10.9(3.1,22.56) | 229.8(165.03,371.09) | 10.86(3.09,22.42) | 104.02(65.12,189.27) | 3.05(2.32,3.78) |
|  | Serbia | 2008.86(553.97,4339.95) | 178(121.33,273) | 22.97(6.33,49.62) | 198.68(137.79,300.75) | 12.15(3.35,26.27) | 86.5(49.06,147.33) | 2.27(1.95,2.59) |
|  | Antigua and Barbuda | 6.93(2.04,14.4) | 226(178.96,287.86) | 7.84(2.31,16.28) | 123.71(91.43,166.16) | 7.38(2.18,15.28) | 93.39(68.13,133.08) | 2.23(-3.33,8.13) |
|  | Israel | 784.25(224.47,1584.54) | 220.09(193.34,260.37) | 8.42(2.41,17.02) | 70.6(56.34,92.07) | 6.47(1.85,13.07) | 29.22(18.79,44.96) | 0.23(-0.3,0.76) |
|  | Bulgaria | 1155.74(309.13,2571.6) | 121.64(76.95,179.35) | 16.67(4.46,37.08) | 177.47(121.52,249.71) | 7.76(2.06,17.46) | 88.21(49,137.81) | 3.75(3.34,4.17) |
|  | Italy | 11865.73(3407.66,23645.51) | 90.68(74.69,111.66) | 19.67(5.65,39.2) | 79.57(64.51,99.32) | 7.5(2.14,14.91) | 10.76(3.23,22.06) | 0.33(0.2,0.46) |
|  | Barbados | 42.26(12.12,88.61) | 191.74(143.43,253.91) | 14.19(4.07,29.76) | 148.75(107.56,201.76) | 8.43(2.41,17.68) | 79.44(49.68,118.35) | 2.06(-0.26,4.43) |
|  | Trinidad and Tobago | 135.5(38.53,295.79) | 178.41(112.3,275.59) | 9.77(2.78,21.32) | 141.4(84.08,225.66) | 7.36(2.1,16.01) | 19.14(-9.36,60.38) | 0.56(-0.6,1.72) |
|  | Bahamas | 26.27(7.47,55.2) | 270.18(202.59,361.14) | 6.97(1.98,14.64) | 151.86(105.87,213.75) | 7.18(2.07,14.88) | 44.57(18.96,78.82) | 1.48(-1.46,4.52) |
|  | Bosnia and Herzegovina | 710.56(200.06,1521.56) | 292.84(206.54,423.32) | 21.53(6.06,46.11) | 440.21(321.54,619.64) | 11.54(3.25,24.75) | 142.24(88.99,218.29) | 3.53(2.92,4.15) |
|  | Malta | 70.42(20.28,143.03) | 135.97(109.19,178.18) | 16.03(4.62,32.56) | 99.15(76.55,134.77) | 7.08(2.02,14.38) | 0.83(-10.76,18.08) | -0.11(-1.79,1.6) |
|  | Jordan | 334.89(92.87,695.66) | 638.03(498.43,849.41) | 2.88(0.8,5.98) | 139.3(94.04,207.84) | 6.12(1.71,12.56) | 52.56(23.89,95.81) | 1.38(0.28,2.5) |
|  | Belarus | 463.09(113.44,1051.56) | 28.51(0.36,65.08) | 4.87(1.19,11.07) | 41.62(10.6,81.93) | 2.86(0.7,6.49) | 3.81(-18.93,33.8) | -0.61(-1.1,-0.12) |
|  | Seychelles | 11.43(3.47,22.42) | 348.51(282.41,455.29) | 11.19(3.39,21.94) | 220.66(173.4,297) | 11.61(3.56,22.59) | 156.75(119.68,219.71) | 3(-1.78,8.02) |
|  | Portugal | 1967.22(572.04,3897.72) | 155.85(131.46,193.52) | 18.47(5.37,36.59) | 143.5(120.29,179.35) | 7.55(2.19,14.98) | 36.68(25.15,56.12) | 1.13(0.81,1.45) |
|  | Cook Islands | 2.66(0.75,5.45) | 194.21(136.68,283.05) | 14.79(4.19,30.28) | 211.14(150.29,305.09) | 10.76(3.04,22.15) | 36.87(10.53,76.78) | 0.99(-7.12,9.81) |
|  | Libya | 370.19(102.3,777.14) | 427.36(301.6,647.58) | 5.5(1.52,11.54) | 231.73(152.62,370.26) | 8.09(2.25,16.83) | 93.47(48.39,172.07) | 2.47(1.57,3.37) |
|  | American Samoa | 6.91(2.16,13.43) | 254.79(189.85,349.75) | 12.44(3.9,24.19) | 209.58(152.91,292.43) | 15.48(4.89,29.86) | 52.65(26.31,91.54) | 1.55(-3.98,7.4) |
|  | Turkey | 5434.24(1366.82,11696.18) | 265.23(180.07,389.54) | 6.68(1.68,14.38) | 168.33(105.76,259.65) | 6.36(1.61,13.68) | 43.06(9.65,91.7) | 3.14(2.88,3.4) |
|  | Bahrain | 83.68(25.25,165.8) | 404.69(280.58,603.85) | 5.8(1.75,11.49) | 77.73(34.02,147.86) | 13.74(4.31,26.51) | 3.2(-19.77,42.55) | -0.72(-2.6,1.21) |
|  | Spain | 7951.87(2243.86,16230.28) | 109.33(87.45,134.84) | 17.28(4.88,35.27) | 76.4(57.96,97.9) | 7.65(2.13,15.63) | 13.59(2.7,26.31) | 0.19(0.04,0.34) |
|  | Oman | 62.25(17.96,126.83) | 425.89(311.28,632.86) | 1.36(0.39,2.77) | 122.93(74.34,210.67) | 5.18(1.53,10.39) | 136.37(82.66,226.93) | 2.93(0.63,5.29) |
|  | Greenland | 7.18(1.87,15.72) | 335.45(254.17,446.44) | 12.78(3.33,27.97) | 330.72(250.31,440.5) | 11.24(2.96,24.45) | 101.59(65.92,149.2) | 2.04(-4.11,8.59) |
|  | Niue | 0.29(0.09,0.59) | 70.08(31.33,124.48) | 17.46(5.15,35.5) | 136.73(82.8,212.44) | 13.47(3.98,27.4) | 78.14(38.37,134.62) | 1.98(-17.43,25.95) |
|  | Northern Mariana Islands | 5.56(1.52,11.38) | 397.12(312.17,523.45) | 13.08(3.57,26.78) | 431.08(340.32,566.04) | 12.48(3.39,25.26) | 42.33(20.21,75.1) | 1.32(-6.06,9.29) |
|  | Palau | 2.75(0.79,5.7) | 263.27(169.07,393.33) | 15.29(4.4,31.65) | 210.75(130.17,322.01) | 14.58(4.23,29.93) | 70.9(27.46,132.35) | 1.87(-6.93,11.5) |
| Middle SDI | Costa Rica | 268.22(75.48,589.88) | 393.78(286.95,538.07) | 5.69(1.6,12.51) | 218.24(149.39,311.23) | 5.3(1.49,11.65) | 59.36(24.86,105.59) | 1.38(0.35,2.43) |
|  | Iran (Islamic Republic of) | 2688.93(777.06,5381.91) | 590.6(499.74,748.65) | 3.19(0.92,6.38) | 379.58(316.48,489.34) | 4.05(1.18,8.08) | 122.67(92.74,174.05) | 3.16(2.75,3.57) |
|  | Gabon | 59.7(15.23,133.97) | 180.15(114.73,255.02) | 3.41(0.87,7.66) | 58.73(21.66,101.15) | 6.56(1.69,14.53) | 56.84(22.32,96.7) | 1.49(-0.27,3.28) |
|  | Armenia | 288.14(76.66,621.01) | 173.71(129.26,238.24) | 9.54(2.54,20.57) | 209.5(159.24,282.47) | 6.86(1.83,14.73) | 73.88(46.29,114.86) | 1.9(1.05,2.76) |
|  | Azerbaijan | 411.06(108.18,884.89) | 281.13(211.26,385.53) | 4(1.05,8.61) | 171.83(122,246.29) | 4.68(1.23,10.01) | 115.2(74.48,179.31) | 3.66(2.76,4.56) |
|  | Grenada | 9.89(2.95,19.42) | 168.87(135.72,219.15) | 9.58(2.86,18.81) | 123.17(95.66,164.9) | 9.45(2.82,18.46) | 95.86(71.75,134.12) | 2.78(-1.7,7.46) |
|  | Iraq | 1434.07(409.7,3032.4) | 437.01(303.02,624.52) | 3.4(0.97,7.2) | 124.35(68.37,202.69) | 7.05(2.02,14.52) | 91.4(45.02,155.02) | 2.32(1.84,2.8) |
|  | Tokelau | 0.11(0.03,0.24) | 73.39(34.81,130.43) | 7.87(2.21,17.14) | 107.37(61.22,175.59) | 8.65(2.43,18.74) | 82.85(43.93,141.07) | 2.13(-28.51,45.9) |
|  | Sri Lanka | 1090.65(322.72,2384.73) | 566.86(395.4,823.31) | 4.99(1.48,10.91) | 425.49(290.38,627.58) | 4.54(1.35,9.86) | 152.34(89.45,242.62) | 3.99(3.4,4.58) |
|  | China | 90654.64(23078.27,197161.13) | 264.27(198.53,353.14) | 6.37(1.62,13.86) | 203.14(148.44,277.1) | 4.7(1.2,10.21) | 44.16(18.35,76.05) | 1.32(1.21,1.43) |
|  | Georgia | 432.69(113.75,914.67) | 133.33(94.69,194.26) | 11.81(3.1,24.96) | 250.72(192.65,342.3) | 7.19(1.89,15.29) | 141.82(101.16,203.37) | 4.52(3.82,5.24) |
|  | Botswana | 79.86(21.32,173.38) | 422.31(277.28,638.13) | 3.41(0.91,7.41) | 190.71(109.99,310.83) | 7.12(1.95,15.22) | 121.96(62.06,207.57) | 2.82(0.96,4.71) |
|  | Thailand | 4390.36(1140.4,9980.97) | 342.85(233.54,511.46) | 6.26(1.63,14.24) | 259.25(170.57,396.03) | 4.42(1.15,10.01) | 36.65(3.21,86.89) | 0.03(-0.21,0.28) |
|  | Uruguay | 401.02(109.55,828.79) | 242.67(209.93,300.24) | 11.67(3.19,24.12) | 213.08(183.17,265.67) | 6.99(1.91,14.51) | 139.48(117.61,178) | 3.04(2.22,3.86) |
|  | South Africa | 2277.32(634.02,4600.44) | 232.49(183.39,293.23) | 4.1(1.14,8.28) | 120.29(87.75,160.53) | 5.65(1.59,11.34) | 53.21(30.38,81.2) | 1.59(1.3,1.87) |
|  | Saint Lucia | 16.88(5.12,34.11) | 210.84(161.36,277.03) | 9.67(2.93,19.53) | 144.58(105.65,196.66) | 8.06(2.46,16.28) | 23.03(3.81,48.67) | 0.26(-3.15,3.79) |
|  | Mexico | 5651.58(1612.17,11411.03) | 198.77(155.88,244.86) | 4.52(1.29,9.13) | 104.42(75.08,135.97) | 5.02(1.44,10.1) | 1.09(-13.48,16.55) | 0(-0.18,0.19) |
|  | Fiji | 63.9(19.48,133.18) | 294.21(192.96,471.13) | 7.01(2.14,14.61) | 228.49(144.12,375.91) | 9.62(2.93,19.68) | 83.51(40.25,159.97) | 2.07(0.08,4.1) |
|  | Jamaica | 236.71(67.02,500.34) | 274.51(203.81,376.81) | 8.42(2.38,17.8) | 214.94(155.49,300.97) | 7.93(2.23,16.76) | 127.49(83.67,191.42) | 2.86(1.86,3.86) |
|  | Indonesia | 8032.1(2038.3,17889.92) | 415.4(313.48,526.01) | 3.1(0.79,6.89) | 268.23(195.41,347.26) | 4.27(1.08,9.48) | 133.65(88.51,183.82) | 2.96(2.54,3.39) |
|  | Egypt | 2116.12(567.93,4855.02) | 577.17(389.97,848.6) | 2.14(0.57,4.9) | 280.71(175.47,433.31) | 3.58(0.97,8.1) | 204.42(122.48,323.87) | 4.24(3.8,4.68) |
|  | Paraguay | 227.1(59.18,507.68) | 568.45(395.99,791.14) | 3.28(0.85,7.33) | 290.2(189.53,420.18) | 4.34(1.14,9.62) | 162.97(96.67,248.51) | 3.24(2.02,4.47) |
|  | Saint Vincent and the Grenadines | 9.91(2.97,20.11) | 185.13(146.44,242.75) | 8.76(2.63,17.78) | 177.36(139.72,233.41) | 7.57(2.27,15.31) | 51.94(31.71,81.19) | 1.3(-3.06,5.87) |
|  | Algeria | 1235.88(353.06,2587.21) | 418.07(292.73,611.58) | 2.95(0.84,6.18) | 213(137.27,329.91) | 4.17(1.2,8.73) | 74.77(36.2,134.27) | 2.11(1.61,2.63) |
|  | Suriname | 45.85(13.24,93.7) | 397.6(317.03,521.72) | 7.96(2.3,16.27) | 234.05(179.96,317.38) | 7.95(2.29,16.23) | 109.98(76.64,160.96) | 2.91(0.44,5.44) |
|  | Panama | 185.62(50.91,403.9) | 331.69(237.38,464.43) | 4.46(1.22,9.71) | 147.83(93.68,224.03) | 4.49(1.23,9.77) | 47.57(15.28,92.68) | 1.31(0.12,2.5) |
|  | Turkmenistan | 78.73(20.68,171.96) | 208.13(147.23,293.15) | 1.55(0.41,3.38) | 124.61(80.21,186.58) | 2.12(0.56,4.59) | 56.57(27.2,96.5) | 1.84(-0.06,3.77) |
|  | Peru | 774.41(205.44,1798.47) | 349.2(238.41,516.76) | 2.28(0.6,5.29) | 187.13(116.31,294.23) | 2.45(0.65,5.68) | 53.22(15.18,109.58) | 1.36(0.77,1.95) |
|  | Mauritius | 128.23(39.03,263.87) | 431.93(326.42,600.11) | 10.04(3.06,20.67) | 358.35(267.43,503.27) | 7.57(2.31,15.48) | 108.81(68.74,172.96) | 2.81(1.26,4.37) |
|  | Albania | 138.22(32.82,327.11) | 254.05(162.85,373.86) | 5.08(1.21,12.02) | 330.78(219.82,476.55) | 3.14(0.75,7.39) | 51.99(12.63,103.31) | 1.25(-0.09,2.6) |
|  | Uzbekistan | 607.87(157.86,1291.77) | 355.04(267.71,488.12) | 1.81(0.47,3.84) | 183.04(128.72,265.82) | 3.48(0.93,7.36) | 190.09(139.7,269.56) | 4.56(3.79,5.33) |
|  | Brazil | 9533.96(2645.64,19517.78) | 215.93(196.26,242.37) | 4.4(1.22,9.01) | 117.03(103.51,135.19) | 4.16(1.15,8.47) | 9.74(3.37,18.12) | 0.52(0.37,0.67) |
|  | Tonga | 6.86(1.89,14.53) | 145.7(90.75,224.23) | 6.7(1.85,14.2) | 132.35(80.38,206.62) | 8.89(2.45,18.82) | 63.41(27.5,114.47) | 1.7(-3.15,6.8) |
|  | Ecuador | 573.52(157.46,1205.47) | 631.67(483.22,853.7) | 3.26(0.9,6.85) | 317.08(232.46,443.65) | 4.08(1.13,8.56) | 147.39(98.64,220.91) | 3.34(2.53,4.15) |
|  | Samoa | 8.84(2.56,19.04) | 175.72(116.2,264.88) | 4.18(1.21,9.01) | 113.47(67.39,182.5) | 6.37(1.85,13.57) | 62.79(29.95,113.03) | 1.52(-2.98,6.24) |
|  | Cuba | 1660(454.34,3496.77) | 164.11(116.03,230.57) | 14.61(4,30.79) | 151.87(106.03,215.25) | 8.47(2.31,17.91) | 37.64(12.57,72.25) | 1.34(1,1.69) |
|  | Equatorial Guinea | 21.48(5.41,50.67) | 467.02(259.33,784.61) | 1.51(0.38,3.57) | 71.86(8.91,168.12) | 5.45(1.41,12.65) | 153.15(64.97,294.2) | 3.83(-0.44,8.29) |
|  | Colombia | 2085.81(572.75,4630.77) | 214.63(143.21,301.89) | 4.37(1.2,9.69) | 114.34(65.68,173.78) | 3.93(1.08,8.71) | -8.2(-29.48,16.37) | -0.76(-1.06,-0.45) |
|  | Tunisia | 785.48(205.73,1769.02) | 379.64(241.55,607.59) | 6.79(1.78,15.29) | 249.81(149.1,416.05) | 6.54(1.72,14.66) | 80.2(29.41,161.65) | 2.05(1.47,2.62) |
|  | Republic of Moldova | 231.68(60.39,485.29) | 47.23(26.67,73.5) | 6.28(1.64,13.16) | 77.49(52.7,109.17) | 3.92(1.02,8.21) | 10.99(-4.27,29.97) | 1.32(0.5,2.15) |
| Low middle SDI | Maldives | 8.45(2.32,17.61) | 388.61(274.78,592.44) | 1.7(0.47,3.53) | 117.56(66.87,208.31) | 3.46(0.97,7.2) | 30.63(3.25,75.32) | -0.14(-5.7,5.75) |
|  | Philippines | 2871.48(757.7,6283.84) | 209.65(148.14,293.46) | 2.56(0.68,5.6) | 74.75(40.04,122.05) | 4.14(1.1,9) | 9.58(-10.74,37.67) | -0.23(-0.49,0.02) |
|  | Ghana | 560.87(151.84,1199.52) | 456.81(318.62,626.1) | 1.78(0.48,3.8) | 165.13(99.33,245.74) | 4.12(1.14,8.77) | 109.09(60.24,169.14) | 3.04(2.3,3.79) |
|  | Myanmar | 2304.3(617.83,5070.88) | 197.57(124.88,322.89) | 4.21(1.13,9.27) | 123.7(69.05,217.91) | 5.58(1.53,12.2) | 53.97(18.24,117.93) | 1.3(1,1.61) |
|  | Sudan | 586.05(164.79,1318.15) | 353.86(244.66,530.67) | 1.44(0.4,3.23) | 124.64(70.59,212.16) | 3.57(1,7.94) | 132.34(80.42,223.4) | 2.95(2.26,3.64) |
|  | Zambia | 159.88(41.6,350.64) | 251.46(176.24,355.42) | 0.88(0.23,1.92) | 53.07(20.31,98.35) | 2.91(0.77,6.34) | 49.9(21.01,88.69) | 1.27(0.1,2.46) |
|  | Democratic People's Republic of Korea | 1106.35(280.71,2435.16) | 238.37(167.17,326.75) | 4.22(1.07,9.28) | 171.58(114.43,242.51) | 3.5(0.89,7.75) | 57.16(25.6,96.2) | 1.65(1.18,2.11) |
|  | Dominican Republic | 268.61(69.32,612.45) | 578.65(392.52,821.63) | 2.47(0.64,5.63) | 349.22(226.02,510.06) | 3.04(0.79,6.94) | 151.4(83.37,241.05) | 4.21(3.02,5.42) |
|  | Guatemala | 417.7(120.61,902.16) | 693.13(521.06,968.01) | 2.35(0.68,5.08) | 255.44(178.33,378.63) | 4.03(1.17,8.66) | 129.68(81.51,205.95) | 2.87(1.94,3.81) |
|  | El Salvador | 229.65(63.82,511.44) | 474.23(333.09,662.54) | 3.67(1.02,8.18) | 383.49(264.65,542.04) | 3.82(1.06,8.51) | 167.73(101,256.9) | 3.32(2.16,4.5) |
|  | Honduras | 331.13(89.42,738.68) | 594.68(416.98,837.55) | 3.37(0.91,7.53) | 233.28(148.03,349.8) | 5.96(1.64,13.09) | 133.7(72.9,214.14) | 3.15(2.11,4.19) |
|  | Timor-Leste | 32.21(8.44,71.25) | 787.92(535.53,1124.34) | 2.41(0.63,5.34) | 420.82(272.78,618.16) | 4.39(1.15,9.62) | 157.02(84.44,255.49) | 3.35(-0.48,7.33) |
|  | Guyana | 41.81(12.05,89.85) | 190.57(122.26,285.27) | 5.43(1.56,11.66) | 190.33(122.08,284.95) | 7.16(2.07,15.1) | 71.1(33.25,123.83) | 1.9(-0.3,4.15) |
|  | Namibia | 44.96(11.8,98.54) | 286.15(196.81,418.54) | 1.87(0.49,4.1) | 126.52(74.11,204.18) | 3.53(0.93,7.61) | 98.28(53.71,161.19) | 2.6(0.31,4.93) |
|  | Kyrgyzstan | 81.88(21.06,177.72) | 44.83(24.27,71.57) | 1.25(0.32,2.72) | -1.12(-15.15,17.15) | 1.94(0.5,4.18) | 2.76(-11.52,22.13) | 0.39(-0.94,1.73) |
|  | Tajikistan | 141.22(37.48,305.18) | 220.72(154.99,327.59) | 1.49(0.39,3.22) | 81.63(44.4,142.15) | 3.44(0.93,7.3) | 114.38(72.36,183.34) | 3.83(2.45,5.23) |
|  | India | 30220.18(8319.79,62896.57) | 470.81(377.86,600.63) | 2.17(0.6,4.52) | 251.17(193.99,331.04) | 2.85(0.79,5.93) | 102.06(69.61,148.37) | 2.02(1.82,2.23) |
|  | Tuvalu | 0.91(0.25,1.97) | 185.8(115.58,295.09) | 7.68(2.14,16.72) | 126.4(70.78,212.97) | 9.38(2.62,20.26) | 87.47(43.66,156.04) | 2.13(-12.21,18.82) |
|  | Nicaragua | 172.89(49.16,354.68) | 508.17(383.51,681.5) | 2.66(0.76,5.45) | 263.12(188.69,366.61) | 4.41(1.26,9.01) | 110.01(66.84,168.91) | 2.54(1.18,3.9) |
|  | Viet Nam | 4694.08(1219.5,10093.23) | 438.74(318.85,604.14) | 4.87(1.27,10.47) | 279.81(195.29,396.41) | 5.67(1.51,12.11) | 147(93.51,223.83) | 3.27(3.01,3.53) |
|  | Kenya | 300.39(76.52,665.86) | 419.34(331.9,541.53) | 0.6(0.15,1.33) | 139.81(99.44,196.24) | 1.63(0.42,3.58) | 96.41(64.77,140.36) | 2.61(1.63,3.6) |
|  | Kiribati | 5.2(1.36,11.33) | 209.33(129.15,330.66) | 4.38(1.15,9.55) | 93.14(43.08,168.89) | 8.32(2.23,17.8) | 70.51(29.98,130.01) | 1.79(-5.67,9.85) |
|  | Nigeria | 1543.78(392.11,3374.14) | 240.39(152.92,341.27) | 0.72(0.18,1.57) | 42.9(6.18,85.25) | 2.2(0.57,4.77) | 78.94(34.55,129.53) | 2.08(1.7,2.46) |
|  | Lesotho | 59.37(15.12,134.66) | 244.13(132.99,394.33) | 2.84(0.72,6.44) | 197.34(101.31,327.12) | 5.34(1.38,11.88) | 167.07(80.75,278.99) | 4.36(2.46,6.29) |
|  | Palestine | 200.81(56.94,406.99) | 438.5(310.7,652.5) | 4.05(1.15,8.21) | 124.89(71.52,214.27) | 10.01(2.89,19.97) | 113.65(64.56,198.54) | 2.86(1.58,4.16) |
|  | Morocco | 1504.25(398.23,3276.43) | 465.62(329.53,649.66) | 4.18(1.11,9.11) | 297.98(202.23,427.47) | 5.03(1.34,10.88) | 143.48(87.67,221.87) | 3.04(2.56,3.53) |
|  | Mauritania | 43.07(10.88,93.73) | 218.78(148.32,317.85) | 1.07(0.27,2.33) | 64.1(27.82,115.09) | 2.36(0.6,5.11) | 56.03(23.03,102.3) | 1.25(-0.99,3.54) |
|  | Marshall Islands | 4.52(1.25,10.16) | 277.54(186.19,411.28) | 7.96(2.19,17.87) | 203.72(130.24,311.31) | 14.59(4.1,31.95) | 80.64(39.87,137.97) | 1.97(-5.86,10.46) |
|  | Venezuela (Bolivarian Republic of) | 1606.25(442.07,3599.8) | 427.56(301.78,599.54) | 5.72(1.57,12.82) | 253.91(169.53,369.28) | 5.71(1.58,12.76) | 64.84(25.43,116.99) | 1.84(1.41,2.27) |
|  | Mongolia | 51.73(12.56,116.63) | 212.54(141.93,317.37) | 1.53(0.37,3.44) | 98.7(53.81,165.35) | 2.78(0.68,6.19) | 63.12(28.74,114.21) | 1.6(-0.48,3.72) |
|  | Eswatini | 32.06(8.38,73.35) | 294.75(188.25,449.48) | 2.81(0.73,6.42) | 178.82(103.6,288.11) | 6.53(1.74,14.72) | 91.79(40.73,165.38) | 2.57(-0.05,5.26) |
|  | Micronesia (Federated States of) | 7.35(1.94,17.21) | 261.47(148.23,442.83) | 7.2(1.9,16.86) | 269.14(153.51,454.35) | 11.68(3.15,26.5) | 141.32(71.71,255.56) | 3.11(-2.77,9.34) |
|  | Lao People's Democratic Republic | 223.27(59.4,489.79) | 254.07(168.37,375.65) | 3.12(0.83,6.84) | 105.35(55.65,175.87) | 5.96(1.62,13) | 77.07(36.55,134.92) | 1.63(0.56,2.71) |
|  | Sao Tome and Principe | 4.28(1.12,9.26) | 225.78(158.56,314.37) | 2.08(0.55,4.51) | 92.77(53,145.19) | 4.98(1.32,10.76) | 113.85(71.05,167.3) | 2.76(-4.17,10.2) |
|  | Bolivia (Plurinational State of) | 346.08(88.08,783.22) | 421.38(312,570.64) | 2.88(0.73,6.52) | 178.71(120.24,258.5) | 4.32(1.11,9.75) | 83.89(46.07,135.59) | 1.85(0.93,2.77) |
|  | Cameroon | 365.02(93.24,812.86) | 451.82(321.41,636.13) | 1.25(0.32,2.79) | 97.03(50.46,162.84) | 3.8(1,8.39) | 106.45(60.06,171.19) | 2.56(1.65,3.48) |
|  | Congo | 105.55(27.01,235.94) | 211.24(139.9,309.69) | 2(0.51,4.48) | 44.5(11.38,90.21) | 4.84(1.27,10.45) | 32.56(4.47,69.64) | 0.84(-0.64,2.34) |
|  | Syrian Arab Republic | 380.96(101.4,828.45) | 351.36(224.12,546.15) | 2.63(0.7,5.72) | 301.64(188.41,474.97) | 3.5(0.96,7.44) | 92.94(40.2,172.01) | 2.03(1.22,2.85) |
|  | Belize | 10.05(2.79,20.97) | 526.89(433.59,660.77) | 2.45(0.68,5.11) | 184.15(141.86,244.84) | 3.95(1.1,8.2) | 120.2(86.97,167.48) | 2.72(-2.97,8.75) |
|  | Cabo Verde | 22.88(6.43,48.47) | 574.51(425.27,754.95) | 4.06(1.14,8.6) | 320.84(227.72,433.41) | 5.78(1.62,12.26) | 310.67(219.18,426.1) | 4.29(0.02,8.73) |
|  | Nauru | 0.41(0.1,0.94) | 93.72(49.03,161.06) | 3.88(0.99,8.88) | 88.28(44.84,153.72) | 12.04(3.2,26.25) | 69.64(34.69,123.19) | 1.72(-19.12,27.93) |
| Low SDI | Vanuatu | 11.58(2.95,25.73) | 479.28(326.98,775.51) | 3.93(1,8.74) | 197.72(119.45,349.97) | 7.31(1.88,16.1) | 106.97(53.91,210.02) | 2.53(-2.86,8.22) |
|  | Yemen | 286.39(73.65,644.46) | 438.9(313.65,644.16) | 0.91(0.23,2.05) | 134.86(80.27,224.31) | 2.47(0.65,5.43) | 97.66(55.87,169.88) | 2.74(1.64,3.85) |
|  | Uganda | 374.66(99.72,805.51) | 342.05(247.47,478.63) | 0.91(0.24,1.96) | 86.15(46.32,143.67) | 3.14(0.85,6.72) | 104.57(63.77,161.97) | 2.51(1.68,3.35) |
|  | Gambia | 20.02(5.19,43.46) | 585.78(403.36,835.2) | 0.89(0.23,1.94) | 202.89(122.32,313.06) | 2.36(0.62,5.11) | 133.43(74.62,212.2) | 3.02(-1.13,7.35) |
|  | Afghanistan | 510.46(139.58,1146.29) | 191.9(119.47,290.77) | 1.33(0.36,2.99) | -12.92(-34.53,16.57) | 4.8(1.36,10.44) | 82.19(39.88,137.81) | 2.31(1.65,2.98) |
|  | Djibouti | 12.41(3.12,29.32) | 710.43(505.48,993.99) | 1.03(0.26,2.44) | 227.47(144.65,342.04) | 2.68(0.7,6.09) | 75.61(36.39,126.54) | 2.04(-3.84,8.29) |
|  | Guinea | 138.25(36.31,301.51) | 217.3(147.95,308.46) | 1.09(0.29,2.38) | 55.26(21.32,99.86) | 2.85(0.75,6.2) | 92.65(50.89,147.25) | 2.49(1.29,3.7) |
|  | Burundi | 62.54(15.41,146.76) | 93.89(38.12,168.39) | 0.52(0.13,1.23) | -9.49(-35.53,25.28) | 1.67(0.42,3.87) | 8.75(-20.85,47.5) | -0.02(-1.57,1.56) |
|  | Bangladesh | 2240.2(545.16,5302.39) | 321.97(216,461.04) | 1.41(0.34,3.33) | 188.95(116.39,284.17) | 1.81(0.44,4.29) | 45.91(10.71,91.53) | 1.3(0.94,1.65) |
|  | Bhutan | 12.24(3.17,26.82) | 470.93(324.83,696.62) | 1.62(0.42,3.56) | 363.38(244.81,546.56) | 2.38(0.62,5.21) | 134.07(76.43,217.51) | 3.05(-2.2,8.57) |
|  | Eritrea | 52.35(12.47,115.26) | 453.55(304.6,678.9) | 0.78(0.19,1.72) | 147.56(80.95,248.35) | 2.41(0.61,5.26) | 101.88(48.69,181.48) | 2.29(-0.48,5.13) |
|  | Cambodia | 588.31(155.99,1251.01) | 670.43(499.53,903.14) | 3.54(0.94,7.53) | 381.45(274.65,526.87) | 5.64(1.52,11.74) | 185.18(123.61,271.64) | 3.64(2.76,4.52) |
|  | Comoros | 8.49(2.22,18.38) | 228.18(147.12,385.06) | 1.19(0.31,2.57) | 113.97(61.12,216.25) | 1.91(0.5,4.1) | 45.62(11.09,106.4) | 1.3(-3.5,6.34) |
|  | Guinea-Bissau | 19.79(5.04,45.16) | 180.02(110.53,279.66) | 1.04(0.27,2.38) | 48.36(11.55,101.16) | 3.35(0.86,7.51) | 65.67(27.49,120.01) | 1.82(-1.41,5.15) |
|  | Liberia | 54.48(14.06,120.43) | 156.53(94.91,249.27) | 1.14(0.29,2.51) | 5.2(-20.07,43.23) | 3.32(0.87,7.26) | 55.06(18.89,106.42) | 2.18(0.3,4.09) |
|  | Madagascar | 138.34(34.57,330.76) | 175.38(101.54,261.09) | 0.52(0.13,1.24) | 23.31(-9.76,61.69) | 1.56(0.4,3.65) | 36.83(2.7,77.04) | 1.08(-0.1,2.27) |
|  | Mali | 202(54.61,436.76) | 267.51(190.97,364.84) | 0.92(0.25,1.99) | 45.43(15.14,83.94) | 2.79(0.77,6.01) | 75(39.53,118.06) | 1.96(0.89,3.04) |
|  | Haiti | 358.59(96.9,814.09) | 226.47(142.54,346.25) | 2.89(0.78,6.56) | 67.31(24.3,128.69) | 5.68(1.52,12.78) | 52.27(16.59,104.34) | 1.59(0.77,2.41) |
|  | Pakistan | 6003.44(1555.19,12977.02) | 333.03(236.28,494.52) | 2.68(0.69,5.79) | 118.07(69.35,199.4) | 5.97(1.56,12.81) | 131.71(81.57,215.45) | 3.13(2.91,3.34) |
|  | Ethiopia | 493.59(121.1,1115.93) | 112.9(67.96,191.75) | 0.46(0.11,1.04) | 1.69(-19.78,39.36) | 1.41(0.35,3.17) | 1.73(-18.24,32.25) | -0.69(-1.33,-0.05) |
|  | Zimbabwe | 392.43(107.63,835.2) | 237.98(161.13,347.93) | 2.61(0.72,5.56) | 132.77(79.84,208.49) | 6.6(1.83,13.93) | 88.9(48.38,147.13) | 2.77(2.03,3.51) |
|  | Mozambique | 231.84(60.03,515.06) | 296.23(195.04,433.02) | 0.79(0.2,1.74) | 75.4(30.6,135.95) | 2.59(0.69,5.67) | 107.76(55.08,177.79) | 3.2(2.16,4.26) |
|  | Angola | 325.22(82.71,722.51) | 320.38(216.06,485.16) | 1.08(0.27,2.4) | 43.92(8.21,100.34) | 3.56(0.95,7.77) | 48.32(16.36,100.8) | 1.46(0.47,2.46) |
|  | South Sudan | 64.35(16.14,147.01) | 96.86(45.15,165.66) | 0.69(0.17,1.58) | 24.26(-8.38,67.69) | 2.07(0.52,4.7) | 29.32(-2.04,71.32) | 0.99(-0.56,2.56) |
|  | Nepal | 513.62(128.29,1128.18) | 516.75(343.61,748.21) | 1.69(0.42,3.71) | 296.14(184.93,444.81) | 2.49(0.63,5.41) | 148.96(78.38,248.19) | 3.13(2.26,4) |
|  | Burkina Faso | 204.25(54.41,439.05) | 237.99(175.79,320.78) | 0.9(0.24,1.93) | 42.36(16.16,77.23) | 2.73(0.73,5.77) | 62.28(33.34,101.43) | 1.82(0.77,2.89) |
|  | Niger | 88.73(21.94,201.67) | 522.4(389.38,697.3) | 0.38(0.09,0.87) | 114.36(68.54,174.59) | 1.45(0.36,3.29) | 114.61(71.91,168.87) | 2.72(0.7,4.79) |
|  | Democratic Republic of the Congo | 922.28(213.78,2246.36) | 188.48(109.66,293.25) | 1.05(0.24,2.56) | 26.98(-7.71,73.1) | 3.08(0.73,7.38) | 22.31(-8.3,63.39) | 0.56(0.06,1.05) |
|  | Malawi | 154.33(41.11,327.32) | 203.98(147.12,280.16) | 0.84(0.22,1.77) | 57.5(28.04,96.97) | 2.54(0.68,5.38) | 54.4(26.01,91.19) | 1.66(0.51,2.84) |
|  | Papua New Guinea | 290.66(74.94,655.13) | 365.24(253.37,528.26) | 2.95(0.76,6.64) | 92.74(46.39,160.27) | 6.45(1.66,14.13) | 77.71(38.57,133.57) | 2.02(0.82,3.24) |
|  | Central African Republic | 57.35(13.44,147.73) | 128.06(73.04,195.16) | 1.08(0.25,2.79) | 18.08(-10.41,52.82) | 3.06(0.77,7.48) | 23.81(-3.71,56.77) | 0.82(-1.21,2.9) |
|  | Benin | 121.42(32.7,260.65) | 310.8(227.34,431.96) | 0.96(0.26,2.06) | 57.39(25.42,103.81) | 3(0.82,6.4) | 81.22(46.17,131.88) | 2.12(0.68,3.58) |
|  | Rwanda | 108.41(27.04,241.4) | 153.43(91.7,247.45) | 0.85(0.21,1.9) | 43.27(8.37,96.42) | 2.19(0.56,4.83) | 27.45(-0.29,68.62) | 0.3(-1.04,1.66) |
|  | Senegal | 275.48(73.6,597.13) | 293.78(212.33,417.56) | 1.82(0.49,3.95) | 98.29(57.27,160.61) | 4.25(1.16,9.15) | 67.98(35.95,117.43) | 2.16(1.22,3.12) |
|  | Solomon Islands | 31.59(7.78,71.53) | 607.31(408.43,958.78) | 4.82(1.19,10.91) | 267.28(164.01,449.78) | 10.35(2.63,23.18) | 186.53(114.52,309.55) | 3.57(-1.2,8.56) |
|  | Togo | 63.54(15.84,141.4) | 399.96(281.81,549.19) | 0.8(0.2,1.78) | 131.18(76.55,200.19) | 2.22(0.56,4.86) | 72.37(34.87,119.26) | 1.84(-0.26,3.98) |
|  | C么te d'Ivoire | 272.15(71.97,593.6) | 389.2(290.91,533.84) | 1.04(0.27,2.27) | 128.54(82.62,196.11) | 3.36(0.91,7.25) | 74.48(41.94,120.13) | 1.75(0.75,2.75) |
|  | Sierra Leone | 54.99(13.58,121.7) | 255.25(170.94,378.89) | 0.66(0.16,1.47) | 56.6(19.43,111.11) | 1.81(0.45,4) | 101.12(53,169.06) | 3(0.83,5.21) |
|  | United Republic of Tanzania | 438.85(113.41,955.07) | 350.77(269.72,445.99) | 0.77(0.2,1.68) | 105.79(68.79,149.26) | 2.09(0.55,4.57) | 89.27(58.47,126.06) | 3.04(2.25,3.83) |
|  | Somalia | 79.9(18.26,205.42) | 252.9(144.44,393.72) | 0.39(0.09,1.01) | 23.99(-14.12,73.47) | 1.46(0.34,3.75) | 28.64(-7.89,75.24) | 1.09(-0.7,2.91) |
|  | Chad | 119.36(29.62,263.31) | 260.72(188.17,361.33) | 0.73(0.18,1.61) | 32.46(5.82,69.41) | 2.56(0.65,5.62) | 98.65(59.97,152.32) | 2.5(1.12,3.9) |

# Table S8. Trends in cancer mortality attributable to HPFG for both females and males in representative countries, 1990-2019.

| **Country** | **Gender** | **Deaths** | | **All-age mortality** | | **Age-standardized mortality** | | **Net drift of mortality from APC model,% per year** |
| --- | --- | --- | --- | --- | --- | --- | --- | --- |
|  |  | **Number in 2019** | **Percent change 1990-2019, %** | **Rate in 2019** | **Percent change 1990-2019, %** | **Rate in 2019** | **Percent change 1990-2019, %** |  |
| United States of America | Both | 58134.43(16746.97,114099.58) | 104.62(92.23,127.61) | 17.73(5.11,34.79) | 58.22(48.64,76) | 9.97(2.86,19.61) | 14.61(7.63,27.31) | 1.19(1.1,1.29) |
|  | Female | 27937.56(7797.24,56879.44) | 103.07(90.7,127.6) | 16.76(4.68,34.12) | 58.12(48.49,77.22) | 8.64(2.41,17.56) | 19.26(11.94,33.08) | 1.47(1.36,1.58) |
|  | Male | 30196.87(7921.98,61175.18) | 106.07(90.31,138.81) | 18.72(4.91,37.93) | 58.19(46.09,83.32) | 11.66(3.05,23.63) | 7.85(-0.38,24.62) | 0.84(0.74,0.93) |
| United Kingdom | Both | 13805.92(4055.56,27142.98) | 79.13(65.13,104.97) | 20.54(6.03,40.38) | 53.15(41.19,75.24) | 10.15(2.95,20.01) | 25.23(16.02,42.51) | 0.49(0.37,0.61) |
|  | Female | 7337.17(2065.5,14823.6) | 93.92(77.18,122.08) | 21.6(6.08,43.65) | 68.94(54.36,93.47) | 9.69(2.74,19.68) | 44.99(33.54,65.79) | 0.86(0.69,1.04) |
|  | Male | 6468.74(1695.21,13103.88) | 64.86(51.68,92.93) | 19.45(5.1,39.4) | 38.23(27.17,61.76) | 10.71(2.8,21.69) | 3.57(-4.54,20.02) | 0.03(-0.14,0.2) |
| Italy | Both | 11865.73(3407.66,23645.51) | 90.68(74.69,111.66) | 19.67(5.65,39.2) | 79.57(64.51,99.32) | 7.5(2.14,14.91) | 10.76(3.23,22.06) | 0.33(0.2,0.46) |
|  | Female | 5232.58(1440.61,10716.55) | 97.58(77.87,119.2) | 16.89(4.65,34.6) | 86.48(67.88,106.88) | 5.68(1.57,11.61) | 16.4(7.36,28.66) | 0.56(0.36,0.77) |
|  | Male | 6633.15(1687.79,13589.66) | 85.57(69.72,113.49) | 22.61(5.75,46.32) | 74.35(59.45,100.57) | 9.86(2.47,20.2) | 4.94(-2.87,18.93) | 0.11(-0.06,0.27) |
| China | Both | 90654.64(23078.27,197161.13) | 264.27(198.53,353.14) | 6.37(1.62,13.86) | 203.14(148.44,277.1) | 4.7(1.2,10.21) | 44.16(18.35,76.05) | 1.32(1.21,1.43) |
|  | Female | 33934.58(8193.97,77063.47) | 216.59(145.1,311.11) | 4.86(1.17,11.05) | 160.29(101.52,238) | 3.3(0.8,7.48) | 24.63(-3.22,60.61) | 0.74(0.6,0.88) |
|  | Male | 56720.06(12509.93,129336.01) | 300.34(195.32,438.94) | 7.83(1.73,17.84) | 237.02(148.62,353.7) | 6.57(1.48,14.91) | 58.45(20.32,107.13) | 1.65(1.52,1.78) |
| India | Both | 30220.18(8319.79,62896.57) | 470.81(377.86,600.63) | 2.17(0.6,4.52) | 251.17(193.99,331.04) | 2.85(0.79,5.93) | 102.06(69.61,148.37) | 2.02(1.82,2.23) |
|  | Female | 16776.41(4417.66,36498.3) | 545.95(406.08,753.99) | 2.48(0.65,5.39) | 291.14(206.44,417.1) | 3.03(0.8,6.59) | 118.08(72.02,186) | 2.34(2.09,2.59) |
|  | Male | 13443.77(3353.24,29505.29) | 398.45(284.3,558.73) | 1.89(0.47,4.14) | 211.25(139.97,311.33) | 2.67(0.67,5.83) | 85.49(44.81,145.93) | 1.67(1.47,1.87) |
| Pakistan | Both | 6003.44(1555.19,12977.02) | 333.03(236.28,494.52) | 2.68(0.69,5.79) | 118.07(69.35,199.4) | 5.97(1.56,12.81) | 131.71(81.57,215.45) | 3.13(2.91,3.34) |
|  | Female | 3586.87(858.39,8358.43) | 472.56(294.26,808.76) | 3.28(0.79,7.65) | 181.6(93.91,346.95) | 7.14(1.74,16.35) | 182.22(96.3,348.31) | 3.96(3.64,4.28) |
|  | Male | 2416.56(533.95,5607.73) | 218(129.3,342.22) | 2.1(0.46,4.88) | 63.7(18.04,127.65) | 4.86(1.09,11.06) | 88(37.62,159.72) | 2.31(2.01,2.6) |

# Table S9. Trends in tracheal, bronchus, and lung cancer mortality attributable to HPFG for both females and males in representative countries, 1990-2019.

| **Country** | **Gender** | **Deaths** | | **All-age mortality** | | **Age-standardized mortality** | | **Net drift of mortality from APC model,% per year** |
| --- | --- | --- | --- | --- | --- | --- | --- | --- |
|  |  | **Number in 2019** | **Percent change 1990-2019, %** | **Rate in 2019** | **Percent change 1990-2019, %** | **Rate in 2019** | **Percent change 1990-2019, %** |  |
| United States of America | Both | 27719.29(6895.63,57335.3) | 97.68(83.55,126.16) | 8.45(2.1,17.48) | 52.85(41.93,74.88) | 4.77(1.19,9.89) | 10.17(2.23,26.08) | 1.02(0.91,1.14) |
|  | Female | 10896.95(2253.71,24720.54) | 138.27(119.01,168.81) | 6.54(1.35,14.83) | 85.52(70.53,109.31) | 3.4(0.71,7.72) | 37.82(26.58,55.82) | 1.95(1.81,2.09) |
|  | Male | 16822.34(3053.46,36256.98) | 78.03(65.65,98.95) | 10.43(1.89,22.48) | 36.67(27.16,52.73) | 6.46(1.17,13.94) | -6.4(-12.86,4.75) | 0.29(0.17,0.41) |
| United Kingdom | Both | 5593.34(1407.51,11681.66) | 67.15(49.96,105.85) | 8.32(2.09,17.38) | 42.92(28.22,76) | 4.16(1.04,8.72) | 19.2(7.73,45.85) | 0.51(0.33,0.7) |
|  | Female | 2396.84(497.45,5444.69) | 142.63(122.31,176.08) | 7.06(1.46,16.03) | 111.38(93.67,140.52) | 3.24(0.67,7.32) | 83.26(68.07,108.23) | 1.92(1.6,2.25) |
|  | Male | 3196.5(569.35,7042.12) | 35.54(25.88,51.07) | 9.61(1.71,21.17) | 13.64(5.54,26.66) | 5.28(0.94,11.66) | -13.16(-19.2,-3.53) | -0.5(-0.73,-0.27) |
| Italy | Both | 4304.25(958.73,9244.9) | 73.44(57.96,103.78) | 7.14(1.59,15.33) | 63.33(48.75,91.9) | 2.84(0.63,6.1) | 5.6(-3.09,22.26) | 0.28(0.08,0.48) |
|  | Female | 1050.6(205.25,2400.36) | 162.81(138.51,189.82) | 3.39(0.66,7.75) | 148.04(125.11,173.54) | 1.22(0.24,2.8) | 66.86(53.42,82.45) | 2.28(1.81,2.76) |
|  | Male | 3253.65(572.87,7263.69) | 56.28(44.25,73.79) | 11.09(1.95,24.76) | 46.83(35.53,63.28) | 4.89(0.85,10.9) | -8.48(-14.85,1.24) | -0.34(-0.56,-0.11) |
| China | Both | 53004.92(11784.92,121264.97) | 267.41(190.8,368.33) | 3.73(0.83,8.53) | 205.76(142.01,289.74) | 2.73(0.61,6.24) | 47.57(17.96,86.29) | 1.42(1.31,1.53) |
|  | Female | 15785.2(2935.11,38934.4) | 242.08(167.72,343.88) | 2.26(0.42,5.58) | 181.25(120.11,264.95) | 1.54(0.29,3.79) | 35.4(6.24,74.56) | 0.94(0.81,1.06) |
|  | Male | 37219.71(6193.73,91103.8) | 279.32(172.27,417.55) | 5.14(0.85,12.57) | 219.33(129.21,335.7) | 4.22(0.7,10.21) | 52.96(12.21,103.78) | 1.57(1.43,1.7) |
| India | Both | 8861.51(1980.64,20244.6) | 390.15(260.51,548.54) | 0.64(0.14,1.46) | 201.55(121.79,298.99) | 0.82(0.19,1.86) | 74.28(27.65,128.2) | 1.43(1.23,1.63) |
|  | Female | 2394.61(479.96,5813.08) | 662.86(488.38,901.79) | 0.35(0.07,0.86) | 361.92(256.28,506.6) | 0.43(0.09,1.05) | 149.93(91.74,228.39) | 2.87(2.43,3.32) |
|  | Male | 6466.91(1163.49,15475.88) | 332.86(206.83,482.89) | 0.91(0.16,2.17) | 170.29(91.59,263.97) | 1.25(0.22,2.96) | 64.11(15.2,119.37) | 1.21(0.99,1.44) |
| Pakistan | Both | 1638.53(327.71,4057.13) | 230.23(137.39,372.43) | 0.73(0.15,1.81) | 66.3(19.55,137.91) | 1.62(0.33,3.96) | 76.23(27.67,151.91) | 2.04(1.67,2.41) |
|  | Female | 257.78(50.32,636.69) | 538.73(322.18,853.58) | 0.24(0.05,0.58) | 214.14(107.64,369) | 0.54(0.11,1.35) | 219.02(113.12,377.86) | 4.41(3.22,5.61) |
|  | Male | 1380.76(233.33,3569.07) | 202.91(111.6,332.13) | 1.2(0.2,3.11) | 55.94(8.93,122.46) | 2.64(0.45,6.68) | 74.17(23.6,148.05) | 1.99(1.6,2.38) |

# Table S10. Trends in colon and rectal cancer mortality attributable to HPFG for both females and males in representative countries, 1990-2019.

| **Country** | **Gender** | **Deaths** | | **All-age mortality** | | **Age-standardized mortality** | | **Net drift of mortality from APC model,% per year** |
| --- | --- | --- | --- | --- | --- | --- | --- | --- |
|  |  | **Number in 2019** | **Percent change 1990-2019, %** | **Rate in 2019** | **Percent change 1990-2019, %** | **Rate in 2019** | **Percent change 1990-2019, %** |  |
| United States of America | Both | 10990.96(2766.37,23061.01) | 85.64(75.25,106.73) | 3.35(0.84,7.03) | 43.55(35.51,59.85) | 1.87(0.47,3.92) | 5.4(-0.66,17.3) | 0.89(0.75,1.02) |
|  | Female | 4680.6(916.48,10550.83) | 64.53(55.1,79.15) | 2.81(0.55,6.33) | 28.11(20.76,39.49) | 1.39(0.27,3.14) | -0.92(-6.38,7.9) | 0.74(0.53,0.95) |
|  | Male | 6310.35(1226.06,13610.39) | 105.17(91.43,126.58) | 3.91(0.76,8.44) | 57.5(46.95,73.94) | 2.46(0.48,5.31) | 6.46(-0.44,17.54) | 0.85(0.67,1.02) |
| United Kingdom | Both | 3220.07(834.83,6757.5) | 80.28(66.01,101.83) | 4.79(1.24,10.05) | 54.14(41.94,72.57) | 2.32(0.6,4.9) | 24.36(14.6,39.84) | 0.34(0.07,0.61) |
|  | Female | 1451.06(287.2,3200.3) | 64.31(51.16,84.56) | 4.27(0.85,9.42) | 43.15(31.69,60.79) | 1.8(0.35,4.01) | 22.24(12.67,36.75) | 0.09(-0.35,0.53) |
|  | Male | 1769.01(342.24,3830.02) | 95.9(80.6,118.73) | 5.32(1.03,11.52) | 64.25(51.43,83.4) | 2.94(0.57,6.39) | 19.85(10.98,33.6) | 0.36(0.02,0.7) |
| Italy | Both | 2935.34(735.92,6313.18) | 104.92(88.22,128.4) | 4.87(1.22,10.47) | 92.98(77.25,115.09) | 1.78(0.44,3.85) | 14.59(5.62,28.11) | 0.53(0.25,0.81) |
|  | Female | 1282.39(254.03,2902.5) | 80.1(60.53,99.08) | 4.14(0.82,9.37) | 69.99(51.51,87.9) | 1.28(0.25,2.89) | 0.47(-7.92,10.01) | -0.1(-0.57,0.37) |
|  | Male | 1652.95(308.54,3664.7) | 129.44(112.48,155.13) | 5.63(1.05,12.49) | 115.56(99.63,139.7) | 2.43(0.45,5.39) | 23.83(15.64,37.31) | 0.84(0.48,1.21) |
| China | Both | 18160.56(4029.89,41451.5) | 310.58(231.52,410.29) | 1.28(0.28,2.91) | 241.69(175.89,324.66) | 0.97(0.21,2.21) | 56.56(26.8,93.1) | 1.83(1.71,1.95) |
|  | Female | 6564.65(1166.78,15771.16) | 202.38(139.46,289.87) | 0.94(0.17,2.26) | 148.61(96.88,220.54) | 0.65(0.12,1.56) | 15.76(-8.44,48.04) | 0.61(0.43,0.78) |
|  | Male | 11595.91(2040.06,28074.77) | 414.88(294.93,573.32) | 1.6(0.28,3.87) | 333.45(232.47,466.83) | 1.39(0.25,3.38) | 96.43(54.65,149.64) | 2.64(2.48,2.8) |
| India | Both | 7748.92(1876.69,17094.69) | 516.17(388.18,669.16) | 0.56(0.13,1.23) | 279.08(200.33,373.2) | 0.76(0.19,1.67) | 112(68.82,163.88) | 2.13(1.91,2.35) |
|  | Female | 3822.05(730.57,8990.13) | 573.13(420.81,805.59) | 0.56(0.11,1.33) | 307.59(215.36,448.35) | 0.72(0.14,1.71) | 114.65(65.78,188.43) | 2.18(1.86,2.49) |
|  | Male | 3926.87(729.5,9167.58) | 469.28(297.32,661.45) | 0.55(0.1,1.29) | 255.47(148.1,375.47) | 0.8(0.15,1.86) | 110.43(47.51,180.65) | 2.1(1.79,2.4) |
| Pakistan | Both | 751.08(170.17,1728.19) | 326.81(224.28,480.05) | 0.34(0.08,0.77) | 114.94(63.31,192.11) | 0.8(0.19,1.88) | 136.14(79.98,223.82) | 3.22(2.64,3.8) |
|  | Female | 311.33(55.55,749.87) | 387.97(256.46,622.51) | 0.29(0.05,0.69) | 140(75.32,255.35) | 0.7(0.13,1.67) | 148.96(81.78,270.21) | 3.5(2.55,4.47) |
|  | Male | 439.75(73.74,1099.73) | 292.02(170.38,479.36) | 0.38(0.06,0.96) | 101.81(39.19,198.24) | 0.91(0.15,2.28) | 133.2(63.39,241.89) | 3.12(2.39,3.86) |

# Table S11. Trends in breast cancer mortality attributable to HPFG for both females in representative countries, 1990-2019.

| **Country** | **Gender** | **Deaths** | | **All-age mortality** | | **Age-standardized mortality** | | **Net drift of mortality from APC model,% per year** |
| --- | --- | --- | --- | --- | --- | --- | --- | --- |
|  |  | **Number in 2019** | **Percent change 1990-2019, %** | **Rate in 2019** | **Percent change 1990-2019, %** | **Rate in 2019** | **Percent change 1990-2019, %** |  |
| United States of America | Both | 5865.44(1162.22,12513.96) | 65.58(55.52,81.11) | 1.79(0.35,3.82) | 28.03(20.25,40.04) | 1.02(0.2,2.18) | -8.26(-13.77,0.13) | 0.56(0.39,0.72) |
|  | Female | 5865.44(1162.22,12513.96) | 65.58(55.52,81.11) | 3.52(0.7,7.51) | 28.92(21.09,41.02) | 1.85(0.36,3.97) | -4.35(-10.1,4.47) | 0.79(0.62,0.96) |
| United Kingdom | Both | 1731.55(360.36,3775.71) | 57.43(44.97,77.2) | 2.58(0.54,5.62) | 34.61(23.95,51.51) | 1.29(0.27,2.83) | 7.31(-0.32,20.1) | -0.38(-0.69,-0.06) |
|  | Female | 1731.55(360.36,3775.71) | 57.43(44.97,77.2) | 5.1(1.06,11.12) | 37.16(26.3,54.38) | 2.34(0.48,5.12) | 15.91(7.83,29.73) | -0.09(-0.4,0.23) |
| Italy | Both | 1405.91(287.29,3078.19) | 67.56(50.81,84.64) | 2.33(0.48,5.1) | 57.8(42.02,73.88) | 0.89(0.18,1.95) | -5.28(-12.24,3.18) | -0.37(-0.71,-0.03) |
|  | Female | 1405.91(287.29,3078.19) | 67.56(50.81,84.64) | 4.54(0.93,9.94) | 58.15(42.34,74.27) | 1.59(0.32,3.46) | -1.71(-8.78,6.92) | -0.18(-0.52,0.17) |
| China | Both | 5453.19(1002.85,12881.76) | 182.39(117.09,271.44) | 0.38(0.07,0.91) | 135.01(80.66,209.11) | 0.28(0.05,0.65) | 14.88(-11.01,49.56) | 0.59(0.39,0.79) |
|  | Female | 5453.19(1002.85,12881.76) | 182.39(117.09,271.44) | 0.78(0.14,1.85) | 132.18(78.49,205.39) | 0.52(0.1,1.23) | 14.49(-11.45,49.78) | 0.65(0.45,0.85) |
| India | Both | 6349.17(1188.39,14888.16) | 439.99(313.59,623.31) | 0.46(0.09,1.07) | 232.21(154.45,344.99) | 0.57(0.11,1.34) | 104.89(56.6,173.57) | 2.07(1.79,2.34) |
|  | Female | 6349.17(1188.39,14888.16) | 439.99(313.59,623.31) | 0.94(0.18,2.2) | 226.98(150.44,337.98) | 1.1(0.21,2.59) | 92.31(46.98,157.22) | 1.8(1.52,2.07) |
| Pakistan | Both | 2210.86(437.52,5456.34) | 449.93(260.48,876.02) | 0.99(0.2,2.44) | 176.94(81.53,391.52) | 2.06(0.42,5.01) | 190.58(95.4,419.65) | 4.14(3.72,4.57) |
|  | Female | 2210.86(437.52,5456.34) | 449.93(260.48,876.02) | 2.02(0.4,5) | 170.47(77.29,380.03) | 4.24(0.86,10.32) | 168.82(80.3,381.46) | 3.79(3.37,4.21) |

# Table S12. Trends in pancreatic cancer mortality attributable to HPFG for both females and males in representative countries, 1990-2019.

| **Country** | **Gender** | **Deaths** | | **All-age mortality** | | **Age-standardized mortality** | | **Net drift of mortality from APC model,% per year** |
| --- | --- | --- | --- | --- | --- | --- | --- | --- |
|  |  | **Number in 2019** | **Percent change 1990-2019, %** | **Rate in 2019** | **Percent change 1990-2019, %** | **Rate in 2019** | **Percent change 1990-2019, %** |  |
| United States of America | Both | 7553.95(1842.52,15702.37) | 197.2(177.58,233.36) | 2.3(0.56,4.79) | 129.81(114.63,157.77) | 1.3(0.31,2.7) | 68.78(57.55,89.14) | 2.88(2.7,3.07) |
|  | Female | 3305.58(586.37,7445.76) | 162.11(143.25,189.61) | 1.98(0.35,4.47) | 104.09(89.41,125.5) | 1.01(0.18,2.27) | 59.4(47.98,75.93) | 2.75(2.47,3.04) |
|  | Male | 4248.37(852.94,9318.63) | 231.76(207.15,269.64) | 2.63(0.53,5.78) | 154.68(135.78,183.76) | 1.64(0.33,3.6) | 72.99(60.26,92.83) | 2.89(2.64,3.14) |
| United Kingdom | Both | 1562.86(389.67,3253.19) | 156.49(137.51,187.94) | 2.32(0.58,4.84) | 119.29(103.07,146.19) | 1.15(0.29,2.41) | 81.58(68.59,103.77) | 2.11(1.71,2.52) |
|  | Female | 776.23(135.96,1733.21) | 146.84(125.33,182.45) | 2.29(0.4,5.1) | 115.04(96.31,146.07) | 1.01(0.18,2.26) | 89.82(74.06,116.2) | 2.05(1.42,2.69) |
|  | Male | 786.63(152.78,1741.76) | 166.78(145.72,197.79) | 2.37(0.46,5.24) | 123.68(106.02,149.68) | 1.31(0.25,2.92) | 69.35(56.27,88.77) | 2.07(1.54,2.61) |
| Italy | Both | 1638.49(400.13,3471.22) | 155.22(132.31,181.42) | 2.72(0.66,5.76) | 140.35(118.77,165.02) | 1.04(0.25,2.2) | 50(38.27,67.41) | 1.68(1.3,2.06) |
|  | Female | 819.66(142.98,1886.34) | 145.46(115.46,172.9) | 2.65(0.46,6.09) | 131.67(103.36,157.57) | 0.86(0.15,1.98) | 44.86(31.02,60.15) | 1.43(0.8,2.06) |
|  | Male | 818.83(156.36,1859.22) | 165.81(145.79,195.55) | 2.79(0.53,6.34) | 149.73(130.92,177.67) | 1.24(0.24,2.83) | 53.15(42.58,69.68) | 1.86(1.36,2.36) |
| China | Both | 8045.62(1759.35,18319.85) | 428.22(329.22,556.14) | 0.57(0.12,1.29) | 339.58(257.2,446.04) | 0.41(0.09,0.94) | 107.12(70.03,157.56) | 2.66(2.47,2.86) |
|  | Female | 3200.33(495.1,7859.15) | 376.08(271.46,512.3) | 0.46(0.07,1.13) | 291.42(205.41,403.42) | 0.31(0.05,0.76) | 85.55(45.49,137.09) | 2.16(1.87,2.46) |
|  | Male | 4845.29(848.44,11868.96) | 469.41(308.94,687.68) | 0.67(0.12,1.64) | 379.35(244.27,563.1) | 0.54(0.1,1.32) | 123.63(65.07,200.49) | 2.96(2.7,3.22) |
| India | Both | 3384.64(784.52,7427.04) | 797.91(600.39,1080.85) | 0.24(0.06,0.53) | 452.41(330.89,626.48) | 0.32(0.07,0.71) | 210.16(145.4,307.41) | 3.76(3.36,4.17) |
|  | Female | 1651.93(278.99,3891.79) | 1051.45(778.92,1443.65) | 0.24(0.04,0.57) | 597.22(432.2,834.71) | 0.3(0.05,0.71) | 262.28(179.26,384.25) | 4.45(3.82,5.08) |
|  | Male | 1732.71(337.98,4161.82) | 642.12(426.72,971.54) | 0.24(0.05,0.58) | 363.4(228.9,569.1) | 0.34(0.07,0.82) | 177.32(98.29,301.74) | 3.29(2.75,3.82) |
| Pakistan | Both | 286.13(63.27,659.35) | 446.22(292.23,719.59) | 0.13(0.03,0.29) | 175.07(97.53,312.74) | 0.31(0.07,0.71) | 206.63(121.71,361.35) | 4.22(3.17,5.28) |
|  | Female | 129.07(21.01,325.12) | 673.22(397.63,1056.14) | 0.12(0.02,0.3) | 280.29(144.75,468.62) | 0.29(0.05,0.72) | 296.96(154.63,493.47) | 5.29(3.5,7.11) |
|  | Male | 157.05(28.05,389.95) | 340.04(189.34,617.34) | 0.14(0.02,0.34) | 126.53(48.95,269.28) | 0.32(0.06,0.8) | 166.27(76.6,333.83) | 3.65(2.35,4.97) |
